# In vitro and in vivo evaluation of chemically synthesized, receptor-biased interleukin-4 and photocaged variants

**DOI:** 10.1126/sciadv.adw9755

**Published:** 2025-06-25

**Authors:** Mamiko Ninomiya, Cecilie Egholm, Daniel Breu, Onur Boyman, Jeffrey W. Bode

**Affiliations:** ^1^ Department of Chemistry and Applied Biosciences, ETH Zurich, 8093 Zurich, Switzerland.; ^2^ Department of Immunology, University of Zurich, University Hospital Zurich, 8091 Zurich, Switzerland.; ^3^ Center for Human Immunology (CHI), University of Zurich, 8006 Zurich, Switzerland.

## Abstract

Interleukin-4 (IL-4) plays a central role in type 2 immune responses. Despite its potential use for allergic and autoimmune diseases, its pleiotropic receptor binding complicates selective targeting of IL-4 signaling pathways. We developed a chemical synthesis of (i) IL-4 variants with atomically tailored side-chain modifications that deter specific receptor interactions and (ii) conditionally activatable IL-4 variants uncaged with 365-nanometer light. In primary cell studies, different variants elicited selective STAT5 or STAT6 phosphorylation in lymphocytes or neutrophils. In murine studies, photocaged IL-4 suppressed inflammation only upon UV irradiation, demonstrating precise on demand control. We accomplished the synthesis and folding of IL-4, a hydrophobic cytokine with three disulfide bonds, using the alpha-ketoacid–hydroxylamine (KAHA) ligation to assemble three segments. We introduced further conjugations, including PEGylation for half-life extension, through orthogonal ligations enabled by functionalized amino acid building blocks. This work highlights the flexibility of chemical protein synthesis to produce therapeutically valuable cytokines, including receptor-biased and spatiotemporally activatable IL-4 variants.

## Teaser

Chemically synthesized, receptor-biased IL-4 variants enable selective immune modulation and photo-controlled therapy.

## INTRODUCTION

Cytokines are a large class of small proteins that contribute to diverse cellular functions and cellular fates. Given their importance in inflammation and immune reactions, cytokines have been approved and used in the context of cancer immunotherapy since the 1980s (*1*). Despite their clinical potential and early successes, the further development of cytokine therapies is hampered by their short half-life, requiring multiple administrations that, in combination with their pleiotropic nature, lead to severe side effects. Strategies to specifically tailor functions and properties of cytokines to harness their full therapeutic potential are required (*2*). Recently, owing to the better understanding of cytokine immunobiology and structural biology, advances have been made by recombinant protein engineering to produce activity-modulated cytokines including muteins, conditionally activatable cytokines, superkines, computationally designed neoleukins, and orthogonal cytokine-receptor pairs (*3*–*7*).

**Fig. 1.**
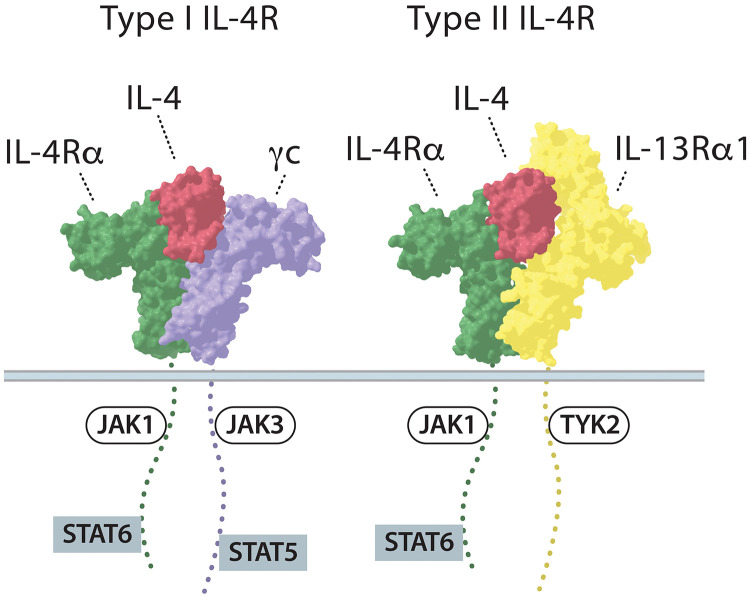
Schematic representation of IL-4 receptor system. The type I IL-4R consists of IL-4Rα and γc; the type II IL-4R is formed from IL-4Rα and IL-13Rα1. Each receptor is associated with a kinase which is a part of JAK-STAT signaling pathway.

Interleukin-4 (IL-4) is a classical four α-helical bundle cytokine that plays a central role in type 2 immune responses. IL-4 is involved in different immune processes, including differentiation of naive T cells to T helper 2 (T_H_2) cells, immunoglobulin (Ig) class switching to IgE, dendritic cell maturation, and macrophage activation (*4*). IL-4 confers its pleiotropic actions by engaging two types of heterodimeric IL-4 receptor (IL-4R) complexes on cell surfaces. The type I IL-4R is comprised of IL-4Rα and γc, while type II IL-4R is formed by IL-4Rα and IL-13Rα1 (Fig. 1). In both IL-4R complexes, IL-4 primarily binds to IL-4Rα with high affinity (*K*_d_ = ~10^−10^ M), after which IL-4–IL-4Rα recruits the low-affinity receptor subunit, either γc or IL-13Rα1 [*K*_d_ (dissociation constant) = ~10^−6^ M] (*8*). Type II IL-4R can also be bound by IL-13, which rationalizes the observation that IL-4 and IL-13 have overlapping biological activities in certain cell types. After forming heterodimeric complexes, both type I and II IL-4Rs activate the intracellular Janus kinase (JAK)–signal transducers and activators of transcription (STAT) signaling pathway by phosphorylating JAK1, JAK3, TYK2, STAT5, and/or STAT6, after which phosphorylated STAT (pSTAT) dimers translocate into the nucleus. While IL-4Rα is expressed widely over different cell types, the expression of the second low-affinity receptor subunits γc and IL-13Rα1 is highly dependent on the cell types. γc is mainly expressed in hematopoietic cells, such as T and B cells, whereas IL-13Rα1 is expressed in non-hematopoietic cells and some myeloid cells (*8*–*13*).

Despite its important role in type 2 immune responses, dysregulated IL-4 is implicated in the pathogenesis of allergic diseases, which affect large parts of the global population. Because IL-4 signaling is involved in the essential steps of the development and evolution of allergic diseases, blocking IL-4 signaling is regarded as an ideal target for the prevention and treatment of allergic diseases (*10*, *14*). Previously, efforts have been made to engineer IL-4 mutants and IL-4 conjugates that act as an antagonist by preventing the binding of the second receptors (*15*–*17*). Dupilumab, an anti–IL-4Rα monoclonal antibody, blocks IL-4R–mediated signaling and was approved for atopic dermatitis in 2017 and for asthma in 2018 by the Food and Drug Administration (*18*–*20*).

While overproduction of IL-4 is an undesired element in atopic disease, IL-4 remains a potential treatment for autoimmune diseases such as psoriasis. Neutrophils are tightly involved in the development and progression of psoriasis by leading to inflammation, attracting other inflammatory immune cells, and forming neutrophil extracellular traps (NETs), which altogether lead to the destruction of healthy tissue (*21*, *22*). Recent studies showed that IL-4R signaling in neutrophils has an inhibitory effect by curtailing neutrophil migration, phagocytosis, and NET formation and by accelerating their cellular aging (*11*, *13*, *23*–*25*), explaining the increasing interests in using engineered IL-4 variants as potential treatments for patients with neutrophilic autoimmune and autoinflammatory disease (*26*, *27*).

To shepherd IL-4 function toward a specific disease area, it is advantageous to modulate its properties and binding affinities by atomic tailoring of specific side chains. Here, we describe the chemical synthesis of IL-4 using the α-ketoacid–hydroxylamine (KAHA) amide-forming ligation (*28*, *29*). This modular synthetic route enabled us to generate IL-4 variants that (i) block or differentiate between the two IL-4R complexes and (ii) serve as half-life extended photocaged IL-4R agonists for spatiotemporally controlled treatment of mouse models of neutrophilic inflammation. This was achieved by using chemical protein synthesis to incorporate multiple noncanonical amino acids bearing unique functional groups, including photoactivatable amino acid and conjugation handles. Together, our data demonstrate the facile modulation and manipulation of bioactive molecules using a chemical synthesis platform for cytokine engineering to control receptor binding and selectivity.

## RESULTS

### Synthesis of mouse IL-4 wild type

**Fig. 2.**
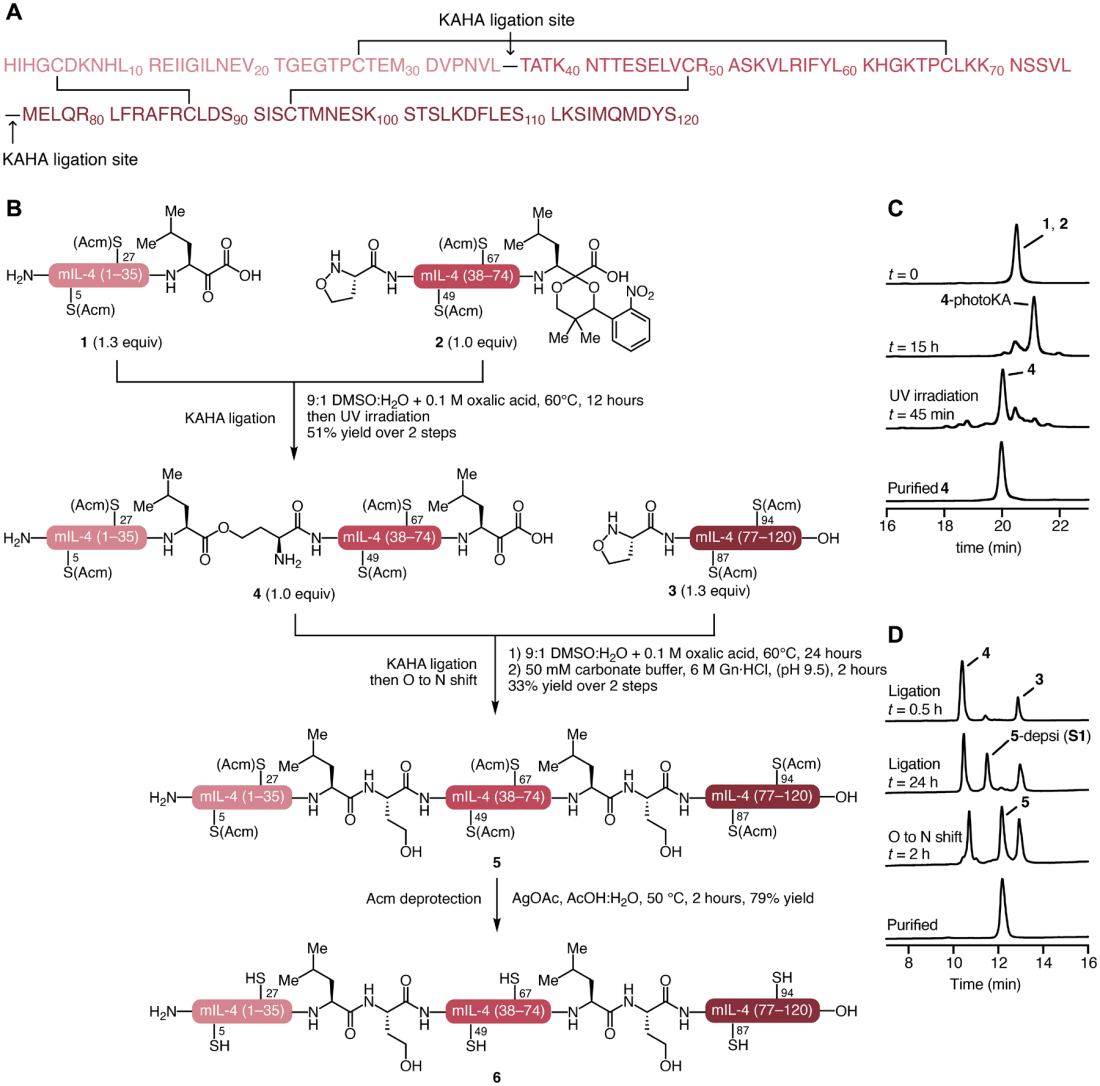
Modular chemical synthesis of IL-4 wild type using KAHA ligation. (**A**) Amino acid sequence of mouse IL-4 showing the ligation sites. (**B**) Synthesis of linear IL-4 **6** by sequential KAHA ligations. (**C**) Analytical HPLC monitoring of ligation between segment **1** and **2** to yield peptide **4**. (**D**) Analytical HPLC monitoring of ligation between peptide **4** and **3** to obtain polypeptide **5**.

While IL-4 is prevalent in all mammals, the sequence divergence is high among different species (*30*). For example, mouse and human IL-4 have sequence identity of only 41% and do not exhibit cross-reactivity. Although most previous antagonist development studies were based on human IL-4, we decided to focus on the mouse system to facilitate in vivo studies. Mouse IL-4 consists of 120 amino acids including six cysteines, all of which are involved in disulfide bonds (Cys5-Cys87, Cys27-Cys67, and Cys49-Cys94) (*31*, *32*). From the primary sequence, we envisioned synthesizing IL-4 from three segments using two KAHA ligations (Fig. 2A). We chose the ligation sites to be Leu36-Thr37 and Leu75-Met76. The most convenient implementation of KAHA ligation uses 5-oxaproline as the hydroxylamine, as this building block is readily available and forms more soluble peptide esters as the primary ligation product. This building block introduces a noncanonical homoserine residue at the ligation site, and we identified the Thr37Hse and Met76Hse mutations as being unlikely to interfere with receptor interactions. We used temporary acetaminomethyl (Acm) group (*33*) as orthogonal protecting groups for the cysteines to avoid oxidation and undesired disulfide scrambling during the synthesis; likewise, we replaced methionine residues with norleucines to avoid oxidation.

Each peptide segment was prepared by Fmoc solid-phase peptide synthesis (SPPS). We synthesized C-terminal α-ketoacid–bearing peptide segments **1** and **2** using our established standard protocol for peptide α-ketoacids (*29*). For orthogonality during the KAHA ligation, the α-ketoacid in the middle segment **2** was protected with a photolabile group. Cleavage of the peptides from the resin and purification by reverse phase high-performance liquid chromatography (RP-HPLC) afforded desired peptide segments **1** and **2** in good yield (18 and 21% yield, respectively). The synthesis of C-terminal segment **3** proved challenging due to its hydrophobicity. After screening multiple resins and dipeptides, the use of polyethylene glycol (PEG)–based HMPB ChemMatrix resin and introduction of two pseudoprolines (*34*) at Ile92-Ser93 and Ser101-Thr102 during the synthesis afforded us the desired peptide **3**, albeit in somewhat low yield (3.3% overall yield based on the loading of the resin). Despite its poor solubility and chromatographic recovery, this route provided ample amounts of this segment for our studies.

We assembled the linear protein from the synthetic segments in a sequential manner (Fig. 2B). We ligated segments **1** and **2** under standard KAHA ligation conditions (20 mM, 9:1 dimethyl sulfoxide (DMSO):H_2_O containing 0.1 M oxalic acid, 60°C) followed by ultraviolet (UV) irradiation to remove the α-ketoacid protecting group; we purified the product by RP-HPLC to obtain **4** in 51% yield (Fig. 2C). We coupled peptide **4** with segment **3** by KAHA ligation using the same conditions to yield linear IL-4 containing two homoserine depsi-peptides, which are the primary product of KAHA ligations with 5-oxaproline. We directly rearranged these ester bonds to amide bonds by diluting the ligation solution with a basic buffer [50 mM sodium carbonate and 6 M Gn·HCl (pH 9.5)] to give linear protein **5** in 33% yield over two steps (Fig. 2D). We removed the Acm protecting groups by the treatment with AgOAc in AcOH/water for 1 hour at 50°C, affording linear IL-4 (**6**) in 79% yield.

**Fig. 3.**
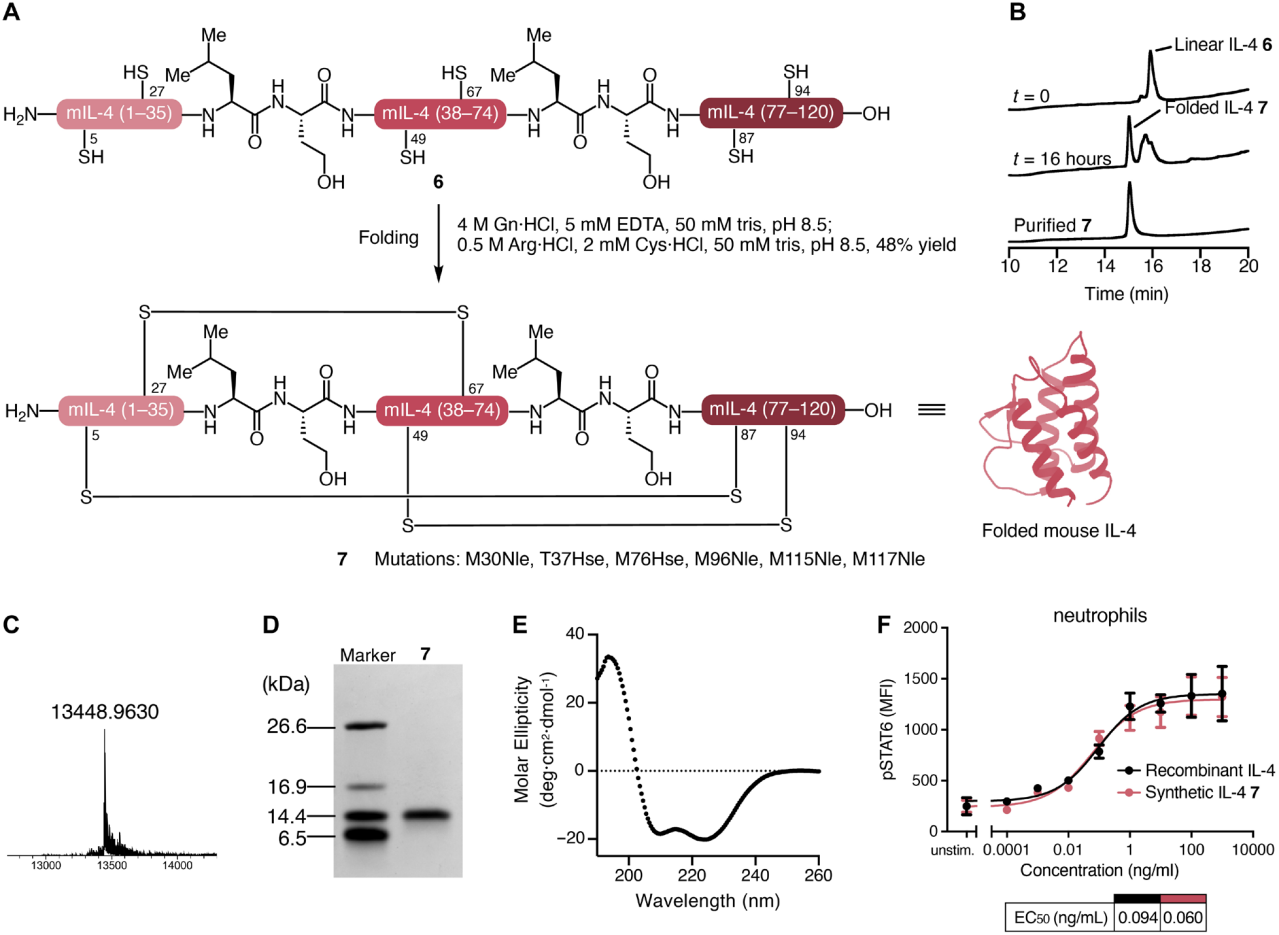
Folding of linear IL-4 wild type and characterization of smIL-4. (**A**) Folding of synthetic IL-4. (**B**) Analytical HPLC monitoring of folding. (**C**) ESI-HRMS of folded smIL-4 **7**. (**D**) SDS-PAGE of folded smIL-4 **7**. (**E**) CD spectrum of folded smIL-4 **7**. (**F**) Dose-dependent STAT6 phosphorylation by recombinant mIL-4 (rIL-4) and smIL-4 **7** in neutrophils. *n* = 2. For the assay with T cells and B cells, see fig. S2.

Initial attempts to fold **6** based on the reported conditions for recombinantly expressed mouse IL-4 did not yield sufficient quantities of the folded protein (*31*). After optimization of denaturant, pH, concentration, and redox reagents, we identified suitable conditions: A lyophilized powder of **6** was dissolved in buffer A [6 M Gn·HCl, 40 mM tris, and 1 mM EDTA (pH 8.5)] at a concentration of 0.5 mg/ml. We diluted the protein solution six times in the buffer B [0.5 M Arg·HCl, 40 mM tris, and 5 mM Cys (pH 8.5)] and incubated overnight at room temperature (Fig. 3A). After acidification, we purified the folded IL-4 **7** successfully by RP-HPLC with 48% isolated yield (Fig. 3B). We confirmed the mass and purity of folded synthetic IL-4 by electrospray ionization high-resolution mass spectrometry (ESI-HRMS), SDS–polyacrylamide gel electrophoresis (SDS-PAGE), and RP-HPLC (Fig. 3, B to D). In addition, the circular dichroism (CD) spectrum of **7** showed a distinct α helix pattern (Fig. 3E).

To test the stimulatory activity of synthetic mouse IL-4 (smIL-4, **7**), we assessed its ability to induce pSTAT6 and pSTAT5 in neutrophils, T cells, and B cells isolated from mouse spleen (Fig. 3F and figs. S1 and S2). In neutrophils, smIL-4 **7** successfully induced pSTAT6, reaching comparable median effective concentration (EC_50_) values as recombinant IL-4 (rIL-4) (Fig. 3F). While neutrophils are shown in Fig. 3F as a representative example, we observed comparable activation profiles and EC_50_ values across all tested cell types and STAT pathways (fig. S2). Together, the biological activity and characterization were consistent with successful synthesis and folding of smIL-4; the mutations (M30Nle, T37Hse, M76Hse, M96Nle, M115Nle, and M117Nle) did not affect the biological activity.

### Synthesis of IL-4Rα antagonist and activity-modulated IL-4 variants

**Fig. 4.**
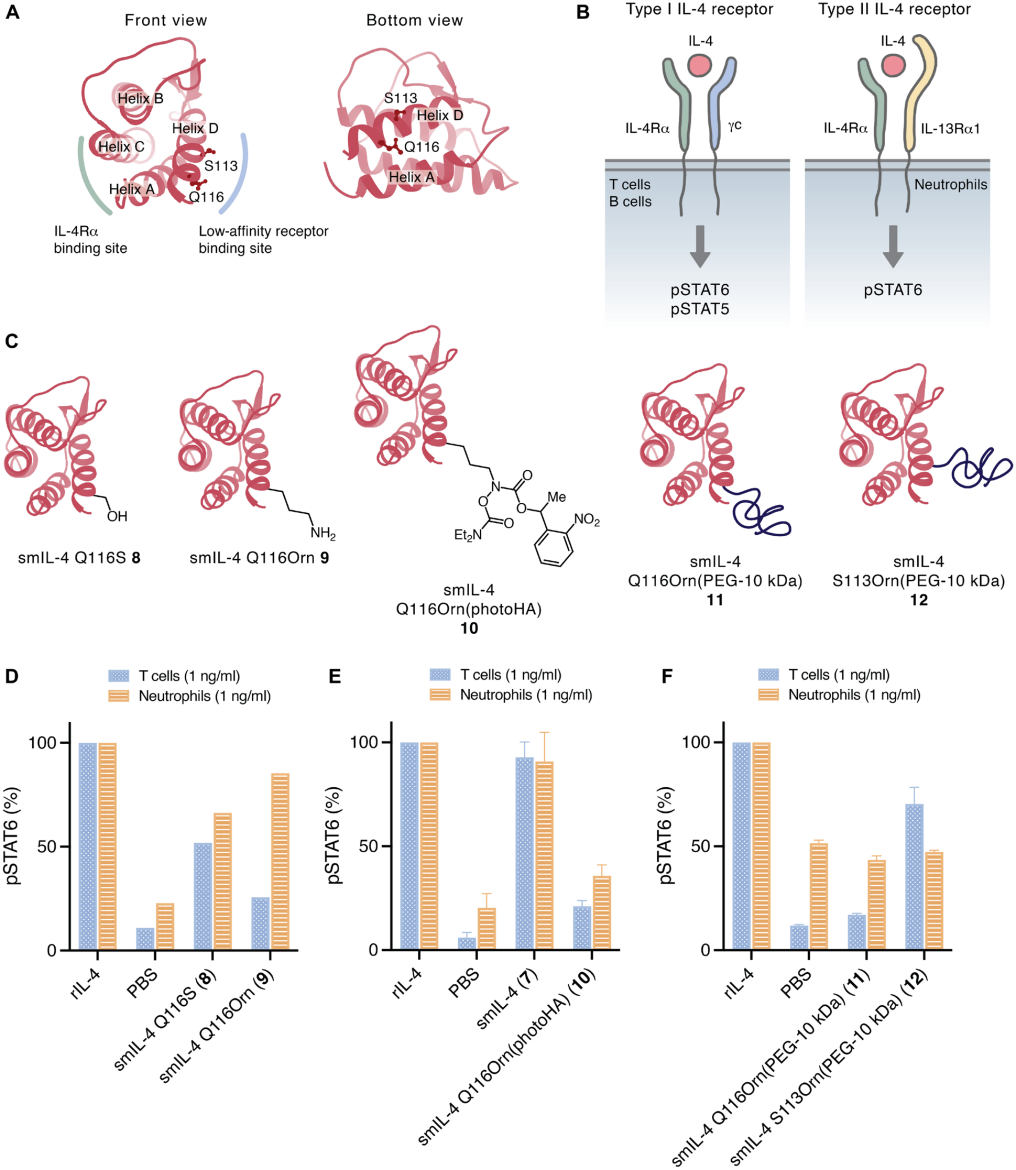
Design and biological activity of IL-4 variants as IL-4Rα antagonist and activity-modulated IL-4 variants. (**A**) Estimated structure of mouse IL-4 and chosen amino acid residues to introduce PEG groups located at receptor-binding sites. (**B**) Scheme showing the IL-4 receptor signaling system and corresponding STAT proteins that are phosphorylated upon receptor engagement by cytokine. (**C**) Synthesized and tested IL-4 variants. (**D** to **F**) In vitro dose-dependent STAT6 phosphorylation by rIL-4, smIL-4 **7**, smIL-4 Q116S **8**, smIL-4 Q116Orn **9**, smIL-4 Q116Orn(photoHA) **10**, mIL-4 Q116Orn(PEG-10 kDa) **11**, and mIL-4 S113Orn(PEG-10 kDa) **12** at a concentration of 1 ng/ml in T cells and neutrophils. The concentration is based on the protein part (IL-4) of the molecule. *n* = 1 (D) and *n* = 2 (E and F). For the complete dose-dependent assay, see figs. S2 to S4.

With an effective synthesis of wild-type smIL-4 established, we adapted this synthetic route to the production of unique, defined mIL-4 variants. First, we aimed to synthesize smIL-4 that have modulated activity to the IL-4R complex by blocking the IL-4R signaling, a modality that could be potentially used to treat allergic disease. To engineer such a protein, we designed mutants and PEGylated smIL-4 constructs that should retain binding to the high-affinity IL-4Rα, but with attenuated or abrogated binding to the second receptor subunits γc or IL-13Rα1. Because the structure of mIL-4 has not been reported to date, we selected the modification sites to be Q116 based on the predicted structure generated by Swiss model (Fig. 4A), the human IL-4 (hIL-4) crystal structure complexed with receptors [Protein Data Bank (PDB): 3BPL and 3BPN], and sequence alignment of hIL-4 and mIL-4. Based on the synthetic route to wild-type smIL-4, we synthesized five different variants **8** to **12** and tested in vitro (Fig. 4C). We chose the mutations Q116S (**8**) and Q116Orn (**9**) to test the effect of slight differences in the structure compared to Gln116. In addition, we synthesized the PEGylated variant smIL-4 Q116Orn(PEG-10 kDa) **11** to block binding of the second receptor subunit using potassium acyltrifluoroborate (KAT) conjugation reaction (*35*, *36*), and the photoprotected intermediate before the ligation smIL-4 Q116Orn(photoHA) **10** was also tested in vitro. To assess the role of the site of PEGylation, we prepared variant S113Orn(PEG-10 kDa) **12**.

Because IL-4R complex components expression differs between cell types, the receptor associated STAT signaling also varies between cell types. As shown in fig. S1, lymphocytes (T cells and B cells) predominantly express type I IL-4R (IL-4Rα and γc), which activate both STAT5 and STAT6 upon ligand binding (Fig. 4B). In contrast, neutrophils primarily express type II IL-4R (IL-4Rα and IL-13Rα1) and signal mainly through STAT6 (Fig. 4B). (*8*) smIL-4 Q116S mutant **8** had an overall loss of 50 to 70% pSTAT5 and pSTAT6 in T cells and neutrophils and did not show any cell type selectivity (Fig. 4D and fig. S3). In contrast, IL-4 Q116Orn mutant **9** interestingly had substantially reduced activity in T cells while maintaining comparable activity to rIL-4 in neutrophils (Fig. 4D and fig. S3). This implied that the Q116Orn mutation biased IL-4 binding against γc without affecting binding to IL-13Rα1. Introducing a bulky small molecule at Q116, rather than a mutation (variant **10**), reduced pSTAT in both T cells and neutrophils (Fig. 4E and fig. S2). Introduction of an even bulkier PEG group (PEG-10 kDa) at Q116 (variant **11**) reduced pSTAT6 to the level seen with PBS in both T cells and neutrophils (Fig. 4F and fig. S4). Intriguingly, introducing this PEG group at S113 (variant **12**) selectively affected pSTAT6 in neutrophils, but not in T cells, resulting in the opposite effect of variant **9** (Fig. 4F and fig. S4). An explanation for this difference could be that position S113 or its proximate environment is particularly important for the binding with IL-13Rα1, which is used for type II IL-4Rs in neutrophils, whereas Q116 and its surrounding environment is important for both the binding with γc and IL-13Rα1. Further investigation of smIL-4 Q116Orn(PEG-10 kDa) **11** by surface plasmon resonance (figs. S5 and S6) showed that variant **11** retained the binding ability to the IL-4Rα, but it is not capable to bind to the second receptor γc. In vitro competition assay against rIL-4 (fig. S7) supported the notion that this variant **11** could act as an antagonist. While the variant **11** (1000 ng/ml) could successfully diminished pSTAT5 in both T cells and B cells, it reduced the pSTAT 6 in neutrophils, T cells, and B cells only 40 to 70% compared to the reference without any variant **11** added.

### Design, synthesis, and in vitro assessment of photocaged IL-4

**Fig. 5.**
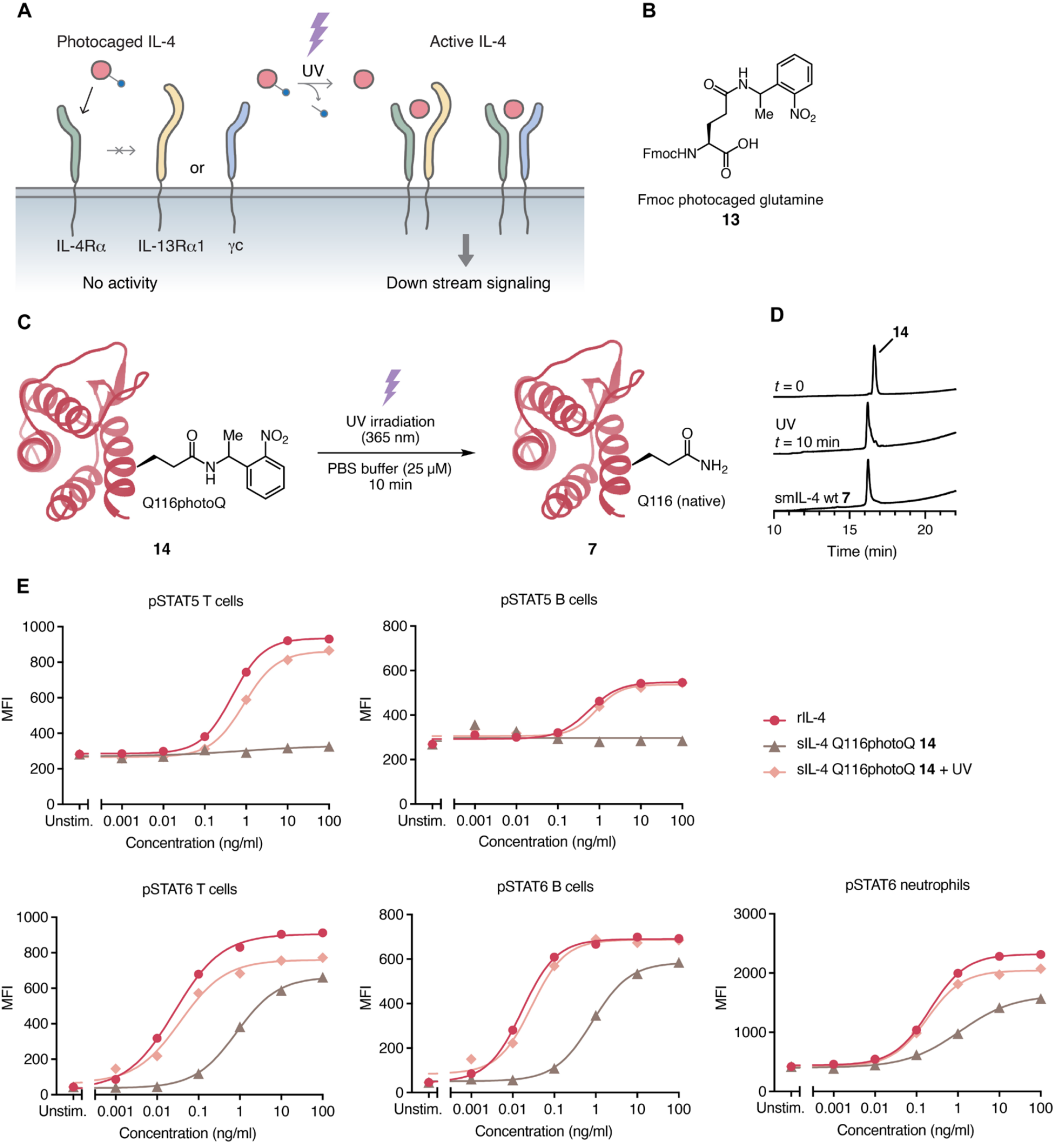
Design and photoinduced activity of photocaged IL-4. (**A**) Concept of photocaged IL-4. (**B**) Photocaged glutamine as an Fmoc SPPS monomer. (**C** and **D**) Deprotection of synthesized photocaged IL-4 and HPLC trace. (**E**) Dose-dependent STAT phosphorylation by rIL-4 and photocaged IL-4 **14** with or without UV irradiation for 10 min in T cells, B cells, and neutrophils. *n* = 1.

The high potency and pleiotropic activities of cytokines can limit their therapeutic utility. To mitigate this, conditional activation strategies are in high demand and successful approaches include protease activation (*37*–*39*) and reducing potential- and pH-dependent exposure of cytokines (*40*). The ability to chemically synthesize IL-4 opens new avenues for conditional activation, as caging groups that cannot be installed in recombinant cytokines can be easily introduced. As we observed that Q116 in mIL-4 is crucial for binding to the secondary low-affinity receptor subunit, we envisioned to synthesize smIL-4 with a photocleavable group at Q116, which could still bind to IL-4Rα; however, the second receptor subunit would not be recruited, abrogating the down-stream signaling until the photocaging group is removed by irradiation (Fig. 5A). Such an IL-4 variant could be of great interest to treat certain organ-specific autoimmune diseases, such as rheumatoid arthritis and psoriasis, by precisely controlling timing and location of activation without inducing systemic adverse side effects.

We embarked on photocaged IL-4 synthesis by designing and synthesizing photocaged glutamine **13** (Fig. 5B) as described in the Supplementary Materials based on reported examples of photocaged amides (*41*–*43*). We incorporated synthesized **13** during SPPS and following our established modular synthetic route toward IL-4 provided us an IL-4 variant with photocaged glutamine at Q116 **14** (photoQ).

We subjected folded, photocaged smIL-4 **14** to photo-uncaging tests (Fig. 5C). We irradiated a solution of **14** in PBS with 365-nm UV light. Ten minutes of UV irradiation removed the photocaging group to reveal the native glutamine residue, as observed by RP-HPLC; the major peak corresponded to that of wild-type smIL-4 **7** (Fig. 5D) and exhibited the expected mass of smIL-4 **7** by HRMS.

We tested the biological activity of photocaged smIL-4 **14** before and after UV decaging by pSTAT assay (Fig. 5E). As expected, before UV irradiation, **14** did not effectively induce signaling and failed to show any signaling activity in T cells, B cells, or neutrophils. However, UV irradiation completely restored the activity to that of rIL-4. We attributed the small residual activity for STAT6 phosphorylation before UV irradiation to the small size of the photocaging group, which may not fully inhibit the formation of the receptor binding complex.

### In vivo assessment of PEGylated photoQ IL-4

**Fig. 6.**
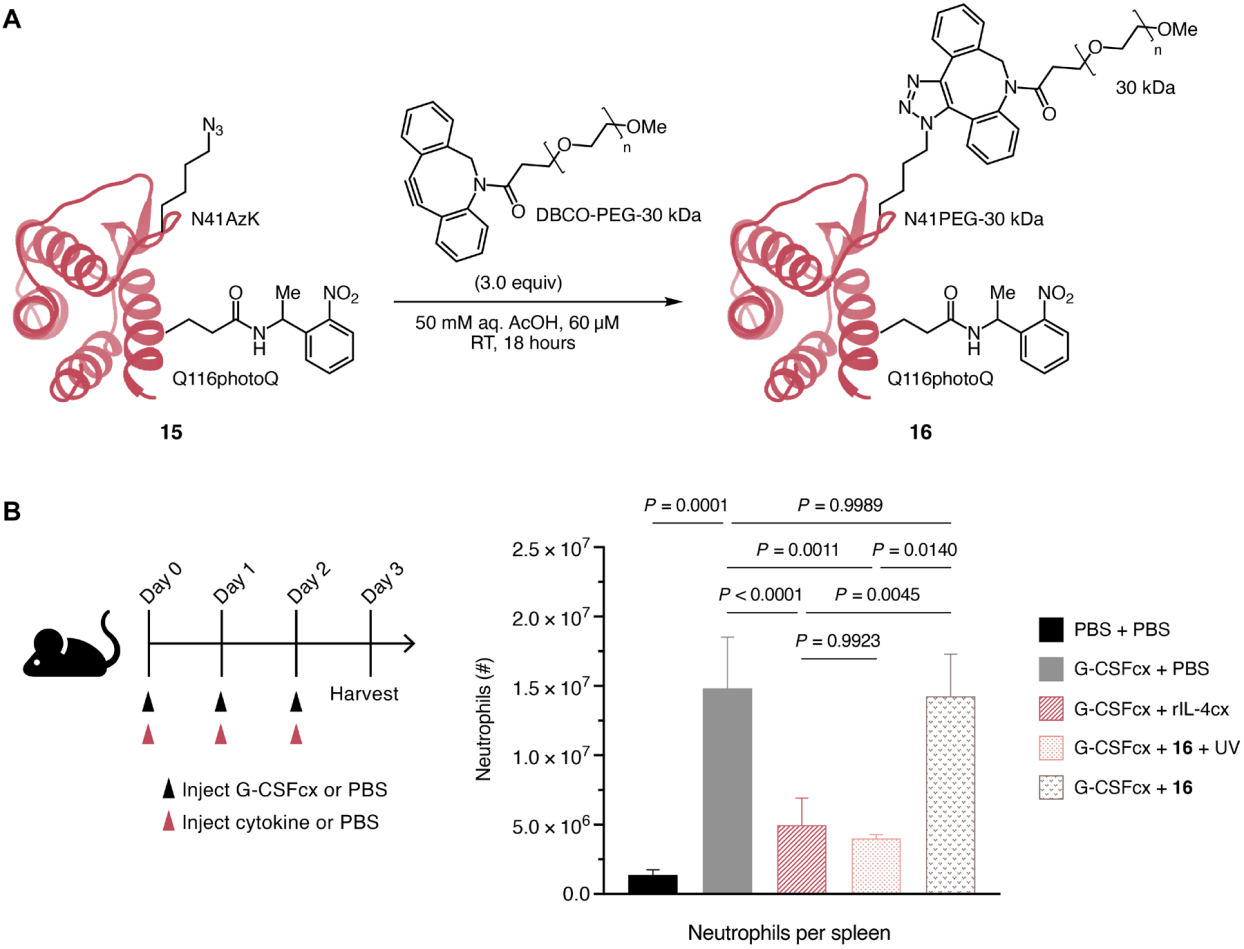
Synthesis and biological activity of PEGylated photocaged IL-4. (**A**) PEGylation of photocaged IL-4 with azidolysine at N41 **15** using SPAAC reaction (only one regioisomer is shown). (**B**) In vivo assay of photocaged IL-4 **16**. We injected mice with half-life extended G-CSF/anti–G-CSF mAb complexes (G-CSFcx, 1 μg/6 μg) and rIL-4/anti–IL-4 mAb complexes (rIL-4cx, 1.5 μg/15 μg) or PEGylated photocaged IL-4 **16** (5 μg) with or without UV irradiation for 10 min before injection. We euthanized mice on day 3, and neutrophil counts were determined in the spleen by flow cytometry. The concentration is based on the protein part (IL-4) of the molecule. *n* = 8 (G-CSFcx + PBS and G-CSFcx + rIL-4cx) and *n* = 2 (PBS + PBS, G-CSFcx +**16** + UV, G-CSFcx +**16**). We analyzed statistical significance using one-way analysis of variance (ANOVA) with Tukey’s correction for multiple comparisons.

With these promising results in vitro, we proceeded with in vivo experiments in mice. Because of its small size, IL-4 has a short half-life in vivo. Half-life extension strategies include complexation of IL-4 with a particular anti–IL-4 monoclonal antibody (mAb), resulting in IL-4/anti–IL-4 mAb complexes (*11*), fusion to Fc fragments, or conjugation with half-life extending polymers. By using our flexible and modular chemical synthesis of IL-4, we prepared a PEGylated, photocaged IL-4. Introducing a PEG group is a well-established strategy to extend in vivo half-life of therapeutic proteins by increasing the molecular weight and hydrodynamic radius of the molecule (*44*, *45*). To this end, we introduced an azidolysine at position N41, which is located in the loop between helices A and B. We coupled this azidolysine with a dibenzocyclooctyne containing a PEG group via strain-promoted alkyne azide cycloaddition (SPAAC) to afford PEGylated photocaged smIL-4 **16** (Fig. 6A) (*46*, *47*). It should be noted that we did not use the previously used KAT ligation in this particular case because the photoprotecting group of hydroxylamine amino acid is not orthogonal to photocaged glutamine **13**. The synthesis, folding, and characterization of the half-life-extended, photocaged smIL-4 **16** proceeded as expected.

We tested the activity of PEGylated photocaged smIL-4 **16** by inducing systemic neutrophilia by injection of recombinant mouse granulocyte colony-stimulating factor (G-CSF) and measuring whether **16** reduced blood counts of neutrophils by IL-4R engagement on neutrophils, as previously published (*11*, *48*). We observed that PEGylated, photocaged smIL-4 **16** was unable to inhibit G-CSF–induced neutrophilia, as evidenced by neutrophil counts that were as high as in G-CSF–injected control mice treated with PBS. However, when we treated the PEGylated photocaged IL-4 with UV light before injection, neutrophil counts were suppressed comparable to when using half-life-extended rIL-4/anti–IL-4 mAb complexes. This demonstrates that controlled activation of IL-4 using our photocage was successful. Moreover, PEGylation of photocaged IL-4 successfully extended the half-life of IL-4, and the biological activity of IL-4 was seen in vivo without complexation to a mAb or another reagent. These data illustrate the potential of synthetic IL-4 variants to serve as conditionally activated cytokines.

## DISCUSSION

Cytokines are critical mediators of immune responses, and they hold promise as next-generation therapies for various diseases. Their clinical use, however, is currently limited due to the lack of precise engineering methods to improve their short half-life and pleiotropism. Engineering of cytokines to modulate or emphasize the desired activity is essential to harness their therapeutic potential.

In this study, we have established a chemical synthesis of mIL-4 and demonstrated that this modular synthetic route can be used to produce two functionally different IL-4 variants: (i) activity modulated IL-4 variants that can block or differentiate between the two IL-4R types and (ii) photocaged IL-4 that acts as a spatiotemporally controlled agonist.

In addition to preparation of smIL-4 Q116Orn(PEG-10 kDa) **11**, an IL-4Rα antagonist with a PEG group at Q116 position, we found an important feature of some variants. The Q116Orn mutant **8** did not notably affect the bioactivity in neutrophils, but this variant was not active in T cells. Contrarily, smIL-4 S113Orn(PEG-10 kDa) **12** selectively inhibited signaling only in neutrophils, while retaining activity in T cells. This information can be used to fine tune these residues and bias the binding to the two different secondary receptor subunits, with which they share the binding surface on mIL-4. Therefore, selective activation of one of these receptor complexes is possible. Recent work by Yang *et al.* (*7*) demonstrated that receptor-biased IL-4 can be generated through computational de novo protein design, in which the entire amino acid sequence was redesigned to selectively engage the type I IL-4R. While this innovative study highlights the possibilities of computational design, our results show that similar receptor-selective modulation can be also achieved through simple, site-specific residue modifications. Our chemical approach retains the native IL-4 sequence and structure, introducing only minimal modifications to tune receptor binding.

These findings, which had not been previously observed by using recombinantly generated muteins of mIL-4, in principle enable the development of an antagonist variant of IL-4 selective to type II IL-4R. The high concentration needed for pSTAT inhibitory with smIL-4 Q116Orn(PEG-10 kDa) **11** may reflect the fact that native IL-4 forms a more stable and complete IL-4R, enabling it to outcompete variant **11** and initiate signaling from internalized complexes in cortical endosomes (*49*–*51*). In addition, at these high concentrations, variant **11** retains residual pSTAT activity, contributing the modest pSTAT6 inhibition observed.

In the second application of the chemical synthesis of mIL-4, we generated a multifunctional mIL-4 containing two unnatural amino acids. We replaced Q116, which proved to be an effective position to modulate binding to the secondary receptor subunit, with a photocaged glutamine residue that recovered its structure after UV irradiation. While half-life extension in blood circulation of photocaged smIL-4 was required to facilitate the biological activity in vivo, we successfully conjugated an additional PEG group to photocaged IL-4 by using SPAAC reaction for half-life extension, demonstrating the ease at which new types of multifunctional cytokines can be designed and engineered by using chemical protein synthesis.

For this proof-of-concept study of a photocaged IL-4 we prepared a photocaged Gln which required UV-A irradiation to remove the protecting group. While we could successfully demonstrate suppression of G-CSF–mediated neutrophil expansion in mice upon decaging the photocaged IL-4, the use of this strategy is limited owing to the challenges associated with administering high-intensity UV-A light. The successful demonstration of this, however, lays the groundwork for alternative conditional activation approaches including photo-caging group at longer wavelengths or chemically or enzymatically induced uncaging (*52*, *53*).

Collectively, chemical protein synthesis enabled us to access a variety of therapeutically valuable IL-4 variants that could not have been obtained otherwise. By establishing wild-type IL-4 synthesis as a platform, we could easily introduce almost any modifications at will to obtain differently functionalized proteins in combination with other organic chemistry methodologies. This concept can be extended to engineer other cytokines with selective functions for use as potential therapeutics.

## MATERIALS AND METHODS

### Animals

C57BL/6 (referred to as B6) mice were purchased from Charles River Laboratories. Animal experiments were performed with 2- to 5-month-old female mice in accordance with the Cantonal Veterinary Office of Zurich and with Swiss Federal and Cantonal Law (authorization no. ZH199/2021).

### Phosphorylated STAT signaling assay

Spleen isolated from C57BL/6 mice were processed into single-cell suspension and resuspended in RPMI 1640 medium (Gibco) supplemented with 5% fetal bovine serum (FBS; Thermo Fisher Scientific). The cells were left to rest for 1 hour in an incubator (always at 37°C with 5% CO_2_). Hereafter, the bulk splenocytes were stimulated with varying concentrations of recombinant mouse IL-4 (rIL-4; PeproTech), synthetic IL-4, different variants of PEGylated IL-4s or IL-4 variants for 15 min in an incubator as indicated on the figures. For the competition assay, the PEGylated IL-4 variants were mixed with a fixed concentration of rIL-4 (1 ng/ml) before stimulation of cells. Afterward, the cells were fixed with fix buffer I (BD Phosflow), followed by permeabilization with perm buffer III (BD Phosflow) according to the manufacturer’s protocol. Phosphorylation of intracellular STATs was analyzed by flow cytometry.

### Flow cytometry

Cells were stained for analysis by flow cytometry using fluorochrome-conjugated monoclonal antibodies directed against the following mouse antigens: CD3 (145-2C11, BD Biosciences), CD4 (GK1.5, eBioscience), CD11b (M1/70, BioLegend), CD19 (6D5, Biolegend), CD25 (7D4, eBioscience), CD44 (IM7, Biolegend), CD62L (MEL-14, BD Biosciences), CD124 (mIL4R-M1, BD Biosciences), CD132 (TUGm2, BioLegend), CD213a1 (13MOKA, Invitrogen), IgG2a (eBioscience), IgG2b (eBioscience), Ly6G (1A8, BioLegend), IL-4 (11B11, Invitrogen), IL-17A (eBio17B7, eBioscience), INFg (XMG1.2; eBioscience), major histocompatibility complex (MHC) II (M5/114.15.2; eBioscience), pSTAT3 (Tyr705; 13A3-1, BioLegend), pSTAT5 (Tyr694; 47/Stat5(pY694), BD Phosflow), and pSTAT6 (Tyr641; J71-773.58.11, BD Phosflow). Cells were acquired on a BD LSR Fortessa flow cytometer and analyzed using FlowJo software (Tristar).

### Sterile inflammation model

B6 mice received intraperitoneal injections for three consecutive days of G-CSF/anti–G-CSF complexes (cx), made of 1 μg of human G-CSF (Neupogen, Amgen) complexed with 6 μg of anti-human G-CSF antibody (BVD11-37G10, SouthernBiotech) and, for IL-4cx, 1.5 μg of mouse IL-4 (PeproTech) complexed with 15 μg of anti-IL-4 antibody (11B11, Bio X Cell) or indicated amount of uncomplexed cytokine. Mice were euthanized 16 hours after the last injection and spleen, bone marrow, and blood were processed into single-cell suspensions for analysis by flow cytometry. Cell surface staining of 2 × 10^6^ cells was performed for 30 min at 4°C with the following antibodies: BUV395-conjugated anti-CD11b (M1/70, BD Biosciences), BUV737-conjugated anti-CD3 (17A2, BD Biosciences), ef450–conjugated anti-Ly6C (HK1.4, Thermo Fisher Scientific), BV510-conjugated anti-CD19 (6D5, BioLegend), BV711-conjugated anti-NK1.1 (PK136, BioLegend), BV785-conjugated anti-CD11c (N418, BioLegend), fluorescein isothiocyanate–conjugated anti-MHC II (M5/114.15.2, BioLegend), phycoerythrin-conjugated anti-XCR1 (ZET, BioLegend), allophycocyanin-conjugated anti-Ly6G (1A8, BioLegend), and Fixable Viability Dye eFluor 780 (Thermo Fisher Scientific). Samples were acquired on a BD LSR II flow cytometer (BD Biosciences) and analyzed using FlowJo software (BD Biosciences).

### General procedures for Fmoc SPPS

The following Fmoc-amino acids with side-chain protecting groups were used: Fmoc-Ala-OH, Fmoc-Arg(Pbf)-OH, Fmoc-Asn(Trt)-OH, Fmoc-Asp(OtBu)-OH, Fmoc-Cys(Acm)-OH, Fmoc-Gln(Trt)-OH, Fmoc-Glu(OtBu)-OH, Fmoc-Gly-OH, Fmoc-His(Trt)-OH, Fmoc-Ile-OH, Fmoc-Leu-OH, Fmoc-Lys(Boc)-OH, Fmoc-Nle-OH, Fmoc-Phe-OH, Fmoc-Pro-OH, Fmoc-Ser(tBu)-OH, Fmoc-Thr(tBu)-OH, Fmoc-Trp(Boc)-OH, Fmoc-Tyr(tBu)-OH, and Fmoc-Val-OH.

### Automated Fmoc SPPS

Peptides were synthesized on a CS Bio 136X synthesizer or on a Multisyntech Syro I parallel synthesizer or on a Symphony X multiple peptide synthesizer using Fmoc-SPPS chemistry. Fmoc deprotections were performed with 20% (v/v) piperidine in *N*,*N*′-dimethylformamide (DMF) (10 min × *n*) and washed with DMF (×5). Unless stated, couplings were performed with Fmoc-Xaa-OH (4.0 equiv relative to resin loading), HCTU (3.95 equiv) and *N*-methylmorpholine (NMM; 8.0 equiv) in DMF (45 min × 2) and washed with DMF (×5). After coupling, unreacted free amine was capped by treatment with a mixture of acetic anhydride/NMM/DMF (1:1:4, v/v, 5 min × 2) and washed with DMF (×5). Fmoc-Cys(Acm)-OH (4.0 equiv relative to resin loading) was coupled by using 6-Cl HOBt (4.0 equiv) and diisoproprylcarbodiimide (DIC; 4.0 equiv) in DMF for 2 hours (×1 unless stated). Pseudoprolines (2.0 equiv relative to resin loading) were coupled by using HATU (1.95 equiv) and NMM (4.0 equiv) for 2 hours (×1 unless stated).

### Resin cleavage procedures

The dried resin was placed in a glass vial, the trifluoroacetic acid (TFA) cleavage cocktail [dried resin (20 ml/g)] was added, and the suspension was shaken for 1.5 to 2 hours. The resin was filtered, and the volatiles were removed under reduced pressure. The residue was triturated with Et_2_O [dried resin (~15 ml/g)] and centrifuged (3500*g*, 5 min), and the supernatant was removed by decantation. This trituration/washing step was repeated twice. The crude material was dried and dissolved in a suitable solvent [DMSO or H_2_O/CH_3_CN (1:1, v/v) containing 0.1% (v/v) TFA] for RP-HPLC purification. The following TFA cleavage cocktail compositions were used: cleavage cocktail A: TFA:DODT:H_2_O (95:2.5:2.5, v/v/v) and cleavage cocktail B: TFA:TIPS:H_2_O (95:2.5:2.5, v/v/v).

### Synthesis of IL-4 wild-type 7

#### Segment synthesis

H-[Cys(Acm)5,27]–IL-4(1–35)–Leu–α-ketoacid **1** was synthesized on Rink aide polystyrene resin loaded with Fmoc-protected Leu–α-ketoacid precursor (2.0 g, 0.22 mmol/g, 0.44 mmol). After automated Fmoc-SPPS up to His1, the peptide was cleaved from the resin using cleavage cocktail A. The resulting crude peptide was purified by preparative RP-HPLC using Shiseido Capcell Pak UG80 C18 column [5 μm, 80-Å pore size, 50-mm inner diameter (I.D.) × 250 mm] at 60°C with a gradient of 20 to 60% CH_3_CN with 0.1% TFA over 30 min to obtain peptide segment **1** (313.4 mg, 18%) as a white fluffy lyophilized powder. Opr-[Cys(Acm)49,67]-IL-4(38–74)-photoprotected Leu–α-ketoacid **2** was synthesized on Rink Aaide ChemMatrix loaded with Fmoc-photoprotected Leu–α-ketoacid precursor (2.0 g, 0.24 mmol/g, 0.48 mmol). Pseudoproline Fmoc-Thr(tBu)-Thr(Ψ(Me,Me)pro)-OH and Fmoc-Ala-Ser(Ψ(Me,Me)pro)-OH were used at Thr42-Thr43 and Ala51-Ser52 (2 hours × 2), and double couplings were applied for coupling of Fmoc-Cys(Acm)-OH at Cys49. After Fmoc-SPPS, the peptide was cleaved from the resin using cleavage cocktail A. The resulting crude peptide was purified by preparative RP-HPLC using Shiseido Capcell Pak UG80 C18 column (5 μm, 80-Å pore size, 50-mm I.D. × 250 mm) at 60°C with a gradient of 20 to 50% CH_3_CN with 0.1% TFA over 30 min to obtain peptide segment **2** (476 mg, 21%) as a white fluffy lyophilized powder. Opr-[Cys(Acm)87,94]-IL-4(77–120)-OH **3** was synthesized on HMPB ChemMatrix resin loaded with Fmoc-Ser(tBu)-OH followed by Fmoc-Tyr(tBu)-OH (225 mg, 0.31 mmol/g, 70 mmol). Pseudoproline Fmoc-Ser-Thr(Ψ(Me,Me)pro)-OH was used at Ser101-Thr102 and double coupling was applied for coupling of BocOpr at the end. After Fmoc-SPPS, the peptide was cleaved from the resin using cleavage cocktail B. The resulting crude peptide was purified by preparative RP-HPLC using Shiseido Capcell Pak UG80 C18 column (5 μm, 80-Å pore size, 50-mm I.D. × 250 mm) at 60°C with a gradient of 20 to 70% CH_3_CN with 0.1% TFA over 40 min to obtain peptide segment **3** (12.2 mg, 3.3%) as a white fluffy lyophilized powder.

#### Ligations

Segment **1** (113 mg, 27.4 mmol, 1.3 equiv) and segment **2** (100 mg, 21.1 mmol, 1.0 equiv) were dissolved in 9:1 DMSO:H_2_O with 0.1 M oxalic acid (1.05 ml, 20 mM). After stirring for 15 hours at 60°C, the mixture was cooled to room temperature, diluted with 1:1 CH_3_CN:H_2_O with 0.1% TFA (7.0 mL, 3 mM), and irradiated at a wavelength of 365 nm for 45 min at room temperature. This reaction mixture was purified by preparative RP-HPLC using Shiseido Capcell Pak UG80 C18 column (50 mm by 250 mm) at 60°C with a gradient of 20 to 60% CH_3_CN with 0.1% TFA over 28 min to obtain peptide **4** (91.7 mg, 51%) as a fluffy lyophilized powder. Polypeptide **4** (25.0 mg, 2.91 mmol, 1.0 equiv) and segment **3** (20.1 mg, 3.78 mmol, 1.3 equiv) were dissolved in 9:1 DMSO:H_2_O with 0.1 M oxalic acid (146 ml, 20 mM). After stirring for 24 hours at 60°C, the resulting gel-like mixture was dissolved with DMSO (400 ml) and then added a buffer containing 50 mM sodium carbonate and 6 M Gn·HCl pH 9.5 (1.5 mL, 2 mM). After incubating for 2.5 hours, the mixture was acidified by adding 50% aq. AcOH and purified by preparative RP-HPLC using Shiseido Capcell Pak MGII C18 column (5 μm, 120-Å pore size, 20-mm I.D. × 250 mm) at 60°C with a gradient of 20 to 70% CH_3_CN with 0.1% TFA over 28 min to obtain **5** (13.4 mg, 33% over two steps as a white fluffy lyophilized powder.

#### Acm deprotection

Polypeptide **5** (13.4 mg, 965 nmol) was dissolved in 50% aq. AcOH and AgOAc (32.0 mg, 191 mmol, 200 equiv) was added. After shaking at 50°C for 2 hours in the dark, the mixture was cooled to room temperature and dithiothreitol (32.0 mg, 207 mmol, 215 equiv) was added. The formed precipitate was separated after centrifugation, and the precipitate was washed twice with 50% aq. AcOH. The combined supernatant was purified by preparative RP-HPLC using Shiseido Capcell Pak MGII C18 column (5 μm, 120-Å pore size, 20-mm I.D. × 250 mm) at 60°C with a gradient of 30 to 85% CH_3_CN with 0.1% TFA over 28 min to obtain **6** (10.2 mg, 79%) as a fluffy lyophilized powder.

#### Folding

Polypeptide **6** (1.0 mg, 74.3 nmol, 1.0 equiv) was dissolved in solubilizing buffer 2.0 ml (0.5 mg/ml) containing 4 M Gn·HCl, 50 mM tris, and 5 mM EDTA (pH 8.5). This solution was diluted sixfold with 4 ml of folding buffer containing 0.5 M Arg·HCl, 50 mM tris, and 2 mM Cys (pH 8.5) and incubated 18 hours at room temperature. The mixture was acidified with 50% aq. AcOH and purified by preparative RP-HPLC using Shiseido Proteonavi column (5 μm, 300-Å pore size, 10-mm I.D. × 250 mm) at room temperature with a gradient of 20 to 75% CH_3_CN with 0.1% TFA over 28 min to obtain folded wild-type IL-4 **7** (483 mg, 48%, calculated based on the UV absorbance at 280 nm in H_2_O).

#### Statistical analyses

Mice were randomly assigned to experimental groups and results are shown as means ± SD. Statistical analyses were performed in GraphPad Prism 10. The details of performed statistical tests are provided in the figure legends. *P* values < 0.05 were considered significant.

## Supplementary Materials

### This PDF file includes:

Figs. S1 to S49 Supplementary Text NMR spectra References
