# Supplementary material for: In vitro and in vivo evaluation of chemically synthesized, receptor-biased interleukin-4 and photocaged variants
Source: Sci Adv. 2025 Jun 25;11(26):eadw9755. doi: 10.1126/sciadv.adw9755 (PMC12190007; doi:10.1126/sciadv.adw9755)
Supplement: Supplementary file 1 — Figs. S1 to S49 Supplementary Text NMR spectra References [file sciadv.adw9755_sm.pdf]

Science Advances
AAAS

Supplementary Materials for

In vitro and in vivo evaluation of chemically synthesized, receptor-biased interleukin-4 and photocaged variants

Mamiko Ninomiya *et al.*

Corresponding author: Jeffrey W. Bode, bode@org.chem.ethz.ch; Onur Boyman, onur.boyman@uzh.ch

Sci. Adv. **11**, eadw9755 (2025)
DOI: 10.1126/sciadv.adw9755

This PDF file includes:

Figs. S1 to S49
Supplementary Text
NMR spectra
References

1. Supplementary figures

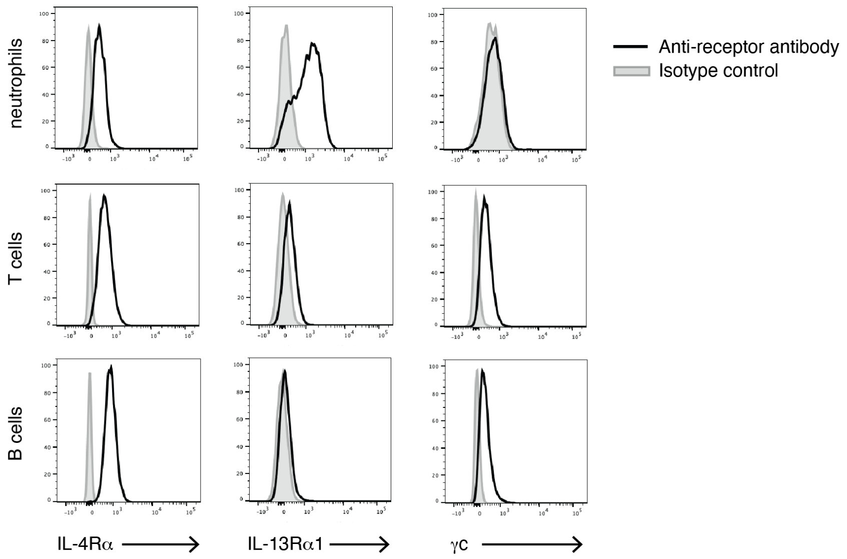

Fig. S1. IL-4 receptor expression varies across neutrophils, T cells, and B cells. Receptor expression of the three IL-4R subunits; IL-4Rα, IL-13Rα1 and γ_c. Histograms showing receptor expression on blood CD3$^-$CD19$^-$ CD11b$^+$Ly6G$^+$ neutrophils (top panel), CD19$^-$CD3$^+$ T cells (middle panel) and CD3$^-$CD19$^+$ B cells (bottom panel).

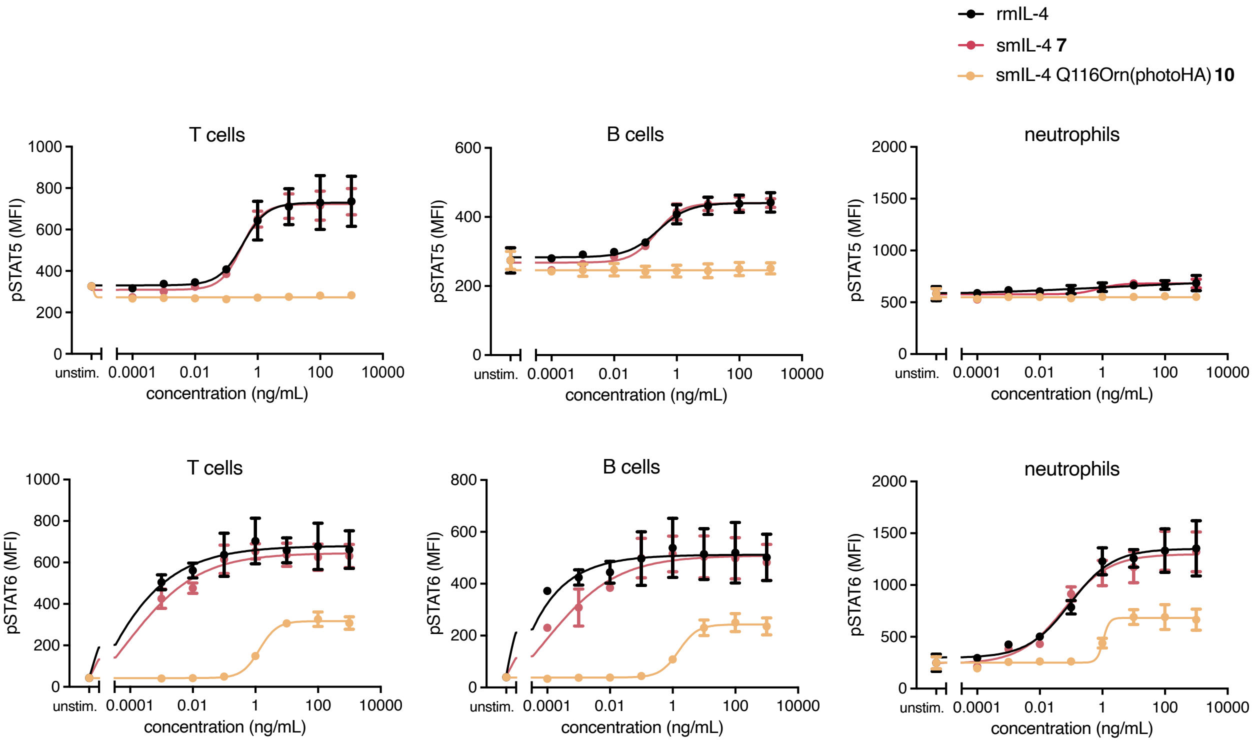

Fig. S2. Synthetic IL-4 variants elicit dose-dependent STAT phosphorylation. Dose-dependent STAT phosphorylation by rIL-4, smIL-4 **7**, and smIL-4 Q116Orn(photoHA) **10** in T cells, B cells, and neutrophils. n = 2.

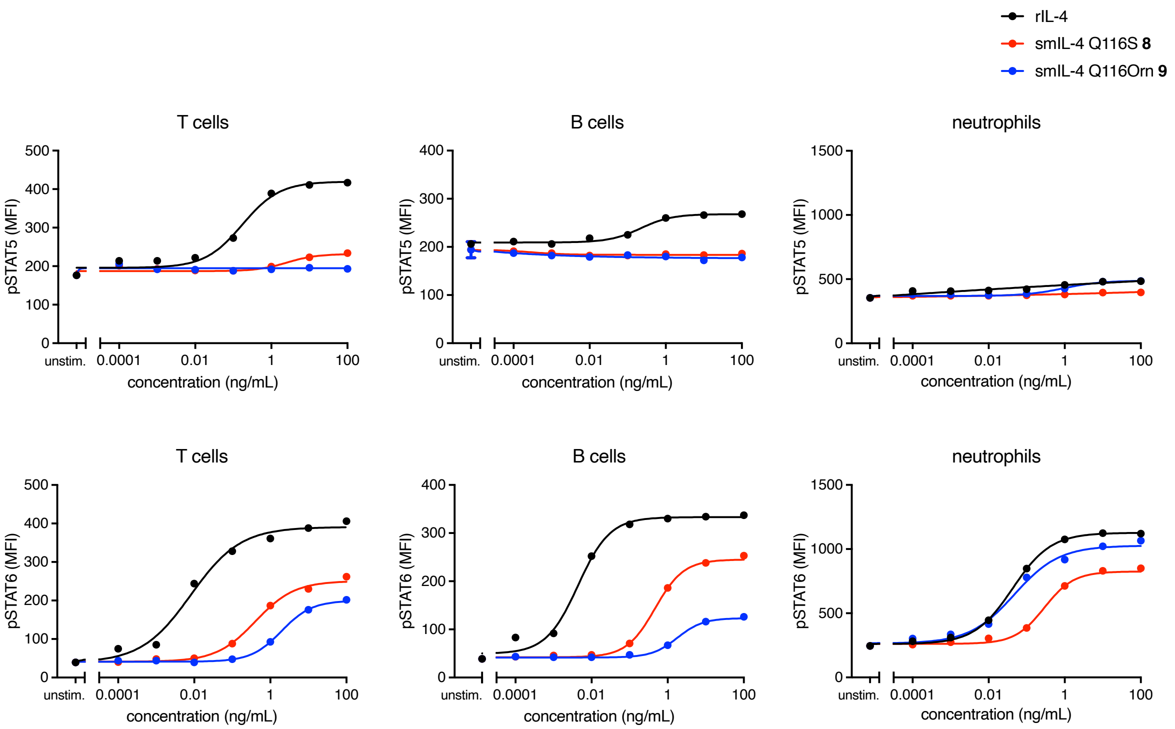

Fig. S3. STAT signaling profiles differ between Q116S and Q116Orn IL-4 variants. Dose-dependent STAT phosphorylation assay by rIL-4, smIL-4 Q116S **8**, and smIL-4 Q116Orn **9** T cells, B cells, and neutrophils. n = 1.

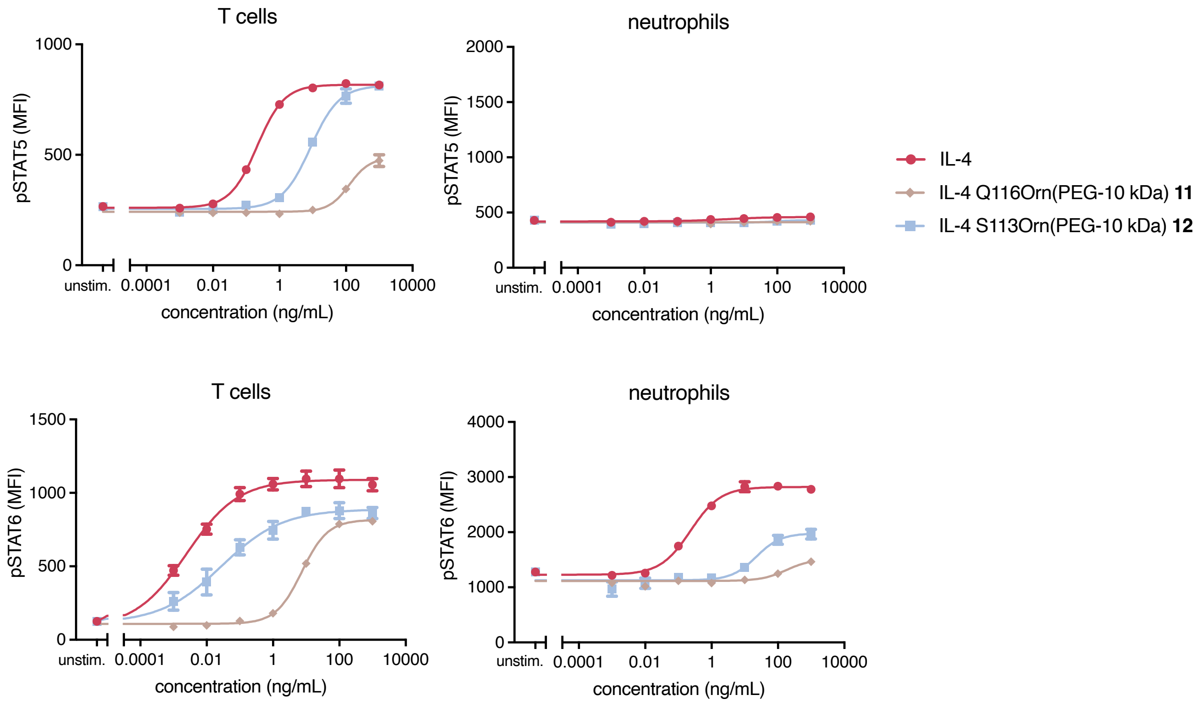

Fig. S4. PEGylated IL-4 variants differently affect STAT signaling. Dose-dependent STAT phosphorylation assay by rIL-4, smIL-4 Q116Orn(PEG-10 kDa) **11**, and smIL-4 S113Orn(PEG-10 kDa) **12** in T cells and neutrophils. n = 2.

a

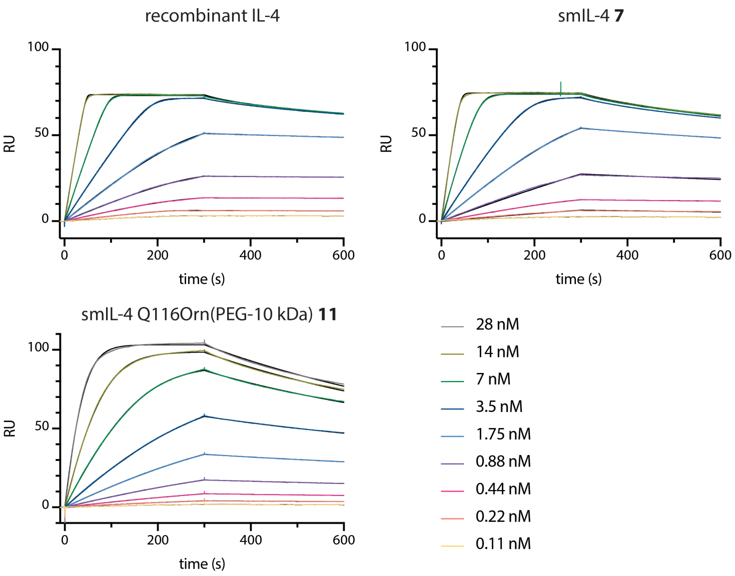

b

	k_{on} (1/Ms)	k_{off} (1/s)	K_D (pM)	RMAX (RU)
rIL-4	1.76 x 10^7	1.20 x 10^{-3}	68.2	74.3
sIL-4 **7**	1.79 x 10^7	1.56 x 10^{-3}	86.9	75.4
**11**	0.156 x 10^7	1.25 x 10^{-3}	804	107

Fig. S5. SPR analysis shows IL-4 variant binding to IL-4Rα. a) SPR diagram of the interaction of IL-4 variants with ectodomain of IL-4Rα which was immobilized on the CMDP sensor chip. The concentrations of perfused IL-4 variants over sensor chip were from top to bottom: 14, 7, 3.5, 1.75, 0.88, 0.44, 0.22, 0.11 nM for recombinant IL-4, synthetic IL-4 **7** and 28, 14, 7, 3.5, 1.75, 0.88, 0.44, 0.22, 0.11 nM for IL-4 Q116Orn(PEG-10 kDa) **11**. b) The table shows the calculated k_{on}, k_{off}, K_D and RMAX values based on SPR experiments with multiple injections.

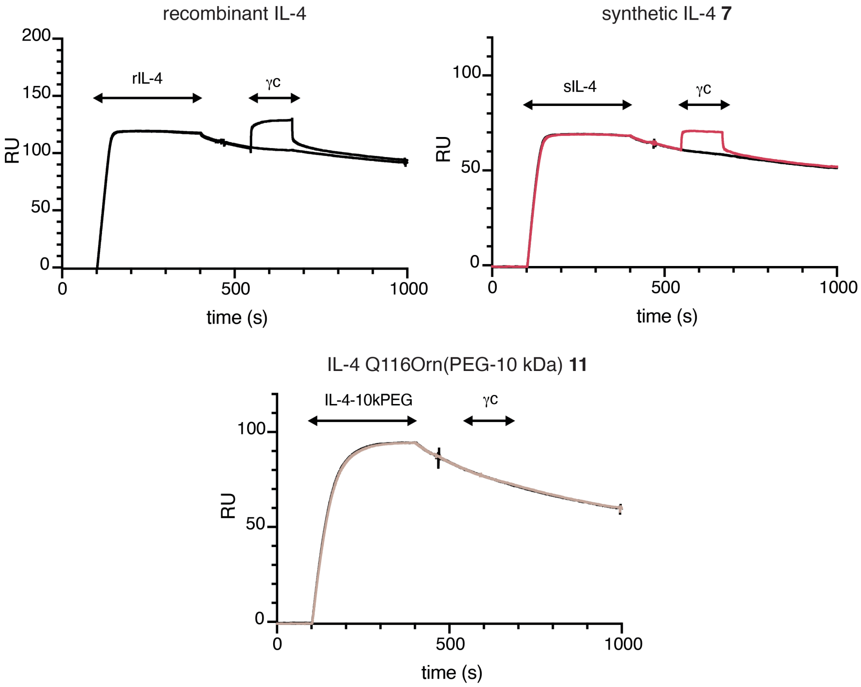

Fig. S6. Secondary receptor binding assessed by SPR. SPR measurement of the interaction of IL-4 bound to IL-4Rα extracellular domain with the γ_c. Solution of γ_c (2 μM) was perfused over a sensor chip with various IL-4 variants bound to immobilized IL-4Rα extracellular domain.

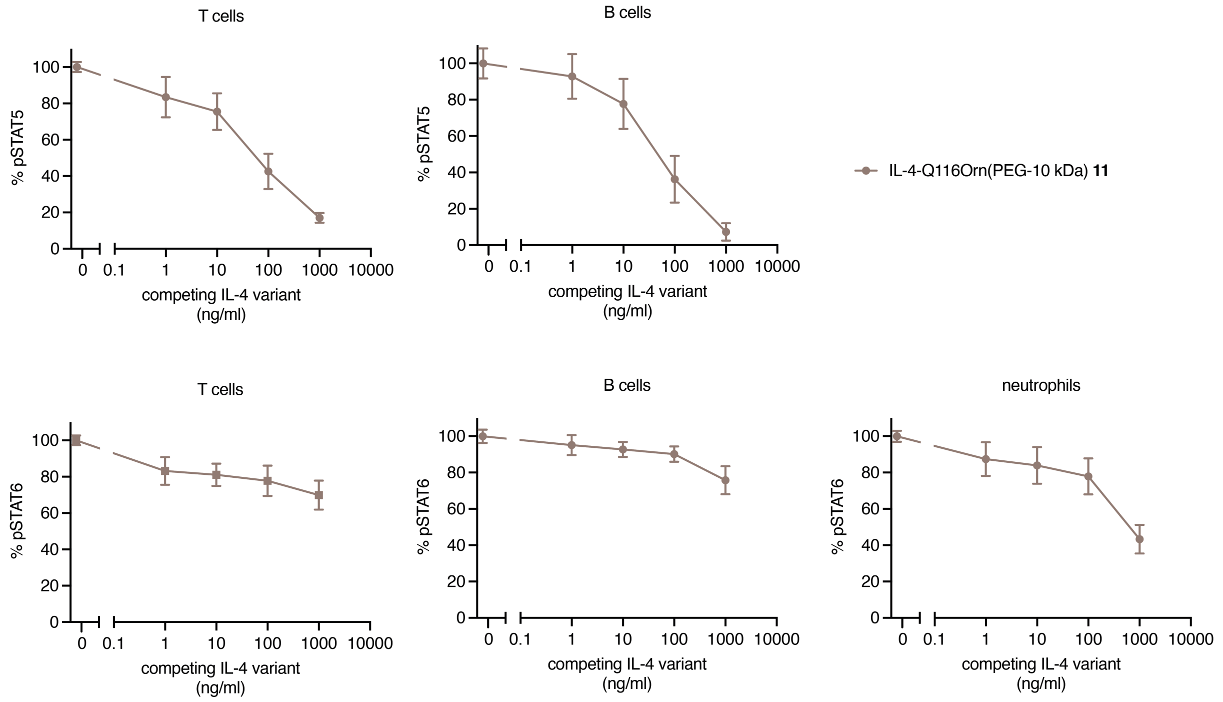

Fig. S7. PEGylated IL-4 variant partially inhibits STAT phosphorylation. Dose-dependent inhibition of STAT5 and STAT6 phosphorylation by smIL-4 Q116Orn(PEG-10 kDa) **11**. Splenocytes were stimulated with 1 ng/mL rIL-4 in the presence of IL-4 variants at the indicated concentrations (based on protein weight). n = 4.

2. Supplementary texts

2.1. General methods

Reagents and solvents

Fmoc-amino acids with suitable side-chain protecting groups, HCTU, and HATU were purchased from Peptides International (Louisville, KY, USA) and ChemImpex (Wood Dale, IL, USA). Dipeptide building blocks and non-standard amino acids were either purchased from PeptArt (Läufelfingen, Switzerland) or Bachem (Bubendorf, Switzerland) or Iris Biotech GMBH. HPLC grade CH_3CN from Sigma-Aldrich was used for analytical and preparative HPLC purification. DMF (>99.8%) from Sigma-Aldrich was directly used for solid phase peptide synthesis without further purification. Fmoc-protected-Leu-α-ketoacid, Fmoc-photoprotected-Leu-α-ketoacid, Fmoc-protected-Val-α-ketoacid, Boc-(*S*)-5-oxaproline, Fmoc-(*S*)-5-oxaproline were prepared according to reported procedures. Solvents for flash column chromatography (hexanes, EtOAc) were of technical grade. Other commercially available reagents and solvents were purchased from Sigma-Aldrich (Buchs, Switzerland), Acros Organics (Geel, Belgium), ABCR (Karlsruhe, Germany) and TCI Europe (Zwijndrecht, Belgium) and used as received.

Characterization

^{1}H- and ^{13}C-NMR spectra were recorded on Bruker DRX400, Bruker AVIII400 and Bruker AVIII600 spectrometers in the solvent indicated. Chemical shifts (δ) for ^{1}H-NMR (400 and 600 MHz) and ^{13}C-NMR (101 and 150 MHz) are expressed in parts per million (ppm) and are referred to residual undeuterated solvent signals. Coupling constants (*J*) *are* reported in Hertz (Hz) and the corresponding splitting patterns are indicated as follows: s, singlet; bs, broad singlet; d, doublet; dd, doublet of doublet; ddd, doublet of doublet of doublet; td, triplet of doublet; t, triplet; m, multiplet; br, broad. Infrared (IR) spectra were recorded on a JASCO FT-IR-4100 spectrometer. High-resolution mass spectra (HRMS) were recorded by the Mass Service of the Laboratory of Organic Chemistry at ETH Zurich either with a Bruker maXis instrument (ESI-MS measurements) equipped with an ESI source and a Qq-TOF detector or with a Bruker solariX instrument (MALDI-FTICR-MS) using 4-hydroxy-α-cyanocinnamic acid as matrix. For ESI (+MS) an enhanced quadratic calibration mode was used with the following reference mass peaks: 118.0863, 322.0481, 622.0290, 922.0098, 1221.9906, 1521.9715, 1821.9523, 2121.9332, 2421.9140, 2721.8948. CD spectrum was measured with a JASCO J-715 spectropolarimeter.

Reactions and purifications

All reactions utilizing air- or moisture-sensitive reagents were performed using standard techniques under an atmosphere of N_2. Flash chromatography was performed on Silicycle SiO_2 Type F60 (230-400 mesh) using a forced flow of air. Reactions and fractions from flash chromatography were monitored by thin layer chromatography (TLC) using precoated glass plates (Merck, silica 60 F254) and visualized by staining with $KMnO_4$ or phosphomolybdic acid.

High-Performance Liquid Chromatography (HPLC)

Peptides and proteins were analyzed and purified by reversed phase high performance liquid chromatography (RP-HPLC) on Jasco analytical and preparative instruments equipped with JASCO LC-Net II/ADC for control unit, PU-2080 or PU-2087 for analytical and preparative HPLC pumps, respectively, MX-2080 for mixing, DG-2080 for in-line degassing, UV-2077 for UV detector (monitoring at 220 nm, 254 nm and 301 nm) and a Rheodyne injector fitted with a 20 or 1000 µl injection loop or on a Gilson preparative instrument fitted with a 20 mL injection loop. If required, the columns were heated using an Alltech column heater or a water bath (preparative HPLC). The mobile phase for RP-HPLC were Milipore-H_2O containing 0.1 % (v/v) TFA and HPLC grade CH_3CN containing 0.1 % (v/v) TFA. Analytical HPLC was performed on a Shiseido Capcell Pak C18 UG120 (5 µm, 120 Å pore size, 4.6 mm I.D. x 250 mm) column, on a Shiseido Capcell Pak C18 UG80 (5 µm, 80 Å pore size, 4.6 mm I.D. x 250 mm) column or on a Shiseido Capcell Pak C18 MGII column (5 µm, 120 Å pore size, 4.6 mm I.D. x 250 mm) columns or Shiseido Capcell Pak C18 MGIII column (5 µm, 120 Å pore size, 4.6 mm I.D. x 250 mm) columns or on a Shiseido Proteonavi column (5 µm, 300 Å pore size 4.6 mm I.D. x 250 mm) at a flow rate of 1 mL/min. Preparative HPLC was performed on a Shiseido Capcell Pak C18 MGII column (5 µm, 120 Å pore size, 10 mm I.D. x 250 mm) or on a Shiseido Proteonavi column (5 µm, 300 Å pore size, 10 mm I.D. x 250 mm) at flow rate 5 mL/min or on a Shiseido Capcell Pak C18 MGII column (5 µm, 120 Å pore size, 20 mm I.D. x 250 mm) or Osaka Soda Capcell Pak C18 MGIII column (5 µm, 120 Å pore size, 20 mm I.D. x 250 mm), or on a Shiseido Proteonavi column (5 µm, 300 Å pore size, 20 mm I.D. x 250 mm) at flow rate 10 mL/min or on a Shiseido Capcell Pak C18 UG80 columns (5 µm, 80 Å pore size, 50 mm I.D. x 250 mm) or on a ReproSil-Pur 120 ODS-3 (5 µm, 120 Å pore size, 50 mm I.D. x 250 mm) at flow rate 40 mL/min.

The following methods were used: For analytical HPLC, the column was pre-equilibrated at the starting solvent composition (e.g. 20% CH_3CN) for 10 min. After injection of the sample, the solvent composition was held at the same solvent composition for 3 min (analytical, 5 min for flow rate 5 or 10 mL/min, 8 min for flow rate 40 mL/min) then run to the final solvent composition (e.g. 70% CH_3CN) over the time indicated. After the gradient run time, the solvent composition was changed to 95% CH_3CN within 1 min and the column was flushed for 5 min (analytical, 5 min for preparative). Within 1 min, the solvent

composition was changed to the starting solvent composition and the run ended. For the sake of simplicity, only the gradient time and the starting and end composition of the eluent will be stated at the individual experiments, although all experiments included the full cycle as described above. Fractions containing the desired product were pooled and lyophilized.

Preloading of 2-chloro trityl resin

2-Chloro trityl resin (1.60 mmol/g) was swollen in CH_2Cl_2 for 10 min and washed with CH_2Cl_2, DMF and CH_2Cl_2. To the resin was added a solution of the corresponding C-terminal Fmoc-Xaa-OH (1.0 equiv relative to desired loading) and *i*Pr_2NEt (2.0 equiv) in CH_2Cl_2 (final concentration ca. 0.2 M) then agitated by bubbling N_2 for 3 h. The resin was washed with CH_2Cl_2 (x5), DMF (x5) and CH_2Cl_2 (x5), and dried. After the estimation of amino acid loading, the remaining 2-chloro trityl was capped by treating with a solution of *i*Pr_2NEt/CH_2Cl_2/MeOH (1:3:1 v/v/v) for 20 min. The resin was washed with CH_2Cl_2 (x5), DMF (x5) and CH_2Cl_2 (x5).

Preloading of HMPB ChemMatrix® resin

HMPB ChemMatrix® resin (0.44 mmol/g) was swollen in DMF and solutions of the corresponding C-terminal Fmoc-Xaa-OH (4.0 equiv), 1-(mesitylene-2-sulfolnyl)-3-nitro-1H-1,2,4-triazole (MSNT) (4.0 equiv), and *N*-methyl imidazole (8.0 equiv) in CH_2Cl_2 (final concentration ca. 0.2 M) were added, followed by agitations by bubbling N_2 for 4 h. The resin was washed with CH_2Cl_2 (x 5), DMF (x 5) and CH_2Cl_2 (x 5) and dried. After the estimation of amino acid loading by following protocol, Fmoc deprotection was performed with 20% (v/v) piperidine in DMF (10 min x 2), then resin was washed with DMF (x 5), CH_2Cl_2 (x 5) and DMF (x 5). A solution of the next amino acid Fmoc-Xaa-OH (1.0 equiv relative to the desired loading) and HCTU (0.95 equiv) in DMF was added NMM (2.0 equiv) and incubated for 3 min to pre-activate the acid, then added to the resin. The resin was agitated by bubbling nitrogen for 2 h, washed with DMF (x 5), CH_2Cl_2 (x 5) and DMF (x 5) and dried. After the estimation of amino acid loading by following protocol, unreacted free amine was capped by treatment with 20% (v/v) acetic anhydride and 10% (v/v) NMM in DMF (10 min x 2) and washed with DMF (x 5), CH_2Cl_2 (x 5) and DMF (x 5)

Preloading of Fmoc-protected-α-ketoacid on Rink amide polystyrene resin

Fmoc protected Rink amide polystyrene resin (0.56 mmol/g) was swollen in DMF and Fmoc deprotection was performed with 20% (v/v) piperidine in DMF (10 min x 2), then resin was washed with DMF (x 5), CH_2Cl_2 (x 5) and DMF (x 5). To a solution of Fmoc-protected-Xaa-a-ketoacid monomer (1.0 equiv to the desired loading) and HCTU (0.95 equiv) in DMF was added NMM (2.0 equiv) and incubated for 3 min to pre-activate the acid, then added to the resin. The resin was agitated by bubbling N_2 (g) for 3 h, washed

with DMF (x 5), CH_2Cl_2 (x 5) and DMF (x 5) and dried. After the estimation of the amino acid loading by following the protocol, unreacted free amine was capped by treatment with a mixture of acetic anhydride/NMM/DMF (1:1:4. v/v, 5 min x 2) and washed with DMF (x 5), CH_2Cl_2 (x 5) and DMF (x 5).

Preloading of Fmoc-protected-α-ketoacid on Rink amide ChemMatrix® resin

Rink amide ChemMatrix® resin (0.47 mmol/g) was swollen in DMF. To a solution of Fmoc-protected-Xaa-a-ketoacid monomer (1.0 equiv relative to the desired loading) and HCTU (0.95 equiv) in DMF was added NMM (2.0 equiv) and incubated for 3 min to pre-activate the acid, then added to the resin. The resin was agitated by bubbling N_2 (g) for 3 h, washed with DMF (x 5), CH_2Cl_2 (x 5) and DMF (x 5) and dried. After the estimation of the amino acid loading by the following the protocol, unreacted amines were capped by treatment with 20% acetic anhydride and 10% NMM in DMF (5 min x 2) and washed with DMF (x 5), CH_2Cl_2 (x 5) and DMF (x 5).

Estimation of amino acid loading by UV spectrophotometry

To the dried pre-loaded resin (10–20 mg) was added a solution of 2 mL of 2% (v/v) DBU in DMF. After shaking for 15 min, 48 μL of the solution was taken and added to CH_3CN (2952 μL) and UV absorbance (A) was measured at 304 nm with a UV spectrophotometer. As a blank, 48 μL of 2% (v/v) DBU in DMF without resin was added to CH_3CN 2952 μL and the UV absorbance was measured.
Loading is calculated with the equation as follows:[54] Loading (mmol/g) = (A x 16.4)/ mass of resin (mg)

Surface Plasmon Resonance

Surface Plasmon Resonance (SPR) experiments were conducted on a Biacore T100 or S100 instrument. Protein concentrations were quantified by BCA assay or UV absorbance at 280 nm. The kinetic data was analyzed using the Biacore evaluation software with a 1:1 Langmuir binding model. Experiments used a Xantec CMDP sensor chip. Ectodomain of mIL-4Rα as a Fc chimera (Sino biologics) or ectodomain of hIL-4Rα as a His tag fusion was covalently immobilized to the sensor chip surface by EDC/NHS protocol. The buffer in all experiments was 10 mM HEPES, 0.15 M NaCl, 3 mM EDTA and 0.05% v/v tween 20 at pH 7.3. Kinetic runs were performed at flow rate of 50 μL/min at 20 °C and various proteins at different concentrations were flowed over sensor chip for 300 s for association and consecutively running buffer was flowed 300 s for dissociation. The chip surface was regenerated by a flow of a buffer 0.1 M NaOAc, 1 M NaCl, pH 3.0 for 30 s at a flow rate of 5 μL/min. For the second binding receptor affinity measurements (Figure S3), the surface was saturated first saturated with IL-4 variants (30 nM) for 300 s, followed by a flow of γc (1 μM) for 120 s.

2.2. Synthesis of mouse interleukin-4 7

Synthesis of H_2N-[Cys(Acm)^{5,27}]-IL-4(1–35)-Leu-α-ketoacid 1

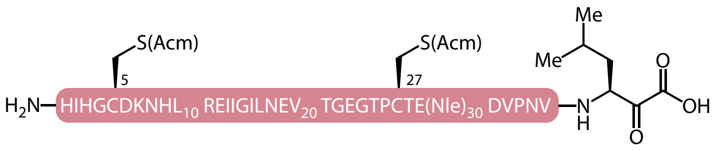

HRMS (MALDI-FTMS) *m/z* calcd for $C_{176}H_{285}N_{51}O_{58}S_2$ $[M+H]^+$ 4108.0486, found *m/z* 4108.0388.

a) b)

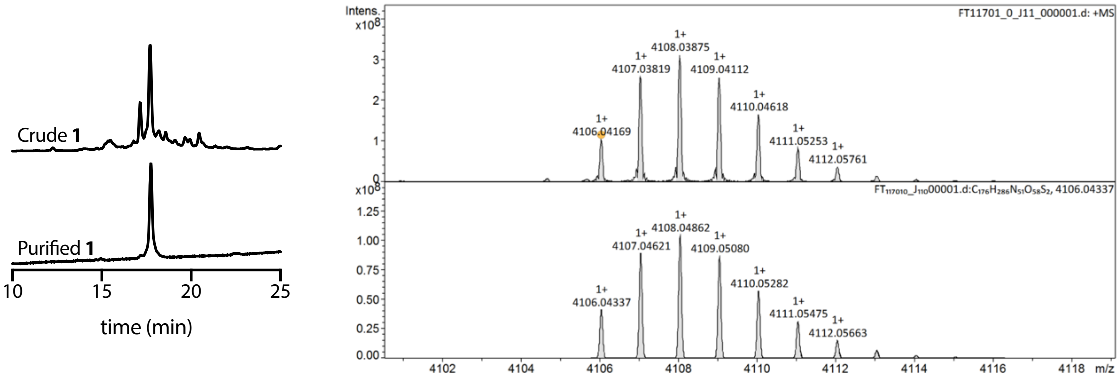

Fig. S8. Analytical data of 1. a) Analytical HPLC trace of the crude and purified **1** (20 to 95% CH_3CN with 0.1% TFA over 14 min on a Shiseido Capcell Pak C18 UG120, λ = 220 nm) b) HRMS (MALDI-FTMS) of **1**. Measured (top) and calculated (bottom).

Synthesis of Opr-[Cys(Acm)^{49,67}]-IL-4(38–74)-photoprotected-Leu-α-ketoacid 2

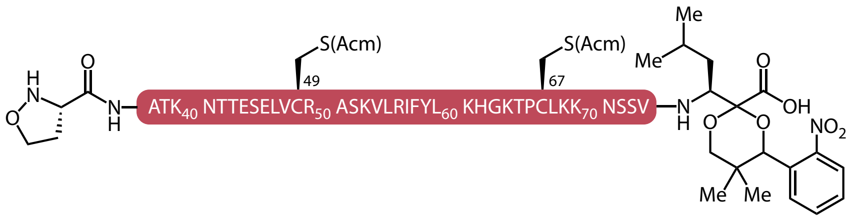

HRMS (MALDI-FTMS) calcd for $C_{210}H_{348}N_{58}O_{62}S_2$ ($[M+H]^+$) *m/z* 4741.5429, found *m/z* 4741.5381.

a) b)

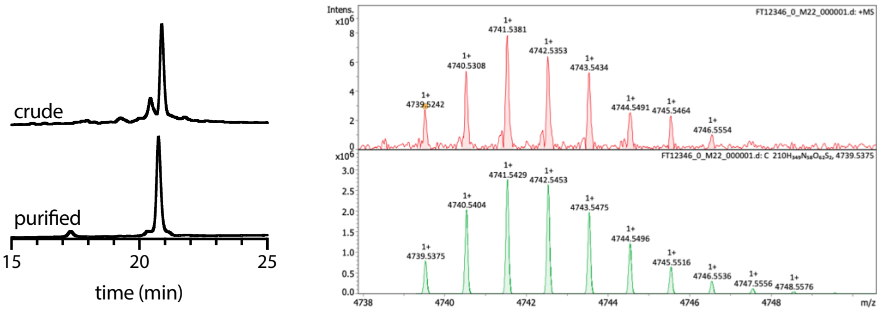

Fig. S9. Analytical data of 2. a) Analytical HPLC trace of the crude and purified **2**. (25 to 50% CH_3CN with 0.1% TFA over 14 min on a Shiseido Capcell Pak C18 UG120, λ = 220 nm) b) HRMS (ESI) of **2**.

Synthesis of Opr-[Cys(Acm)^{87,94}]-IL-4(77–120)-OH 3

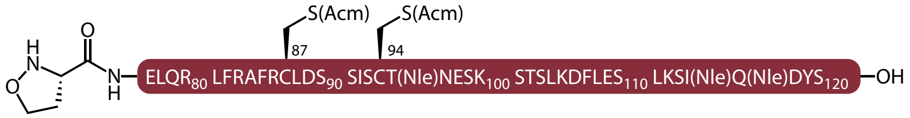

HRMS (ESI) *m/z* calcd for $C_{233}H_{378}N_{62}O_{76}S_2$ $[M+3H]^{3+}$ 1777.0101, $[M+4H]^{4+}$ 1333.0094, $[M+5H]^{5+}$ 1066.6090, [M] 5327.7140 , found *m/z* 1776.9157, 1332.9411, 1066.5554, 5327.7348.

a) b)

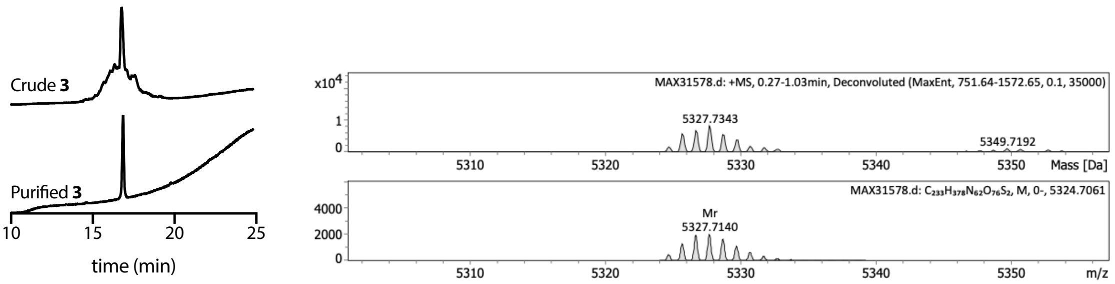

Fig. S10. Analytical data of 3. a) Analytical HPLC trace of the crude and purified **3** (20 to 95% CH_3CN with 0.1% TFA over 14 min on a Shiseido Capcell Pak C18 UG120, λ = 220 nm). b) HRMS (ESI) of **3**. Deconvoluted (top) and calculated (bottom).

Synthesis of H_2N-[Cys(Acm)^{5,27,49,67}]-IL-4(1-74)-Leu-α-ketoacid containing isopeptide bond at Leu^{36}-Hse^{37} 4

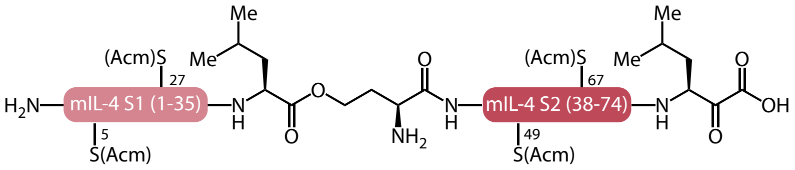

HRMS (ESI) *m/z* calcd for $C_{374}H_{620}N_{108}O_{115}S_4$ $[M+4H]^{4+}$ 2150.4731, $[M+5H]^{5+}$ 1720.5799, $[M+6H]^{6+}$ 1433.9845, $[M+7H]^{7+}$ 1229.2735, [M] 8597.4996, found *m/z* 2150.3843, 1720.5111, 1433.9282, 1229.2240, 1075.5713, 8597.5225.

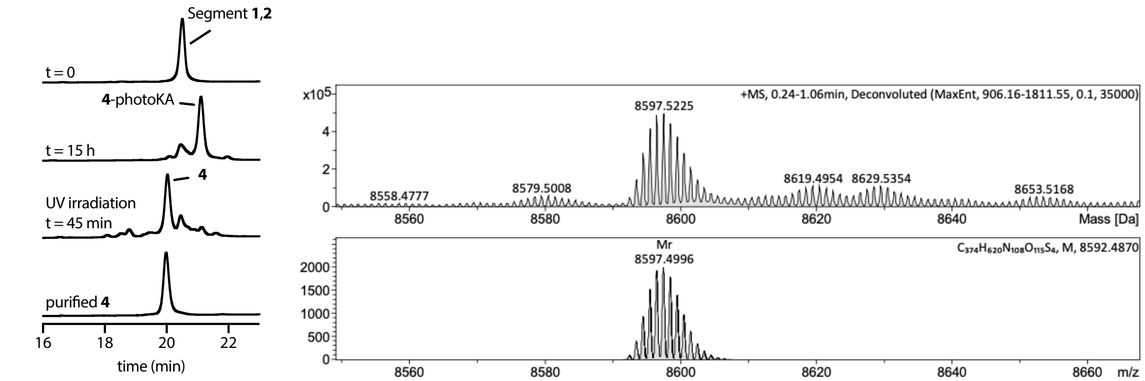

Fig. S11. Analytical data of 4. a) Analytical HPLC trace of the KAHA ligation over time (20 to 60% CH_3CN with 0.1% TFA over 14 min on a Shiseido Capcell Pak C18 UG120, λ = 220 nm). b. HRMS (ESI) of **4**. Deconvoluted (top) and calculated (bottom).

Synthesis of H_2N-[Cys(Acm)^{5,27,49,67,87,94}]-IL-4(1-120)-OH 5

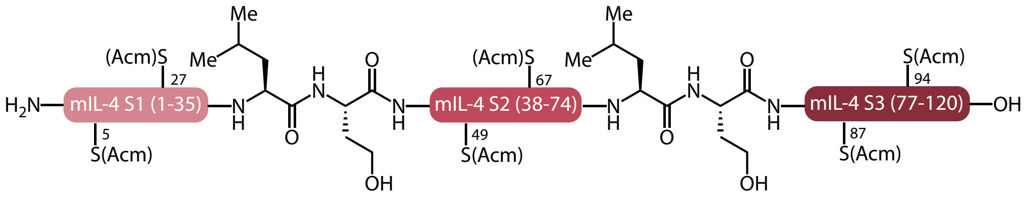

HRMS (ESI) *m/z* calcd for $C_{606}H_{998}N_{170}O_{189}S_6$ $[M+8H]^{8+}$ 1736.2400, $[M+9H]^{9+}$ 1543.4364, $[M+10H]^{10+}$ 1389.1935, $[M+11H]^{11+}$ 1262.9948, $[M+12H]^{12+}$ 1157.8292, [M] 13881.2235, found *m/z* 1736.2879, 1543.3676, 1389.1325, 1262.9385, 1157.8606, 13881.2411.

a) b)

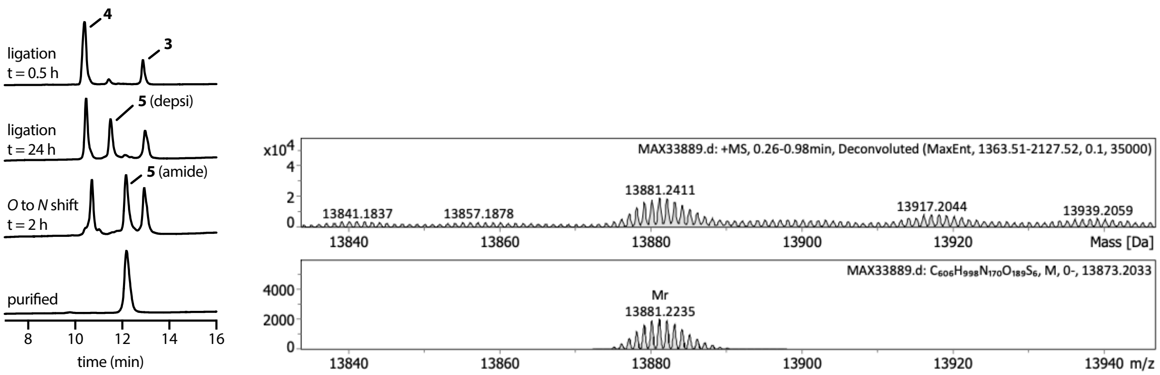

Fig. S12. Analytical data of 5. a) Analytical HPLC trace of the KAHA ligation and O to N acyl shift (20 to 95% CH_3CN with 0.1% TFA over 14 min on a Shiseido Capcell Pak UG80 C18 UG120, λ = 220 nm). b) Deconvoluted HRMS (ESI) spectrum of **5**.

Synthesis of H_2N-IL-4(1-120)-OH 6

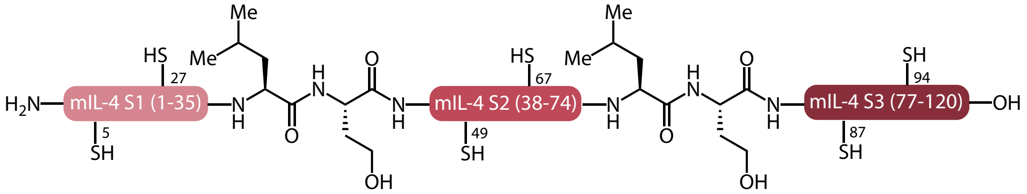

HRMS (ESI) *m/z* calcd for $C_{588}H_{968}N_{164}O_{183}S_6$ $[M+6H]^{6+}$ 2243.5729, $[M+7H]^{7+}$ 1923.2064, $[M+8H]^{8+}$ 1682.9315, $[M+9H]^{9+}$ 1496.0510, $[M+10H]^{10+}$ 1346.5467, $[M+11H]^{11+}$ 1224.2249, [M] 13455.0003, found *m/z* 2243.5099, 1923.0113, 1682.8871, 1496.0118, 1346.5118, 1224.1938, 13454.0463.

a) b)

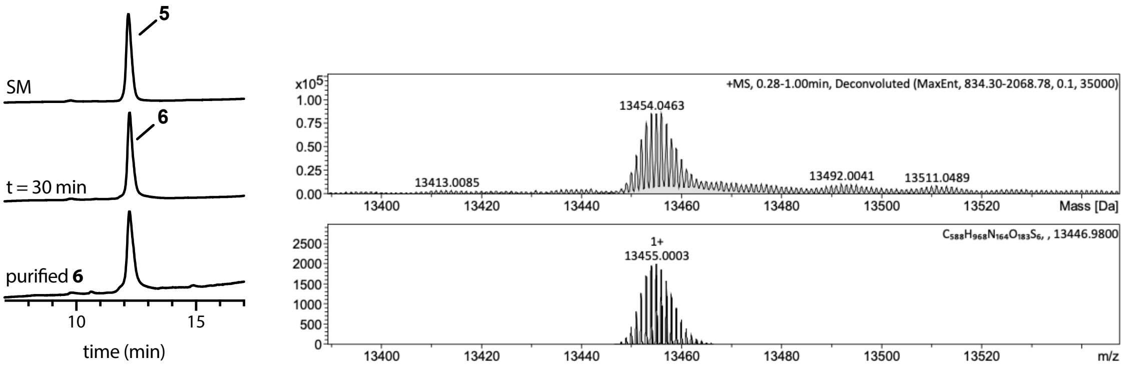

Fig. S13. Analytical data of 6. a) Analytical HPLC trace of the Acm deprotection (20 to 95% CH_3CN with 0.1% TFA over 14 min on a Shiseido Capcell Pak UG80 C18 UG120, λ = 220 nm). b) HRMS (ESI) of **6**. Deconvoluted (top), calculated (bottom).

Synthesis of folded H-IL-4(1-120)-OH 7

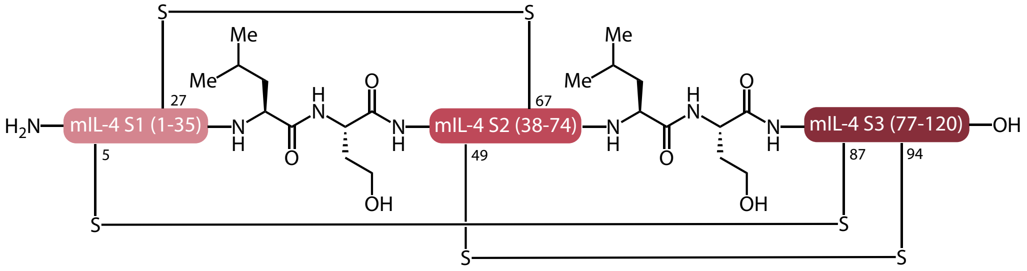

HRMS (ESI) *m/z* calcd for $C_{588}H_{962}N_{164}O_{183}S_6$ $[M+6H]^{6+}$ 2242.5649, $[M+7H]^{7+}$ 1922.3424, $[M+8H]^{8+}$ 1682.1755, [M] 13448.9539, found *m/z* 2242.4989, 1922.2881, 1682.1294, 13448.9630.

a) b)

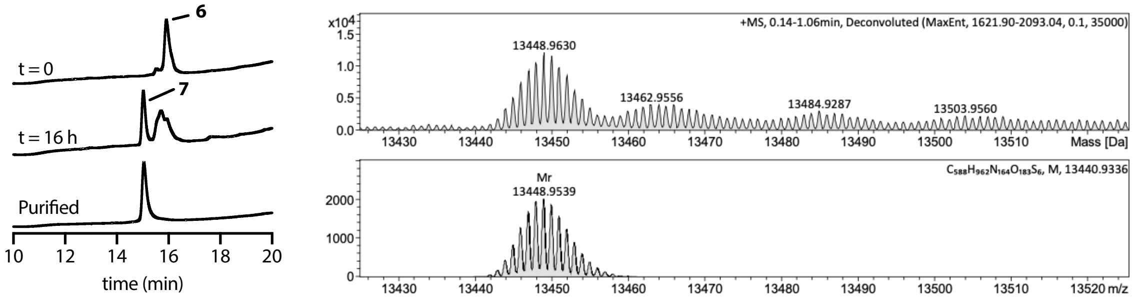

Fig. S14. Analytical data of 7. a) Analytical HPLC of the folding (20 to 95% CH_3CN with 0.1% TFA over 14 min on a Shiseido Capcell Pak UG80 C18, λ = 220 nm). b) HRMS (ESI) of **7**. Deconvoluted (top), calculated (bottom).

Synthesized folded IL-4 **7** was dissolved in 10 mM potassium phosphate buffer (pH 6.8) and the CD spectrum was measured at 20 °C using a 0.1 cm cell. Protein concentration was determined by the absorbance at 280 nm.

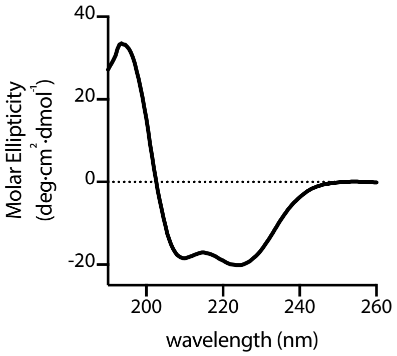

Fig. S15. CD spectrum of 7.

2.3. Synthesis of mouse IL-4 Q116S 8

Synthesis of Opr-[Cys(Acm)^{87,94}, Ser^{116}]-IL-4(77–120)-OH

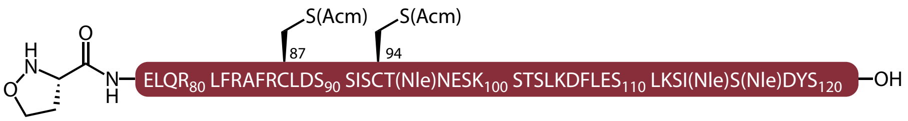

Opr-[Cys(Acm)^{87,94}, Ser^{116}]-IL-4(77–120)-OH **S1** was synthesized on HMPB ChemMatrix resin loaded with Fmoc-Ser(tBu)-OH followed by Fmoc-Tyr(tBu)-OH (130 mg, 0.271 mmol/g, 35 µmol). Pseudoprolines Fmoc-Ile-Ser(Ψ(Me,Me)pro)-OH and Fmoc-Ser-Thr(Ψ(Me,Me)pro)-OH were used at Ile92-Ser93 and Ser101-Thr102. Double coupling was applied for coupling of Fmoc-Ser-Thr(Ψ(Me,Me)pro)-OH. After Fmoc-SPPS, the peptide was cleaved from the resin using cleavage cocktail B. The resulting crude peptide was purified by preparative RP-HPLC using Shiseido Capcell Pak UG80 C18 column (50 x 250 mm) at 60 °C with a gradient of 20 to 70% CH_3CN with 0.1% TFA over 40 min to obtain peptide segment **S1** (9.4 mg, 5%) as a white fluffy lyophilized powder.

HRMS (ESI) *m/z* calcd for $C_{231}H_{375}N_{61}O_{76}S_2$ $[M+3H]^{3+}$ 1763.3261, $[M+4H]^{4+}$ 1322.7464, $[M+5H]^{5+}$ 1058.3986, $[M+6H]^{6+}$ 882.1667, [M] 5283.6796, found *m/z* 1763.2347, 1322.4267, 1058.3428, 882.1201, 5286.6757.

a) b)

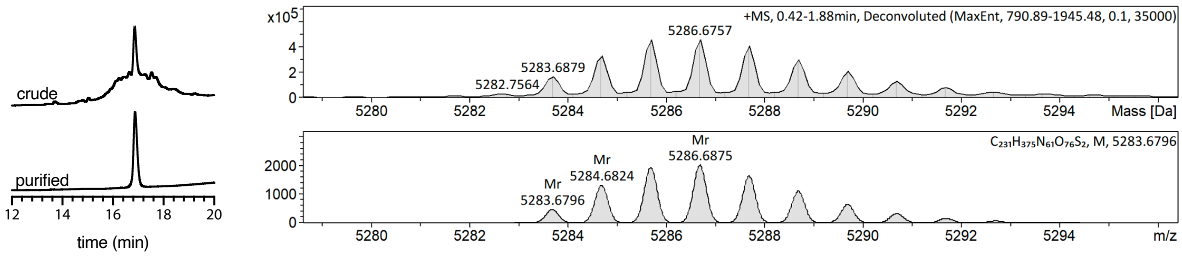

Fig. S16. Analytical data of S1. a) Analytical HPLC trace of the crude and purified **S1** (20 to 95% CH_3CN with 0.1% TFA over 14 min on a Shiseido Capcell Pak C18 UG120, λ = 220 nm). b) HRMS (ESI) of **S1**. Deconvoluted (top) and calculated (bottom).

Synthesis of H_2N-[Cys(Acm)^{5,27,49,67,87,94}, Ser^{116}]-IL-4(1-120)-OH containing isopeptide bond at Leu^{36}-Hse^{37} and Leu^{75}-Hse^{76} S2

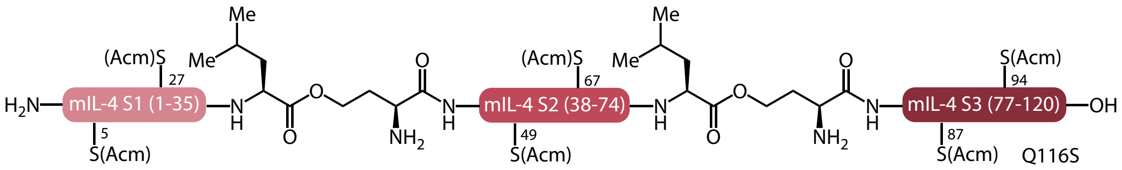

Polypeptide **4** (11.7 mg, 1.37 µmol, 1.0 equiv) and segment **S1** (9.4 mg, 1.78 µmol, 1.3 equiv) were dissolved in 9:1 DMSO:H_2O with 0.1 M oxalic acid (137 µL, 10 mM). After stirring for 23 h at 60 °C, the resulting gel-like mixture was dissolved with DMSO (1 mL) and purified by preparative RP-HPLC using Shiseido Capcell Pak MGII C18 column (5 µm, 120 Å pore size, 20 mm I.D. x 250 mm) at 60 °C with a gradient of 20 to 75% CH_3CN with 0.1% TFA over 28 min to obtain **S2** (6.5 mg, 34%) as a white fluffy lyophilized powder.

HRMS (ESI) *m/z* calcd for $C_{604}H_{995}N_{169}O_{189}S_6$ $[M+11H]^{11+}$ 1259.2628, $[M+12H]^{12+}$ 1154.4082, $[M+13H]^{13+}$ 1065.6850, $[M+14H]^{14+}$ 989.6366, $[M+15H]^{15+}$ 923.7280, [M] 13840.1971, found *m/z* 1259.2065, 1154.3553, 1065.5610, 989.5914, 923.6845, 13840.1841.

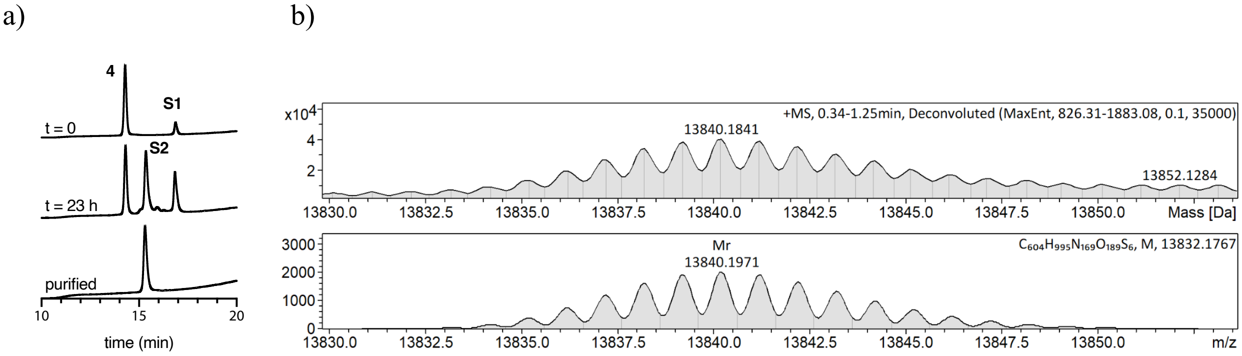

Fig. S17. Analytical data of S2. a) Analytical HPLC trace of the KAHA ligation (20 to 95% CH_3CN with 0.1% TFA over 14 min on a Shiseido Capcell Pak C18 UG120, λ = 220 nm). b) HRMS (ESI) of **S2**. Deconvoluted (top) and calculated (bottom).

Synthesis of H_2N-[Cys(Acm)^{5,27,49,67,87,94}, Ser^{116}]-IL-4(1-120)-OH S3

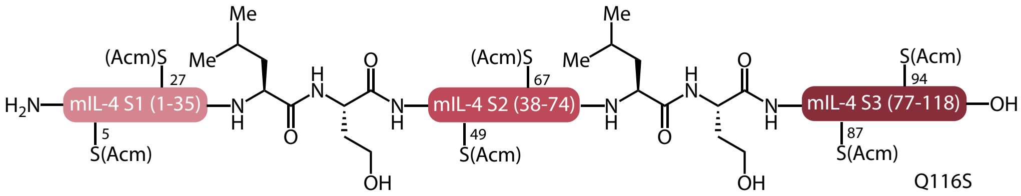

Peptide **S2** (6.5 mg, 0.47 µmol) was dissolved in a buffer containing 50 mM CHES and 6 M Gn·HCl pH 9.5 (2.3 mL, 0.2 mM). After incubating for 2.5 h at room temperature, the mixture was acidified by adding 50% aq. AcOH and purified by preparative RP-HPLC using Shiseido Capcell Pak MGII C18 column (5 µm, 120 Å pore size, 20 mm I.D. x 250 mm) at 60 °C with a gradient of 20 to 75% CH_3CN with 0.1% TFA over 28 min to obtain **S3** (5.3 mg, 82%) as a white fluffy lyophilized powder.

HRMS (ESI) *m/z* calcd for $C_{604}H_{995}N_{169}O_{189}S_6$ $[M+11H]^{11+}$ 1259.2628, $[M+12H]^{12+}$ 1154.4082, $[M+13H]^{13+}$ 1065.6850, $[M+14H]^{14+}$ 989.6366, $[M+15H]^{15+}$ 923.7280, [M] 13840.1971, found *m/z* 1259.2040, 1154.4360, 1065.6327, 989.5884, 923.6815, 13840.1709.

a) b)

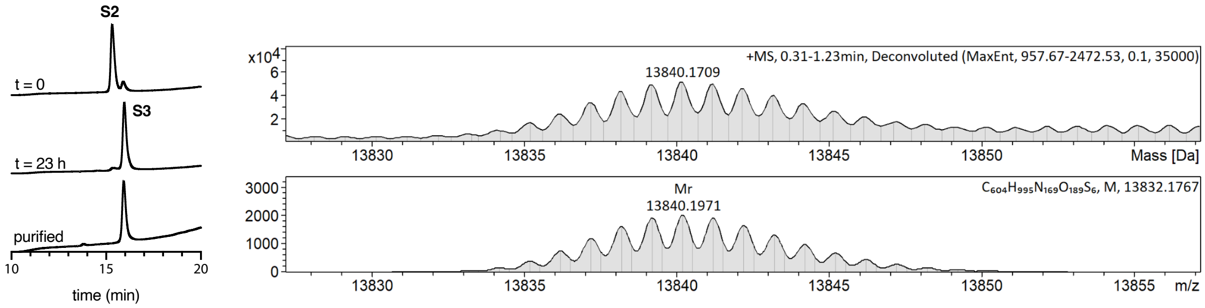

Fig. S18. Analytical data of S3. a) Analytical HPLC trace of the O to N acyl shift of **S2** to **S3** (20 to 95% CH_3CN with 0.1% TFA over 14 min on a Shiseido Capcell Pak C18 UG120, λ = 220 nm). b) HRMS (ESI) of **S3**. Deconvoluted (top) and calculated (bottom).

Synthesis of H_2N-[Ser^{116}]-IL-4(1-120)-OH S4

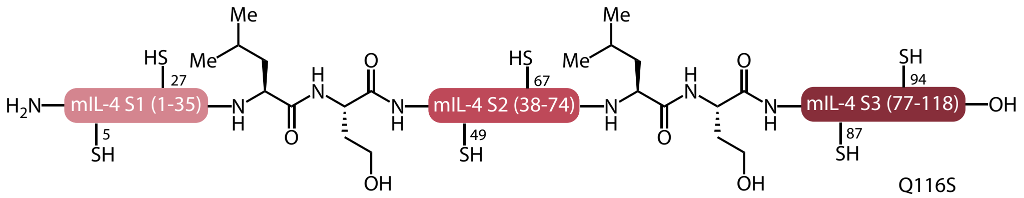

Polypeptide **S3** (5.3 mg, 383 nmol) was dissolved in 50% aq. AcOH (1.3 mL, 0.3 mM) and added AgOAc (13.0 mg, 77.9 µmol). After shaking at 50 °C for 1 h in the dark, the mixture was cooled to room temperature and added DTT (16.0 mg, 104 µmol). The formed precipitate was separated after centrifugation and the precipitate was washed twice with 50% aq. AcOH. The combined supernatant was purified by preparative RP-HPLC using Shiseido Capcell Pak MGII C18 column (5 µm, 120 Å pore size, 20 mm I.D. x 250 mm) at 60 °C with a gradient of 20 to 75% CH_3CN with 0.1% TFA over 28 min to obtain **S4** (3.4 mg, 66 %) as a white fluffy lyophilized powder.

HRMS (ESI) *m/z* calcd for $C_{586}H_{965}N_{163}O_{183}S_6$ $[M+10H]^{10+}$ 1342.4415, $[M+11H]^{11+}$ 1220.4929, $[M+12H]^{12+}$ 1118.8691, $[M+13H]^{13+}$ 1032.8798, [M] 13405.9540, found *m/z* 1342.4027, 1220.4580, 1118.7519, 1032.8481, 13413.9525.

a) b)

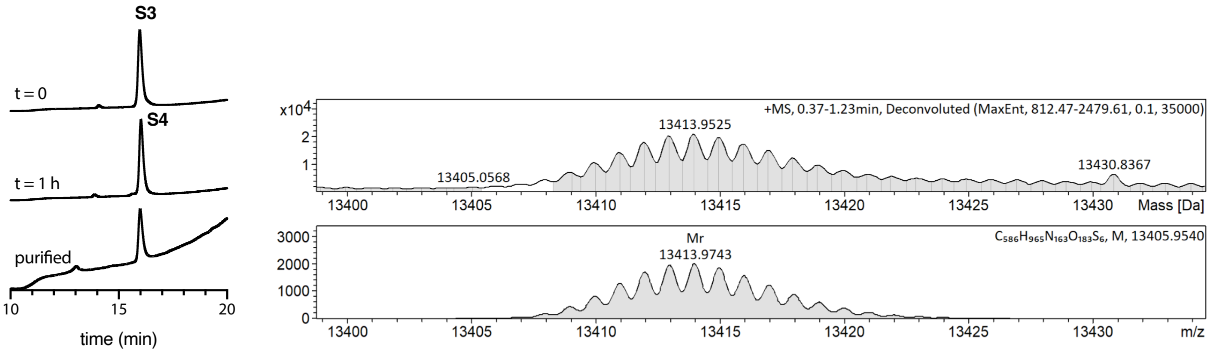

Fig. S19. Analytical data of S4. a) Analytical HPLC trace of the reaction (20 to 95% CH_3CN with 0.1% TFA over 14 min on a Shiseido Capcell Pak C18 UG120, λ = 220 nm). b) HRMS (ESI) of **S4**. Deconvoluted (top), calculated (bottom).

Synthesis of folded H-[Ser^{116}]-IL-4(1-120)-OH 8

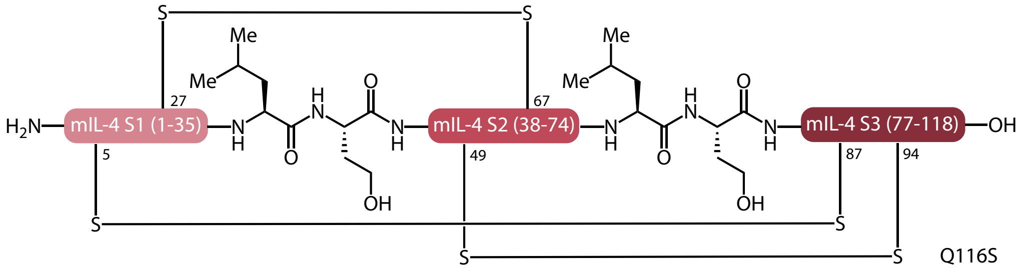

Polypeptide **S4** (3.4 mg, 0.25 µmol) was dissolved in solubilizing buffer 6.8 mL (0.5 mg/mL) containing 4 M Gn·HCl, 50 mM tris and 5 mM EDTA (pH 8.5). This solution was then diluted with 34 mL of folding buffer containing 0.5 M Arg·HCl, 50 mM Tris, 2 mM Cys (pH 8.5) and incubated 18 h at room temperature. The mixture was acidified with 6 mL of 50% aq. AcOH and purified by preparative RP-HPLC using Shiseido Proteonavi column (5 µm, 300 Å pore size, 20 mm I.D. x 250 mm) at room temperature with a gradient of 20 to 75% CH_3CN with 0.1% TFA over 28 min to obtain folded IL-4 variant **8** (1.1 mg, 32%, calculated based on BCA assay) as a white fluffy lyophilized powder.

HRMS (ESI) *m/z* calcd for $C_{586}H_{959}N_{163}O_{183}S_6$ [M] 13407.9273, found *m/z* 13407.9087.

a) b)

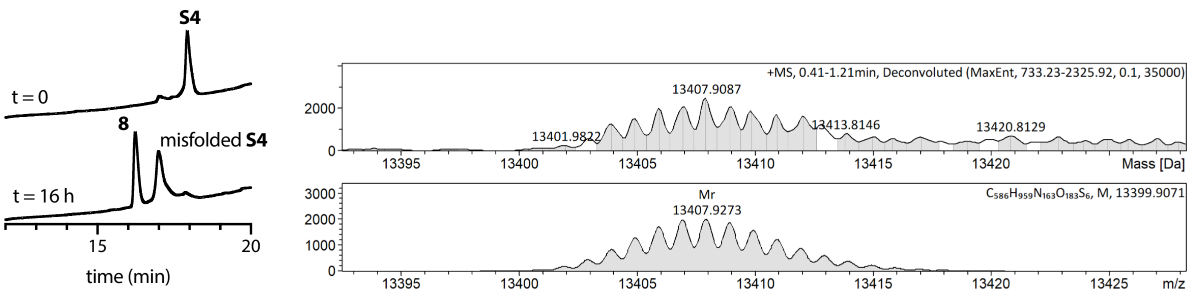

Fig. S20. Analytical data of 8. a) Analytical HPLC of the folding (20 to 80% CH_3CN with 0.1% TFA over 14 min on a Shiseido proteonavi, λ = 220 nm). b) HRMS (ESI) of **8**. Deconvoluted (top), calculated (bottom).

2.4. Synthesis of mouse IL-4 Q116Orn 9

Synthesis of Opr-[Cys(Acm)^{87,94}, Orn^{116}]-IL-4(77–120)-OH S5

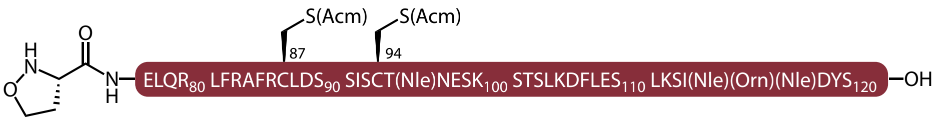

Opr-[Cys(Acm)^{87,94}, Orn^{116}]-IL-4(77–120)-OH **S5** was synthesized on HMPB ChemMatrix resin loaded with Fmoc-Ser(tBu)-OH followed by Fmoc-Tyr(tBu)-OH (130 mg, 0.271 mmol/g, 35 µmol). Pseudoprolines Fmoc-Ile-Ser(Ψ(Me,Me)pro)-OH and Fmoc-Ser-Thr(Ψ(Me,Me)pro)-OH were used at Ile92-Ser93 and Ser101-Thr102. Double coupling was applied for coupling of Fmoc-Ser-Thr(Ψ(Me,Me)pro)-OH. After Fmoc-SPPS, the peptide was cleaved from the resin using cleavage cocktail B. The resulting crude peptide was purified by preparative RP-HPLC using Shiseido Capcell Pak UG80 C18 column (50 x 250 mm) at 60 °C with a gradient of 20 to 70% CH_3CN with 0.1% TFA over 40 min to obtain peptide segment **S5** (7.0 mg, 3.8%) as a white fluffy lyophilized powder.

HRMS (ESI) *m/z* calcd for $C_{233}H_{380}N_{62}O_{75}S_2$ $[M+3H]^{3+}$ 1772.3489, $[M+4H]^{4+}$ 1329.5135, $[M+5H]^{5+}$ 1063.8123, $[M+6H]^{6+}$ 886.6782, [M] 5310.7268, found *m/z* 1772.2504, 1329.4395, 1063.7518, 886.6272, 5313.7248.

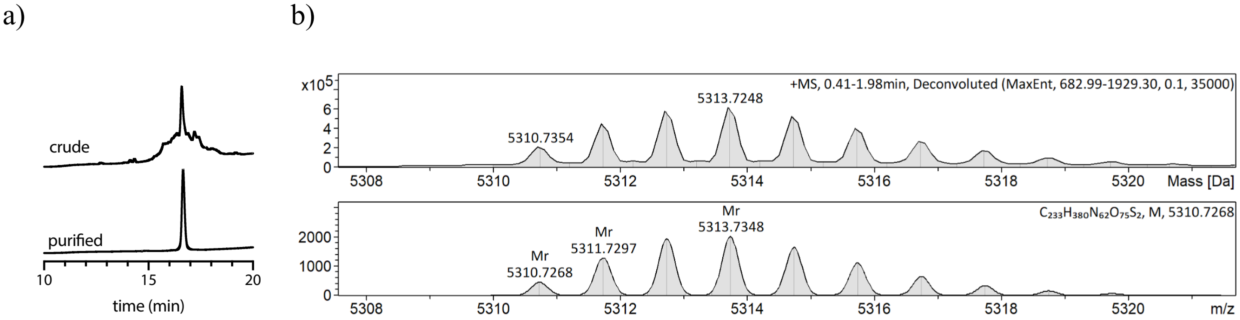

Fig. S21. Analytical data of S5. a) Analytical HPLC trace of the crude and purified **S5** (20 to 95% CH_3CN with 0.1% TFA over 14 min on a Shiseido Capcell Pak C18 UG120, λ = 220 nm). b) HRMS (ESI) of **S5**. Deconvoluted (top) and calculated (bottom).

Synthesis of H_2N-[Cys(Acm)^{5,27,49,67,87,94}, Orn^{116}]-IL-4(1-120)-OH containing isopeptide bond at Leu^{36}-Hse^{37} and Leu^{75}-Hse^{76} S6

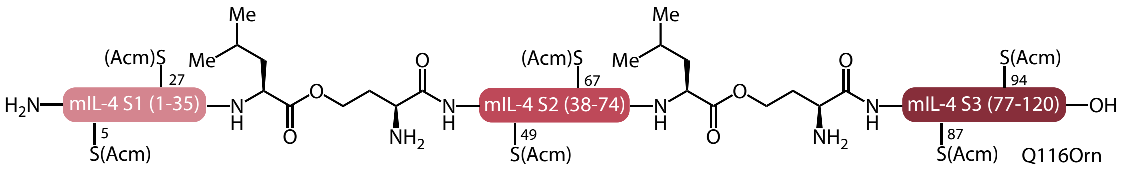

Polypeptide **4** (8.7 mg, 1.01 µmol, 1.0 equiv) and segment **S5** (7.0 mg, 1.32 µmol, 1.3 equiv) were dissolved in 9:1 DMSO:H_2O with 0.1 M oxalic acid (101 µL, 10 mM). After stirring for 23 h at 60 °C, the resulting gel-like mixture was dissolved with DMSO (1 mL) and purified by preparative RP-HPLC using Shiseido Capcell Pak MGII C18 column (5 µm, 120 Å pore size, 20 mm I.D. x 250 mm) at 60 °C with a gradient of 20 to 75% CH_3CN with 0.1% TFA over 28 min to obtain **S6** (6.0 mg, 43%) as a white fluffy lyophilized powder.

HRMS (ESI) *m/z* calcd for $C_{606}H_{1000}N_{170}O_{188}S_6$ $[M+11H]^{11+}$ 1261.7235, $[M+12H]^{12+}$ 1156.6639, $[M+13H]^{13+}$ 1067.7672, $[M+14H]^{14+}$ 991.5701, $[M+15H]^{15+}$ 925.5326, [M] 13867.2443, found *m/z* 1261.9910, 1156.6115, 1067.7178, 991.5228, 925.4871, 13867.2522.

a) b)

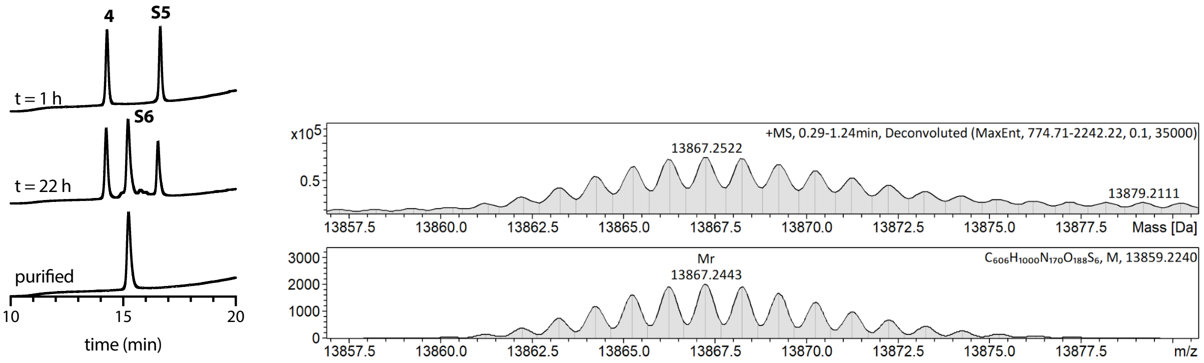

Fig. S22. Analytical data of S6. a) Analytical HPLC trace of the KAHA ligation (20 to 95% CH_3CN with 0.1% TFA over 14 min on a Shiseido Capcell Pak C18 UG120, λ = 220 nm). b) HRMS (ESI) of **S6**. Deconvoluted (top) and calculated (bottom).

Synthesis of H_2N-[Cys(Acm)^{5,27,49,67,87,94}, Orn^{116}]-IL-4(1-120)-OH S7

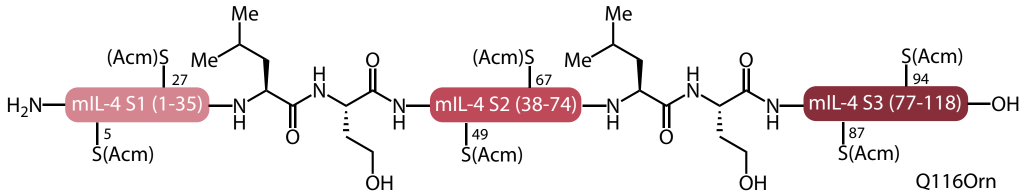

Peptide **S6** (6.0 mg, 0.43 µmol) was dissolved in a buffer containing 50 mM CHES and 6 M Gn·HCl pH 9.5 (2.2 mL, 0.2 mM). After incubating for 2.5 h at room temperature, the mixture was acidified by adding 50% aq. AcOH and purified by preparative RP-HPLC using Shiseido Capcell Pak MGIII C18 column (5 µm, 120 Å pore size, 20 mm I.D. x 250 mm) at 60 °C with a gradient of 20 to 75% CH_3CN with 0.1% TFA over 28 min to obtain **S7** (4.7 mg, 78%) as a white fluffy lyophilized powder.

HRMS (ESI) *m/z* calcd for $C_{606}H_{1000}N_{170}O_{188}S_6$ $[M+11H]^{11+}$ 1261.7235, $[M+12H]^{12+}$ 1156.6639, $[M+13H]^{13+}$ 1067.7672, $[M+14H]^{14+}$ 991.5701, $[M+15H]^{15+}$ 925.5326, [M] 13867.2443, found *m/z* 1261.7532, 1156.6071, 1067.7909, 991.5207, 925.4866, 13867.2106.

a) b)

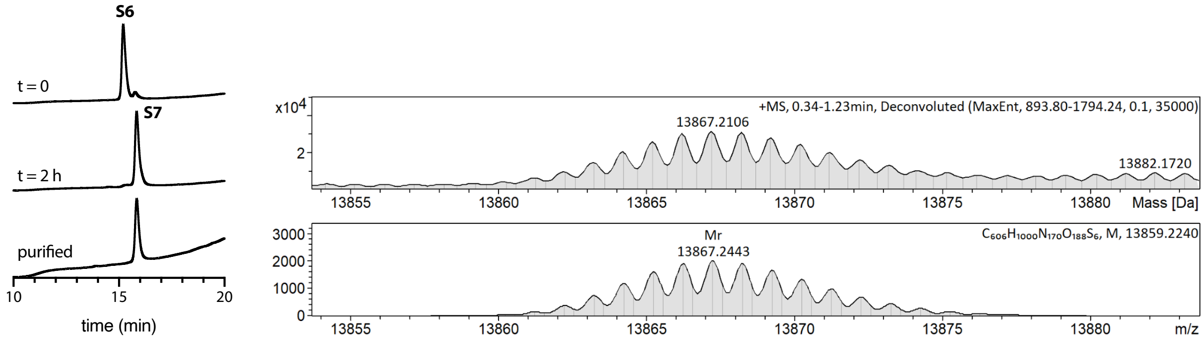

Fig. S23. Analytical data of S7. a) Analytical HPLC trace of the reaction (20 to 95% CH_3CN with 0.1% TFA over 14 min on a Shiseido Capcell Pak C18 UG120, $\lambda = 220$ nm). b) HRMS (ESI) of **S7**. Deconvoluted (top) and calculated (bottom).

Synthesis of H_2N-[Orn^{116}]-IL-4(1-120)-OH S8

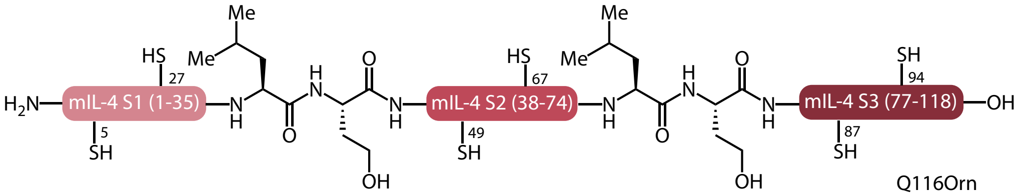

Polypeptide **S7** (4.7 mg, 0.34 µmol) was dissolved in 50% aq. AcOH (1.1 mL, 0.3 mM) and added AgOAc (12.0 mg, 71.9 µmol). After shaking at 50 °C for 1 h in the dark, the mixture was cooled to room temperature and DTT (16.0 mg, 104 µmol) added. The resulting precipitate was separated after centrifugation and the precipitate was washed twice with 50% aq. AcOH. The combined supernatant was purified by preparative RP-HPLC using Shiseido Capcell Pak MGII C18 column (5 µm, 120 Å pore size, 20 mm I.D. x 250 mm) at 60 °C with a gradient of 20 to 75% CH_3CN with 0.1% TFA over 28 min to obtain **S8** (2.9 mg, 64 %) as a white fluffy lyophilized powder.

HRMS (ESI) *m/z* calcd for $C_{588}H_{970}N_{164}O_{182}S_6$ $[M+11H]^{11+}$ 1222.9537, $[M+12H]^{12+}$ 1121.1248, $[M+13H]^{13+}$ 1034.9620, [M] 13441.0216, found *m/z* 1223.0966, 1121.0901, 1034.9298, 13441.0118.

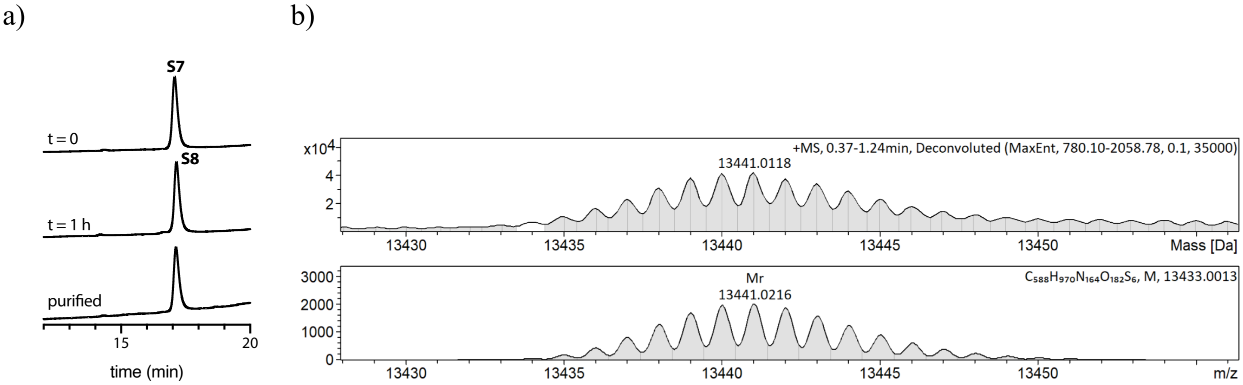

Fig. S24. Analytical data of S8. a) Analytical HPLC trace of the reaction (20 to 95% CH_3CN with 0.1% TFA over 14 min on a Shiseido Capcell Pak C18 UG120, λ = 220 nm). b) HRMS (ESI) of **S8**. Deconvoluted (top), calculated (bottom).

Synthesis of folded H-[Orn^{116}]-IL-4(1-120)-OH 9

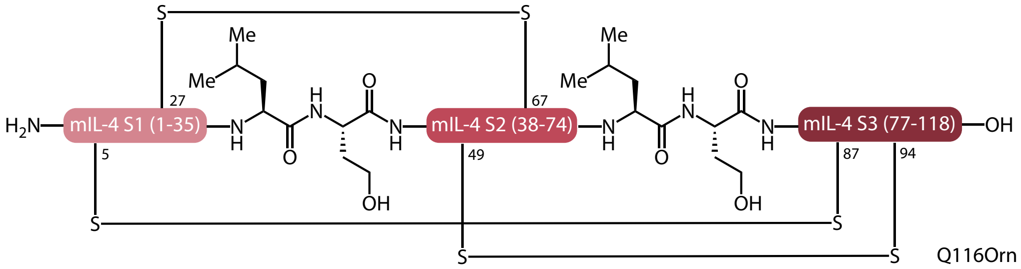

Polypeptide **S8** (2.9 mg, 0.22 µmol) was dissolved in solubilizing buffer 5.8 mL (0.5 mg/mL) containing 4 M Gn·HCl, 50 mM tris and 5 mM EDTA (pH 8.5). This solution was then diluted with 29 mL of folding buffer containing 0.5 M Arg·HCl, 50 mM Tris, 2 mM Cys (pH 8.5) and incubated 18 h at room temperature. The mixture was acidified with 4 mL of 50% aq. AcOH and purified by preparative RP-HPLC using Shiseido Proteonavi column (5 µm, 300 Å pore size, 20 mm I.D. x 250 mm) at room temperature with a gradient of 20 to 75% CH_3CN with 0.1% TFA over 28 min to obtain folded IL-4 variant **9** (1.1 mg, 32%, calculated based on a BCA assay) as a white fluffy lyophilized powder.

HRMS (ESI) *m/z* calcd for $C_{588}H_{964}N_{164}O_{182}S_6$ $[M+7H]^{7+}$ 1920.3448, $[M+8H]^{8+}$ 1680.4276, $[M+9H]^{9+}$ 1493.8254, [M] 13434.9746, found *m/z* 1920.4304, 1680.3774, 1493.7795, 13434.9567.

a) b)

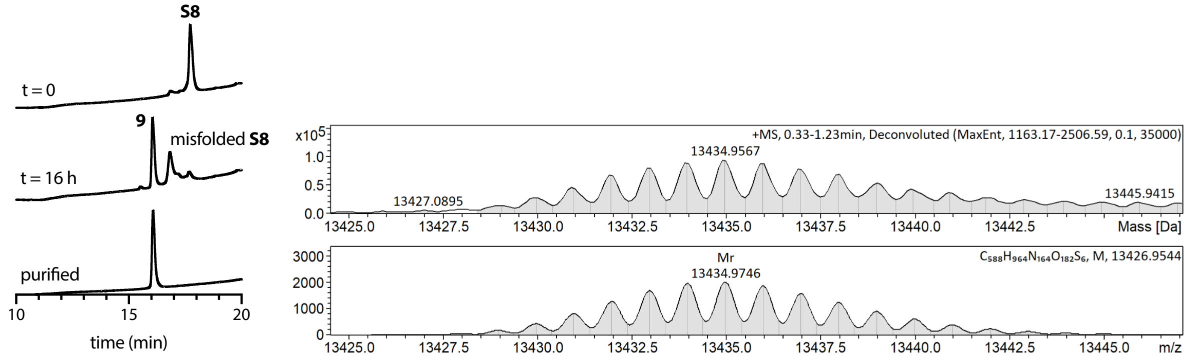

Fig. S25. Analytical data of 9. a) Analytical HPLC of the folding (20 to 80% CH_3CN with 0.1% TFA over 14 min on a Shiseido proteonavi, λ = 220 nm). b) HRMS (ESI) of **9**. Deconvoluted (top), calculated (bottom).

2.5. Synthesis of mouse IL-4 Q116Orn(photoHA) 10

Synthesis of Opr-[Cys(Acm)^{87,94}, Orn(photoHA)^{116}]-IL-4(77–120)-OH S9

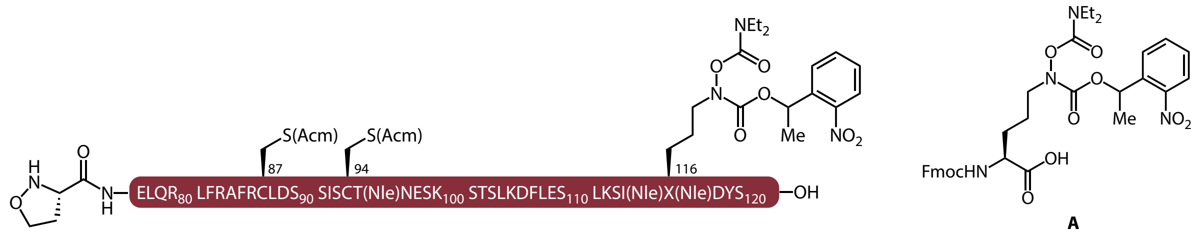

Opr-[Cys(Acm)^{87,94}, Orn-photoHA^{116}]-IL-4(77–120)-OH **S9** was synthesized on HMPB ChemMatrix resin loaded with Fmoc-Ser(tBu)-OH followed by Fmoc-Tyr(tBu)-OH (220 mg, 0.271 mmol/g, 60 µmol). Pseudoprolines Fmoc-Ile-Ser(Ψ(Me,Me)pro)-OH and Fmoc-Ser-Thr(Ψ(Me,Me)pro)-OH were used at Ile92-Ser93 and Ser101-Thr102. Fmoc-Orn(photoHA)-OH **A** was prepared according the reported procedure[36] and was used for the coupling at Gln116. Double coupling was applied for coupling of Fmoc-Ser-Thr(Ψ(Me,Me)pro)-OH. After Fmoc-SPPS, the peptide was cleaved from the resin using cleavage cocktail B. The resulting crude peptide was purified by preparative RP-HPLC using Shiseido Capcell Pak UG80 C18 column (50 x 250 mm) at 60 °C with a gradient of 20 to 70% CH_3CN with 0.1% TFA over 40 min to obtain peptide segment **S9** (30.8 mg, 9.1%) as a white fluffy lyophilized powder.

HRMS (ESI) *m/z* calcd for $C_{247}H_{396}N_{64}O_{81}S_2$ $[M+3H]^{3+}$ 1875.1113, $[M+4H]^{4+}$ 1406.5854, $[M+5H]^{5+}$ 1125.4698, $[M+6H]^{6+}$ 938.0594, [M] 5621.8357, found *m/z* 1875.2860, 1406.4663, 1125.3758, 937.9809, 5621.8464.

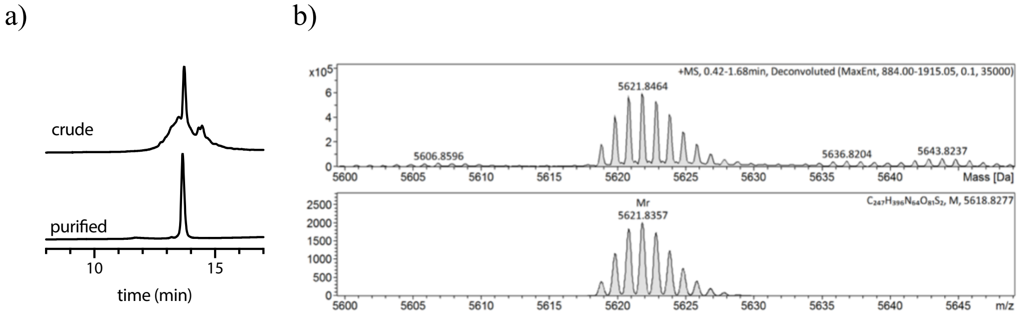

Fig. S26. Analytical data of S9. a) Analytical HPLC trace of the crude and purified **S9** (20 to 95% CH_3CN with 0.1% TFA over 14 min on a Shiseido Capcell Pak C18 UG120, λ = 220 nm). b) HRMS (ESI) of **S9**. Deconvoluted (top) and calculated (bottom).

Synthesis of H_2N-[Cys(Acm)^{5,27,49,67,87,94}, Orn(photoHA)^{116}]-IL-4(1-120)-OH containing isopeptide bond at Leu^{36}-Hse^{37} and Leu^{75}-Hse^{76} S10

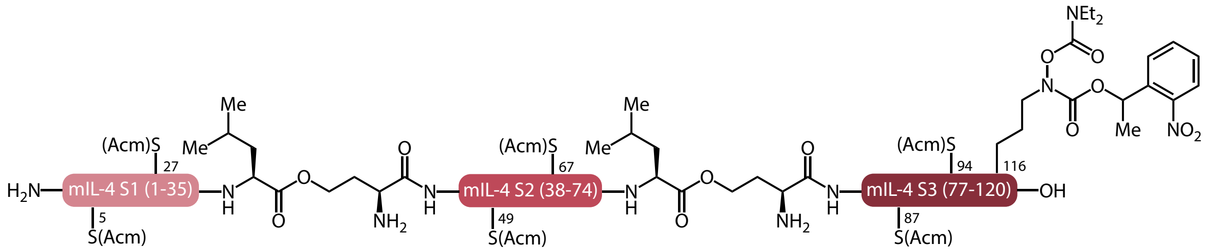

Polypeptide **4** (27.9 mg, 3.24 µmol, 1.0 equiv) and segment **S9** (23.7 mg, 4.22 µmol, 1.3 equiv) were dissolved in 9:1 DMSO:H_2O with 0.1 M oxalic acid (324 µL, 10 mM). After stirring for 23 h at 60 °C, the resulting gel-like mixture was dissolved with DMSO (1 mL) and purified by preparative RP-HPLC using Shiseido Capcell Pak MGII C18 column (5 µm, 120 Å pore size, 20 mm I.D. x 250 mm) at 60 °C with a gradient of 20 to 70% CH_3CN with 0.1% TFA over 28 min to obtain **S10** (15.0 mg, 33%) as a white fluffy lyophilized powder.

HRMS (ESI) *m/z* calcd for $C_{620}H_{1016}N_{172}O_{194}S_6$ $[M+11H]^{11+}$ 1289.7497, $[M+12H]^{12+}$ 1182.3545, $[M+13H]^{13+}$ 1091.4816, $[M+14H]^{14+}$ 1013.5906, $[M+15H]^{15+}$ 946.0851, [M] 14175.3452, found *m/z* 1289.6758, 1282.3692, 1091.4182, 1013.5318, 946.0946, 14175.3571.

a) b)

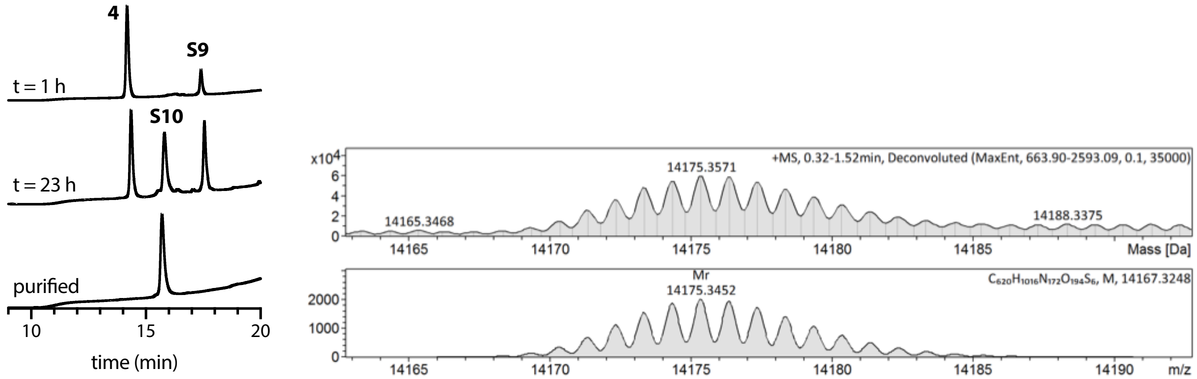

Fig. S27. Analytical data of S10. a) Analytical HPLC trace of the KAHA ligation (20 to 95% CH_3CN with 0.1% TFA over 14 min on a Shiseido Capcell Pak C18 UG120, λ = 220 nm). b) HRMS (ESI) of **S10**. Deconvoluted (top) and calculated (bottom).

Synthesis of H_2N-[Cys(Acm)^{5,27,49,67,87,94}, Orn(photoHA)^{116}]-IL-4(1-120)-OH S11

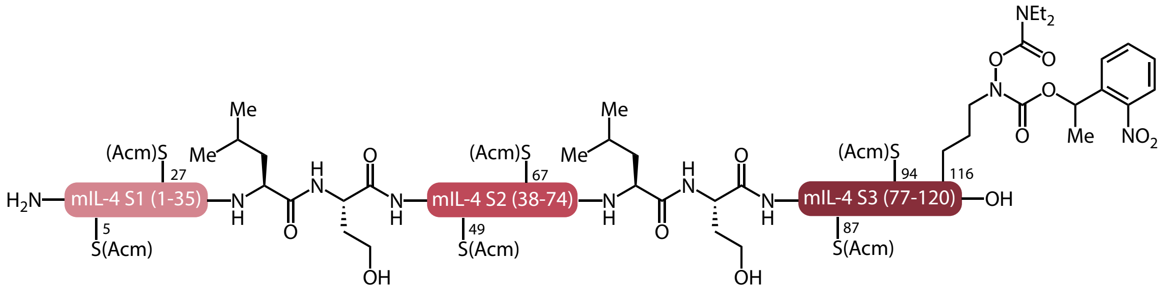

Peptide **S10** (15.0 mg, 1.06 µmol) was dissolved in a buffer containing 50 mM sodium carbonate and 6 M Gn·HCl pH 9.5 (5.3 mL, 0.2 mM). After incubating for 2.5 h at room temperature, the mixture was acidified by adding 50% aq AcOH and purified by preparative RP-HPLC using Shiseido Capcell Pak MGII C18 column (5 µm, 120 Å pore size, 20 mm I.D. x 250 mm) at 60 °C with a gradient of 20 to 70% CH_3CN with 0.1% TFA over 28 min to obtain **S11** (12.2 mg, 81%) as a white fluffy lyophilized powder.

HRMS (ESI) *m/z* calcd for $C_{620}H_{1016}N_{172}O_{194}S_6$ $[M+11H]^{11+}$ 1289.7497, $[M+12H]^{12+}$ 1182.3545, $[M+13H]^{13+}$ 1091.4816, $[M+14H]^{14+}$ 1013.5906, $[M+15H]^{15+}$ 946.0851, [M] 14175.3452, found *m/z* 1289.6734, 1182.3672, 1091.4164, 1013.5293, 946.0932, 14175.3324.

a) b)

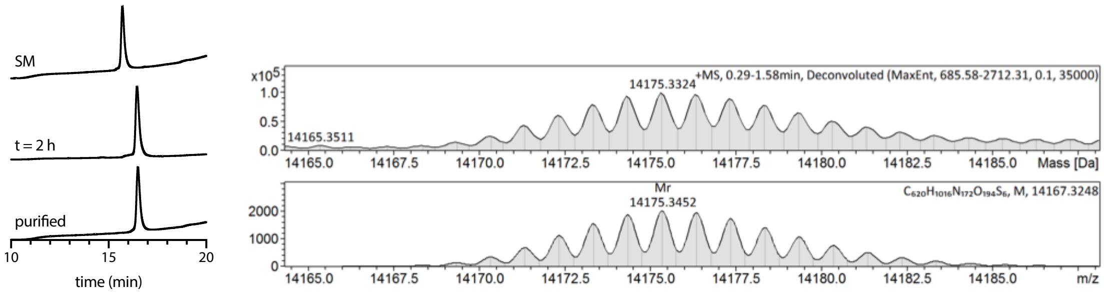

Fig. S28. Analytical data of S11. a) Analytical HPLC trace of the reaction (20 to 95% CH_3CN with 0.1% TFA over 14 min on a Shiseido Capcell Pak C18 UG120, λ = 220 nm). b) HRMS (ESI) of **S11**. Deconvoluted (top) and calculated (bottom).

Synthesis of H_2N-[Orn(photoHA)^{116}]-IL-4(1-120)-OH S12

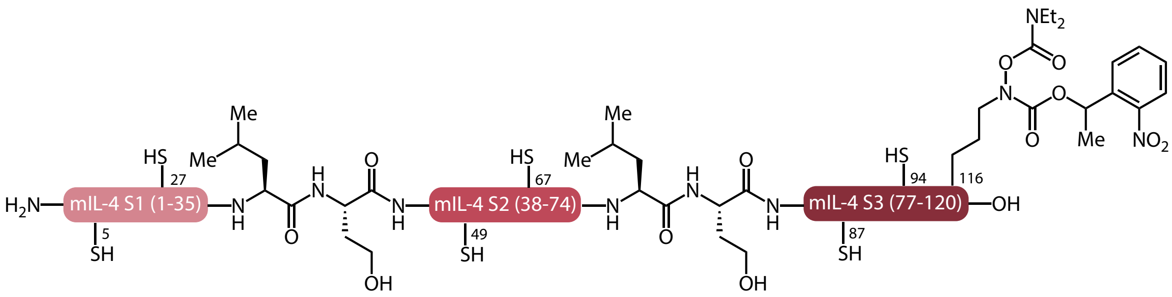

Polypeptide **S11** (12.2 mg, 861 nmol) was dissolved in 50% aq. AcOH (2.9 mL, 0.3 mM) and added AgOAc (29.0 mg, 174 µmol). After shaking at 50 °C for 1 h in the dark, the mixture was cooled to room temperature and DTT (29.0 mg, 188 µmol) added. The resulting precipitate was separated after centrifugation and the precipitate was washed twice with 50% aq. AcOH. The combined supernatant was purified by preparative RP-HPLC using Shiseido Capcell Pak MGII C18 column (5 µm, 120 Å pore size, 20 mm I.D. x 250 mm) at 60 °C with a gradient of 20 to 70% CH_3CN with 0.1% TFA over 28 min to obtain **S12** (8.2 mg, 69 %) as a white fluffy lyophilized powder.

HRMS (ESI) *m/z* calcd for $C_{602}H_{986}N_{166}O_{188}S_6$ $[M+10H]^{10+}$ 1375.9771, $[M+11H]^{11+}$ 1250.9798, $[M+12H]^{12+}$ 1146.8154, $[M+13H]^{13+}$ 1058.6764, [M] 13749.1225, found *m/z* 1375.9170, 1250.9248, 1146.8470, 1058.6819, 13749.0998.

a) b)

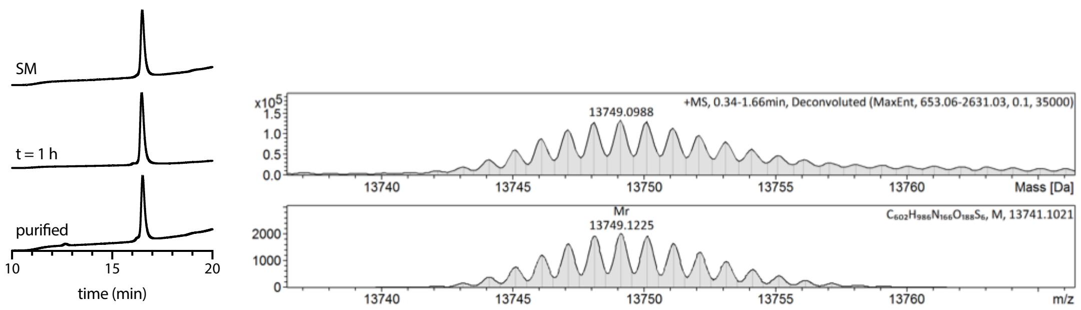

Fig. S29. Analytical data of S12. a) Analytical HPLC trace of the reaction (20 to 95% CH_3CN with 0.1% TFA over 14 min on a Shiseido Capcell Pak C18 UG120, λ = 220 nm). b) HRMS (ESI) of **S12**. Deconvoluted (top), calculated (bottom).

Synthesis of folded H-[Orn(photoHA)^{116}]-IL-4(1-120)-OH 10

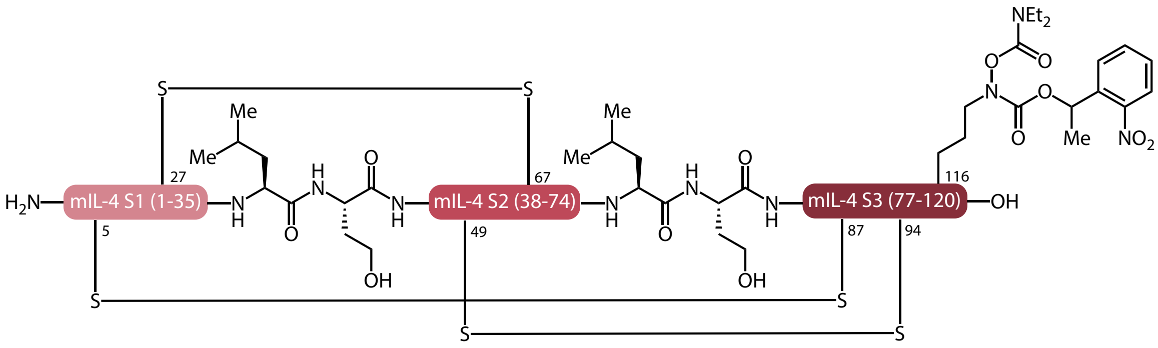

Polypeptide **S12** (4.4 mg, 0.32 µmol) was dissolved in solubilizing buffer 8.8 mL (0.5 mg/mL) containing 4 M Gn·HCl, 50 mM tris and 5 mM EDTA (pH 8.5). This solution was then diluted with 44 mL of folding buffer containing 0.5 M Arg·HCl, 50 mM tris, 2 mM Cys (pH 8.5) and incubated 18 h at room temperature. The mixture was acidified with 6 mL of 50% aq. AcOH and purified by preparative RP-HPLC using Shiseido Proteonavi column (5 µm, 300 Å pore size, 20 mm I.D. x 250 mm) at room temperature with a

gradient of 20 to 75% CH_3CN with 0.1% TFA over 28 min to obtain folded IL-4 variant **10** (1.19 mg, 27%, calculated based on BCA assay) as a white fluffy lyophilized powder.

HRMS (ESI) *m/z* calcd for $C_{602}H_{980}N_{166}O_{188}S_6$ $[M+7H]^{7+}$ 1964.3858, $[M+8H]^{8+}$ 1718.9635, $[M+9H]^{9+}$ 1528.0795, [M] 13743.0756, found *m/z* 1964.3024, 1718.8925, 1528.0166, 13743.0806.

a) b)

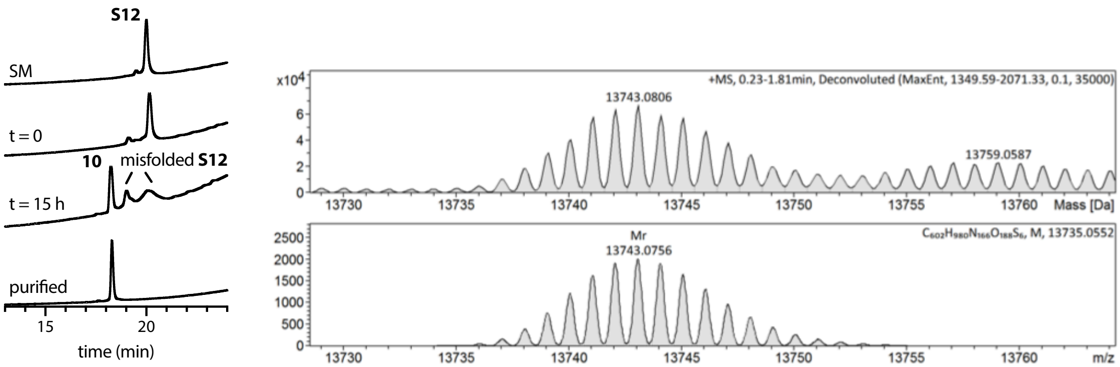

Fig. S30. Analytical data of 10. a) Analytical HPLC of the folding (20 to 80% CH_3CN with 0.1% TFA over 14 min on a Shiseido proteonavi, λ = 220 nm). b) HRMS (ESI) of **10**. Deconvoluted (top), calculated (bottom).

2.6. Synthesis of mouse IL-4 S113Orn(photoHA) S17

Synthesis of Opr-[Cys(Acm)^{87,94}, Orn(photoHA)^{113}]-IL-4(77–120)-OH S13

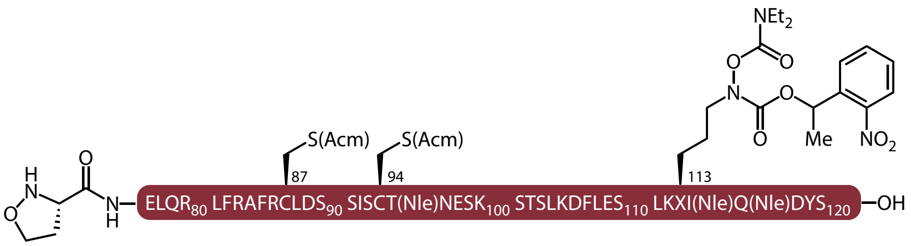

Opr-[Cys(Acm)^{87,94}, Orn-photoHA^{113}]-IL-4(77–120)-OH **S13** was synthesized on HMPB ChemMatrix resin loaded with Fmoc-Ser(tBu)-OH followed by Fmoc-Tyr(tBu)-OH (220 mg, 0.271 mmol/g, 60 µmol). Pseudoprolines Fmoc-Ile-Ser(Ψ(Me,Me)pro)-OH and Fmoc-Ser-Thr(Ψ(Me,Me)pro)-OH were used at Ile92-Ser93 and Ser101-Thr102. Fmoc-Orn(photoHA)-OH **A** was used for the coupling at Ser113 and double coupling was applied for coupling of Fmoc-Ser-Thr(Ψ(Me,Me)pro)-OH. After Fmoc-SPPS, the peptide was cleaved from the resin using cleavage cocktail B. The resulting crude peptide was purified by preparative RP-HPLC using Shiseido Capcell Pak UG80 C18 column (50 x 250 mm) at 60 °C with a gradient of 20 to 70% CH_3CN with 0.1% TFA over 40 min to obtain peptide segment **S13** (24.1 mg, 7.1%) as a white fluffy lyophilized powder.

HRMS (ESI) *m/z* calcd for $C_{246}H_{393}N_{65}O_{82}S_2$ $[M+3H]^{3+}$ 1880.1019, $[M+4H]^{4+}$ 1410.3282, $[M+5H]^{5+}$ 1128.4641, $[M+6H]^{6+}$ 940.5546, [M] 5636.8102, found *m/z* 1879.9479, 1410.2129, 1128.3723, 940.4782, 5636.8303.

a) b)

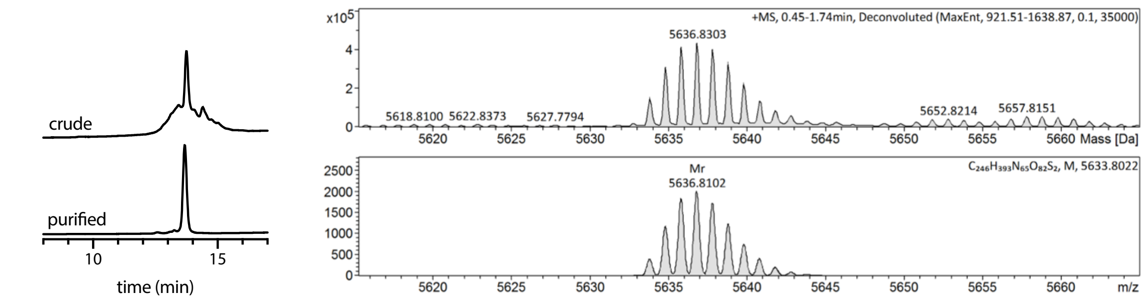

Fig. S31. Analytical data of S13. a) Analytical HPLC trace of the crude and purified **S13** (20 to 95% CH_3CN with 0.1% TFA over 14 min on a Shiseido Capcell Pak C18 UG120, λ = 220 nm). b) HRMS (ESI) of **S13**. Deconvoluted (top) and calculated (bottom).

Synthesis of H_2N-[Cys(Acm)^{5,27,49,67,87,94}, Orn(photoHA)^{113}]-IL-4(1-120)-OH containing isopeptide bond at Leu^{36}-Hse^{37} and Leu^{75}-Hse^{76} S14

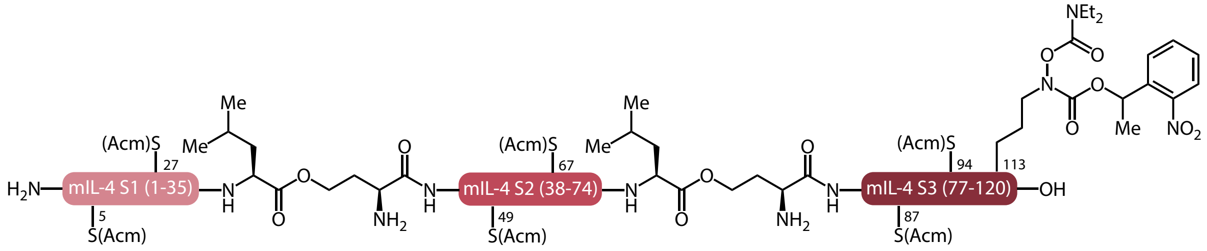

Polypeptide **4** (15.0 mg, 1.73 µmol, 1.0 equiv) and segment **S13** (12.8 mg, 2.27 µmol, 1.3 equiv) were dissolved in 9:1 DMSO:H_2O with 0.1 M oxalic acid (174 µL, 10 mM). After stirring for 24 h at 60 °C, the resulting gel-like mixture was dissolved with DMSO (400 µL) and purified by preparative RP-HPLC using Shiseido Capcell Pak MGII C18 column (5 µm, 120 Å pore size, 20 mm I.D. x 250 mm) at 60 °C with a gradient of 20 to 70% CH_3CN with 0.1% TFA over 28 min to obtain **S14** (8.4 mg, 34%) as a white fluffy lyophilized powder.

HRMS (ESI) *m/z* calcd for $C_{622}H_{1019}N_{173}O_{194}S_6$ $[M+7H]^{7+}$ 2032.0384, $[M+8H]^{8+}$ 1778.1595, $[M+9H]^{9+}$ 1580.6982, $[M+10H]^{10+}$ 1422.7291, $[M+11H]^{11+}$ 1293.4817, $[M+12H]^{12+}$ 1185.7755, $[M+13H]^{13+}$ 1094.6395, $[M+14H]^{14+}$ 1016.5229, [M] 14216.3712, found *m/z* 2031.9163, 1778.0539, 1580.7158, 1422.6451, 1293.4057, 1185.7057, 1094.7057, 1016.5342, 14217.3735.

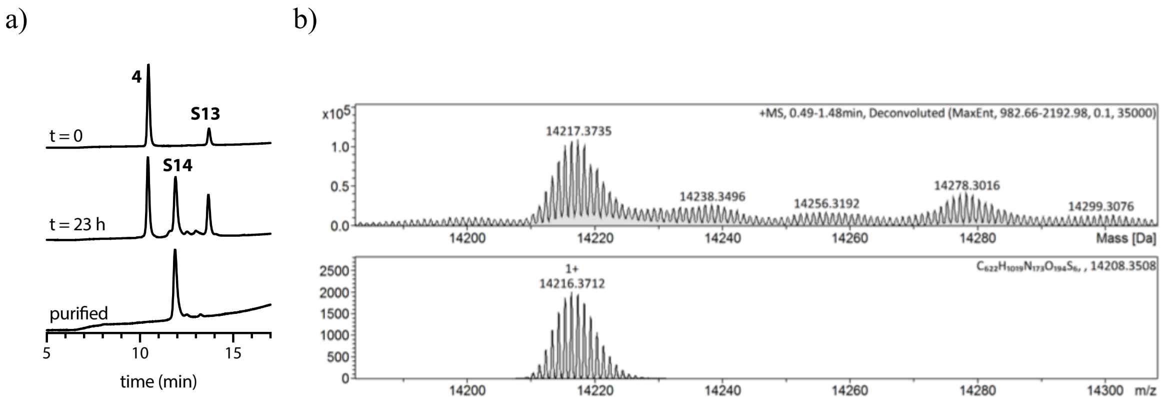

Fig. S32. Analytical data of S14. a) Analytical HPLC trace of the KAHA ligation (20 to 95% CH_3CN with 0.1% TFA over 14 min on a Shiseido Capcell Pak C18 UG120, λ = 220 nm). b) HRMS (ESI) of **S14**. Deconvoluted (top) and calculated (bottom).

Synthesis of H_2N-[Cys(Acm)^{5,27,49,67,87,94}, Orn-photoHA^{113}]-IL-4(1-120)-OH S15

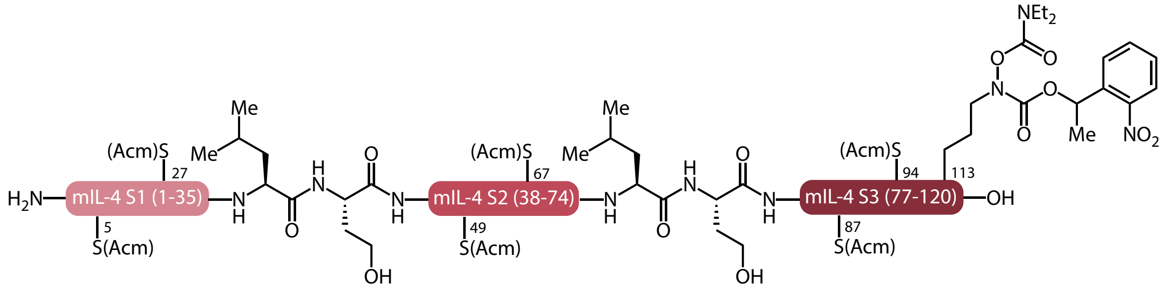

Peptide **S14** (8.4 mg, 591 nmol) was dissolved in a buffer containing 50 mM sodium carbonate and 6 M Gn·HCl pH 9.5 (3.0 mL, 0.2 mM). After incubating for 2 h at room temperature, the mixture was acidified by adding 50% aq. AcOH and purified by preparative RP-HPLC using Shiseido Capcell Pak MGII C18 column (5 μm, 120 Å pore size, 20 mm I.D. x 250 mm) at 60 °C with a gradient of 20 to 70% CH_3CN with 0.1% TFA over 28 min to obtain **S15** (6.1 mg, 73%) as a white fluffy lyophilized powder.

HRMS (ESI) *m/z* calcd for $C_{622}H_{1019}N_{173}O_{194}S_6$ [M] 14216.3712, found *m/z* 14216.3721.

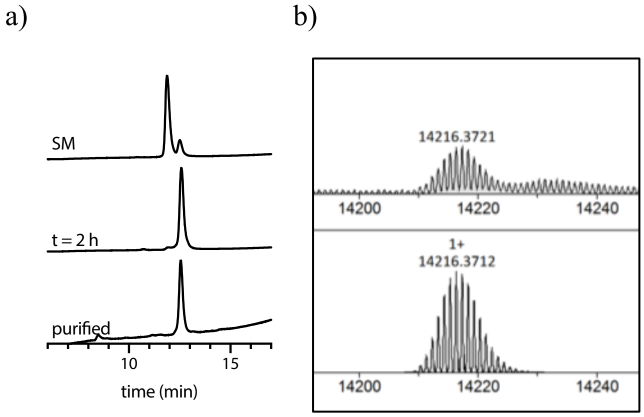

Fig. S33. Analytical data of S15. a) Analytical HPLC trace of the reaction (20 to 95% CH_3CN with 0.1% TFA over 14 min on a Shiseido Capcell Pak C18 UG120, λ = 220 nm). b) HRMS (ESI) of **S15**. Deconvoluted (top) and calculated (bottom).

Synthesis of H_2N-[Orn-photoHA^{113}]-IL-4(1-120)-OH S16

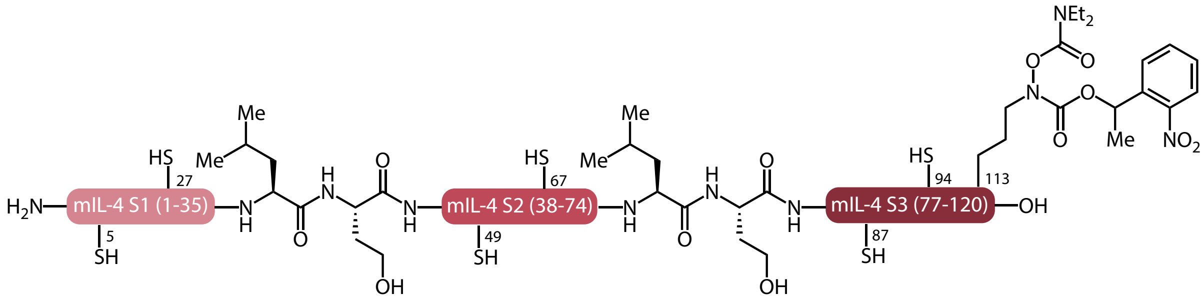

Polypeptide **S15** (6.1 mg, 429 nmol) was dissolved in 50% aq. AcOH (1.4 mL, 0.3 mM) and added AgOAc (14.0 mg, 83.9 µmol). After shaking at 50 °C for 1 h in the dark, the mixture was cooled to room temperature and added DTT (14.0 mg, 97.2 µmol). The formed precipitate was separated after centrifugation and the precipitate was washed twice with 50% aq. AcOH. The combined supernatant was purified by preparative RP-HPLC using Shiseido Capcell Pak MGII C18 column (5 µm, 120 Å pore size, 20 mm I.D. x 250 mm) at 60 °C with a gradient of 20 to 70% CH_3CN with 0.1% TFA over 28 min to obtain **S16** (4.1 mg, 69 %) as a white fluffy lyophilized powder.

HRMS (ESI) *m/z* calcd for $C_{604}H_{989}N_{167}O_{188}S_6$ $[M+7H]^{7+}$ 1971.1143, $[M+8H]^{8+}$ 1724.8510, $[M+9H]^{9+}$ 1533.3128, $[M+10H]^{10+}$ 1380.0823, $[M+11H]^{11+}$ 1254.7118, $[M+12H]^{12+}$ 1150.2364, $[M+13H]^{13+}$ 1061.8342, [M] 13790.1491, found *m/z* 1971.0249, 1724.7732, 1533.3552, 1380.0206, 1254.7462, 1150.2684, 1061.7866, 13790.1350.

a) b)

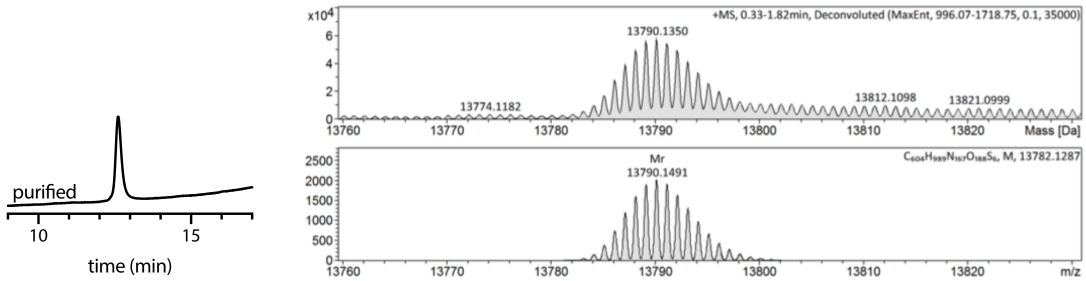

Fig. S34. Analytical data of S16. a) Analytical HPLC trace of purified **S16** (20 to 95% CH_3CN with 0.1% TFA over 14 min on a Shiseido Capcell Pak C18 UG120, λ = 220 nm). b) HRMS (ESI) of **S16**. Deconvoluted (top), calculated (bottom).

Synthesis of folded H-[Orn-photoHA^{113}]-IL-4(1-120)-OH S17

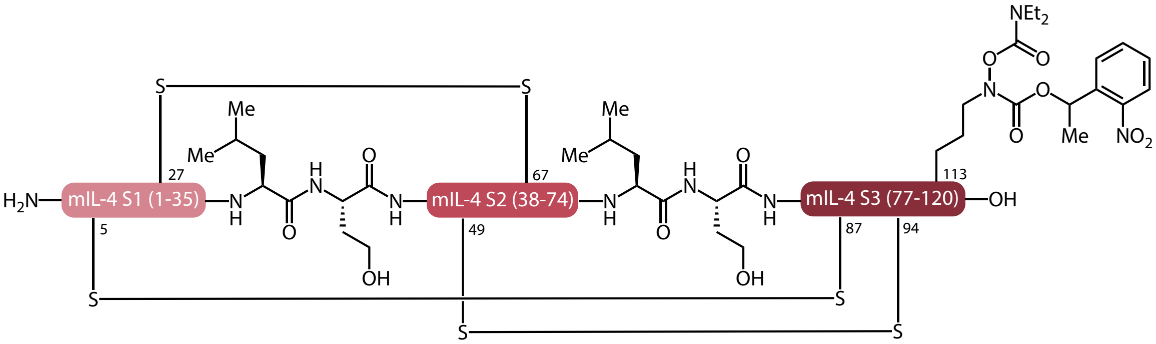

Polypeptide **S16** (4.1 mg, 0.30 µmol) was dissolved in solubilizing buffer 8.2 mL (0.5 mg/mL) containing 4 M Gn·HCl, 50 mM tris and 5 mM EDTA (pH 8.5). This solution was diluted with 41 mL of folding buffer containing 0.5 M Arg·HCl, 50 mM tris, 2 mM Cys (pH 8.5) and incubated 18 h at room temperature. The mixture was acidified with 6 mL of 50% aq. AcOH and purified by preparative RP-HPLC using Shiseido Capcell Pak MGII C18 column (5 µm, 120 Å pore size, 20 mm I.D. x 250 mm) at 60 °C with a gradient of 20 to 75% CH_3CN with 0.1% TFA over 28 min to obtain folded IL-4 variant **S17** as a white fluffy lyophilized powder.

HRMS (ESI) *m/z* calcd for $C_{604}H_{983}N_{167}O_{188}S_6$ $[M+6H]^{6+}$ 2298.4576, $[M+7H]^{7+}$ 1970.2504, $[M+8H]^{8+}$ 1724.0950, $[M+9H]^{9+}$ 1532.6408, $[M+10H]^{10+}$ 1379.4775, [M] 13784.1021, found *m/z* 2298.3556, 1970.1647, 1724.0216, 1532.6881, 1379.4196, 13784.1111.

a) b)

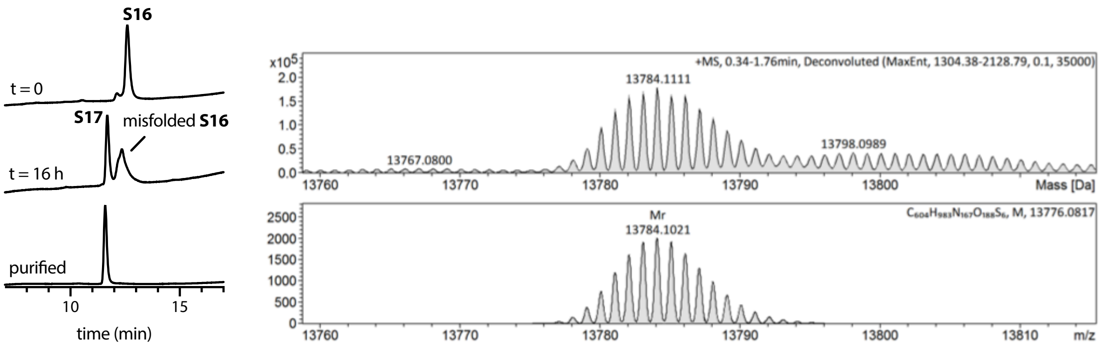

Fig. S35. Analytical data of S17. a) Analytical HPLC of the folding (20 to 95% CH_3CN with 0.1% TFA over 14 min on a Shiseido Capcell Pak C18 UG120, λ = 220 nm). b) HRMS (ESI) of **S17**. Deconvoluted (top), calculated (bottom).

2.7. Synthesis of PEGylated IL-4

Synthesis of IL-4 Q116Orn(PEG-10kDa) 11

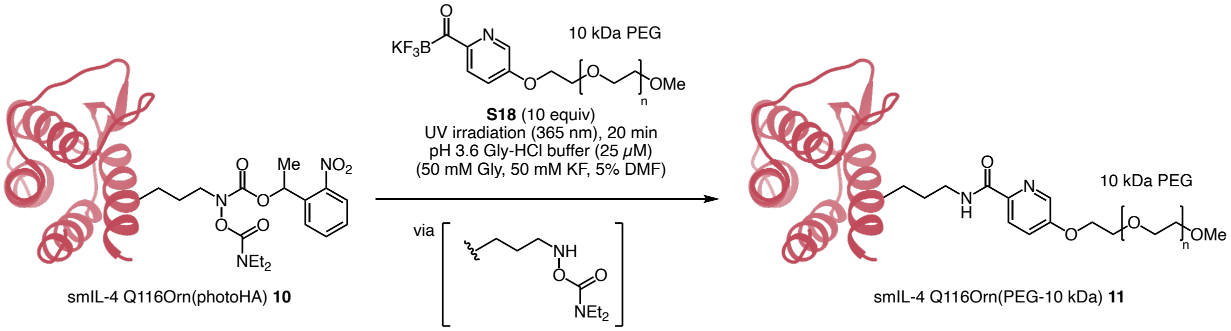

Folded IL-4 containing photoprotected hydroxylamine at Q116 **10** (200 μg, 14.6 nmol, 1.0 equiv) was dissolved in 582 μL of the ligation buffer containing 50 mM Gly·HCl, 50 mM KF and 5% DMF (pH 3.6, 25 μM). To this solution was added 10kDa-PEG-pyridyl KAT **S18** (14.6 μL, 146 nmol, 10 equiv, 10 mM in DMF) which was prepared from 10 kDa poly(ethylene glycol) mono-methyl ether (average M_n ~10000) according to the reported procedure[35] and the mixture was irradiated with handheld UV lamp at a wavelength of 365 nm for 20 min at room temperature. After incubating for 21 h at room temperature, the mixture was diluted with 1:1 CH_3CN:H_2O with 0.1% TFA and purified by preparative RP-HPLC using Shiseido Capcell Pak MGII column (5 μm, 120 Å pore size, 10 mm I.D. x 250 mm) at 60 °C with a gradient of 20 to 85% CH_3CN with 0.1% TFA over 30 min to obtain IL-4 Q116Orn(PEG-10 kDa) **11** (252 μg, 74% isolated yield, calculated based on the BSA assay).

a) b)

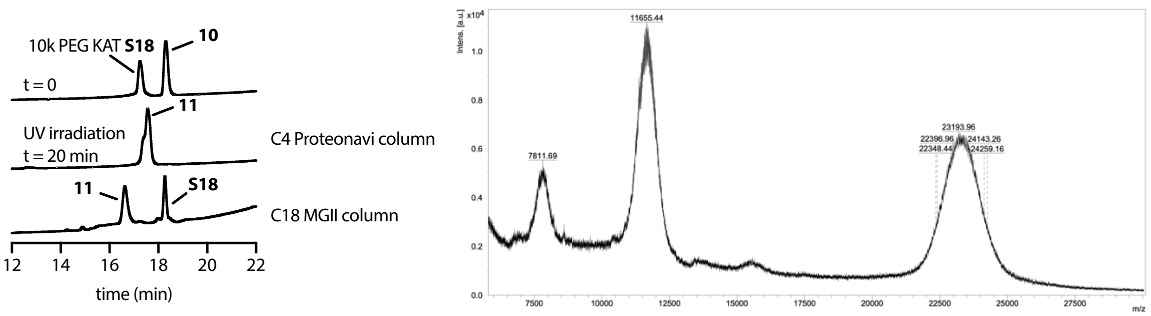

Fig. S36. Analytical data of 11. a) Analytical HPLC of PEGylation (20 to 95% CH_3CN with 0.1% TFA over 14 min on a Shiseido Proteonavi and Capcell Pak C18 MGII, λ = 220 nm). b) MS (MALDI-TOF) of **11**.

Synthesis of IL-4 S113Orn(PEG-10kDa) 12

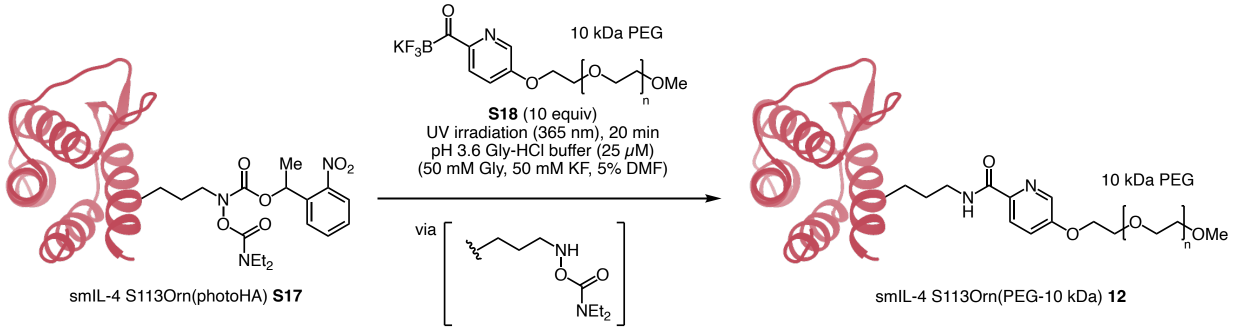

Folded IL-4 containing photoprotected hydroxylamine at S113 **S17** (200 µg, 14.5 nmol, 1.0 equiv) was dissolved in 500 µL of the ligation buffer containing 50 mM Gly·HCl, 50 mM KF and 5% DMF (pH 3.6, 29 µM). To this solution was added 10kDa-PEG-pyridyl KAT **S18** (18.0 µL, 180 nmol, 12 equiv, 10 mM in DMF) and the mixture was irradiated with hand-held UV lamp at a wavelength of 365 nm for 30 min at room temperature. After incubating for 15 h at room temperature, the mixture was diluted with 1:1 CH_3CN:H_2O with 0.1% TFA and purified by preparative RP-HPLC using Shiseido Capcell Pak MGII column (5 µm, 120 Å pore size, 10 mm I.D. x 250 mm) at 60 °C with a gradient of 20 to 85% CH_3CN with 0.1% TFA over 30 min to obtain IL-4 S113Orn(PEG-10 kDa) **12** (21 µg, 6.3% isolated yield, calculated based on the BSA assay).

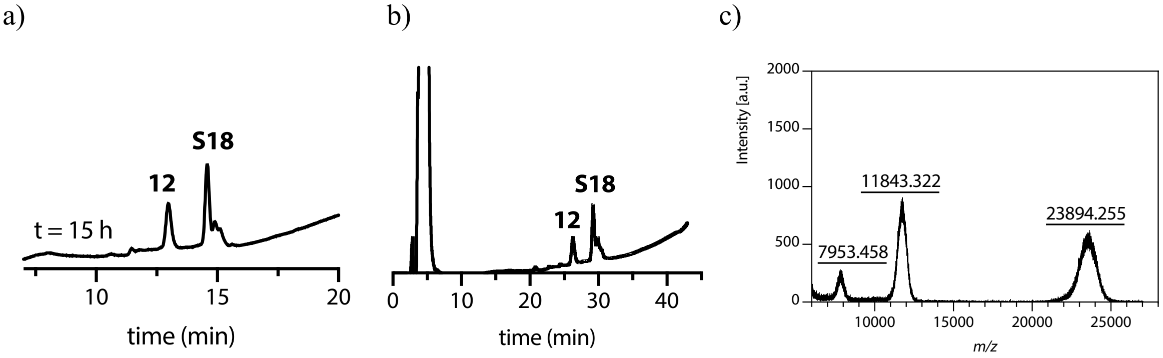

Fig. S37. Analytical data of 12. a) Analytical HPLC of PEGylation (20 to 95% CH_3CN with 0.1% TFA over 14 min on a Shiseido Capcell Pak C18 UG120, λ = 220 nm). b) Preparative HPLC of PEGylation (20 to 85% CH_3CN with 0.1% TFA over 30 min on a Shiseido Capcell Pak MGII column, λ = 220 nm). c) MS (MALDI-TOF) of **12**.

2.8. Synthesis of Fmoc-Gln(photocaged)-OH 13

2-Nitroacetophenone *O*-methyl oxime S19

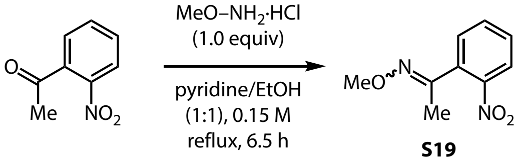

S19

A solution of 2-nitroacetophenone (2.0 g, 12 mmol) and methoxyamine hydrochloride (1.0 g, 12 mmol) in pyridine/EtOH (80 mL, 1:1, v/v) was refluxed for 6.5 h. After cooling to room temperature, the volatiles were removed under vacuum. The residue was treated with EtOAc and H_2O, and separated. The organic layer was washed with saturated aqueous $NaHCO_3$ and brine, dried ($MgSO_4$), and concentrated under vacuum. The resulting crude mixture of 2-nitroacetophenone and oxime **S19** (2.31 g, ca. 1:4.3, determined by ^{1}H-NMR analysis) was used for the next reaction without purification.

1-(2-nitrophenyl)ethan-1-amine hydrochloride S20

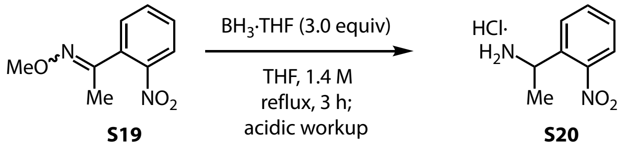

S19 **S20**

58% over 2 steps

To a solution of 2-nitroacetophenone and oxime **S19** (2.31 g, crude) in THF (8.5 mL) under N_2 atmosphere was added borane·THF complex (1.0 M in THF, 35.7 mmol) at 0 °C. After refluxing for 3 h, the reaction mixture was cooled to –20 °C and added H_2O (2 mL). Then, 20% aqueous KOH (2.0 mL) was added over 20 min followed by refluxing for 2 h. After cooling to room temperature, the mixture was extracted with CH_2Cl_2 (x3). The combined organic layers were washed with brine, dried ($MgSO_4$), and concentrated under vacuum. The residue was suspended in CH_2Cl_2 (30 mL) and acidified with conc. HCl (1.5 mL) while stirring. The resulting precipitates were filtered, washed with Et_2O and dried under vacuum to obtain 1-(2-nitrophenyl)ethan-1-amine hydrochloride **S20** (1.41 g, 58% over 2 steps) as a white solid.

Analytical data matched the literature.[56]

Fmoc-Gln(photocaged)-OtBu S22

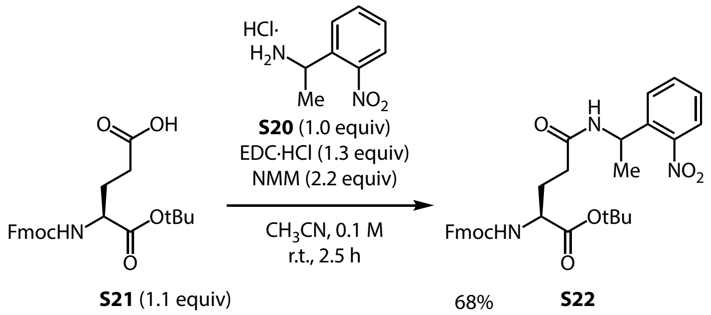

To a solution of Fmoc-Glu-OtBu **S21** (2.0 g, 4.7 mmol) in CH_3CN (47 mL) was added EDC·HCl (1.08 g, 5.64 mmol), **S20** (859 mg, 4.24 mmol), and NMM (1.2 mL, 9.4 mmol). After stirring for 2.5 h, the reaction was diluted with EtOAc and H_2O, and the phases were separated. The aqueous phase was extracted with EtOAc (x3) and the combined organic layers were washed with brine, dried ($MgSO_4$), and concentrated under vacuum. The residue was purified by flash column chromatography (silica gel, hexanes/EtOAc = 1/1) to obtain Fmoc-Gln(photocaged)-OtBu **S22** (1.84 g, 76%) as a colorless oil.

S22 (a mixture of two diastereomers): Rf 0.63 (hexanes/EtOAc = 1/1); ^{1}H NMR (400 MHz, $CDCl_3$) δ 7.87 – 7.84 (m, 1H), 7.79 – 7.76 (m, 2H), 7.62 – 7.59 (m, 2H), 7.55 – 7.48 (m, 2H), 7.43 – 7.30 (m, 5H), 6.78 – 6.69 (m, 1H), 4.43 – 4.40 (m, 2H), 4.24 – 4.11 (m, 2H), 2.24 – 2.20 (m, 3H), 1.85 – 1.81 (m, 1H), 1.56 – 1.53 (m, 3H), 1.46 – 1.43 (m, 9H); ^{13}C NMR (100 MHz, $CDCl_3$) δ 171.12, 171.02, 156.58, 148.70, 148.62, 143.89, 143.62, 141.33, 139.13, 138.93, 133.30, 128.29, 127.93, 127.87, 127.80, 127.79, 127.13, 127.12, 125.12, 124.77, 120.05, 82.81, 82.67, 77.40, 77.08, 76.76, 67.10, 53.82, 53.73, 47.20, 46.32, 32.35, 29.41, 29.30, 28.00, 27.97, 21.59, 21.51; IR (ATR) 3319, 1727, 1686, 1643, 1521, 1445, 1336, 1253, 1232, 1151, 1034 cm^{-1}; HRMS (ESI) calcd for $C_{32}H_{36}N_3O_7$ ($[M+H]^+$) *m/z* 574.2548, found *m/z* 574.2540.

Fmoc-Gln(photocaged)-OH (photoQ) 13

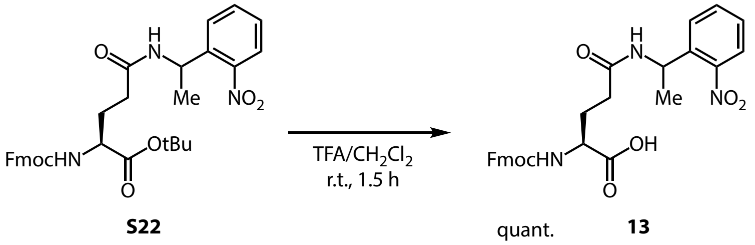

To a solution of **S22** in CH_2Cl_2 (20 mL) was added TFA (20 mL). After stirring for 1.5 h at room temperature, the reaction mixture was evaporated under vacuum. The residue was dissolved in CH_3CN/H_2O (1:1) with 0.1% TFA and lyophilized to obtain Fmoc-Gln(photocaged)-OH **13** (1.64 g, quant.) as a yellow lyophilized powder.

13 (a mixture of two diastereomers): ^{1}H NMR (400 MHz, DMSO) δ 8.62 (t, *J* = 7.5 Hz, 1H), 7.95 – 7.83 (m, 3H), 7.78 – 7.57 (m, 5H), 7.51 – 7.37 (m, 3H), 7.37 – 7.28 (m, 2H), 5.29 – 5.07 (m, 1H), 4.39 – 4.11 (m, 3H), 3.97 – 3.77 (m, 1H), 2.19 (dtd, *J* = 15.3, 8.8, 7.2, 3.9 Hz, 2H), 1.92 (dt, *J* = 6.9, 4.4 Hz, 1H), 1.79 – 1.62 (m, 1H), 1.40 (dd, *J* = 7.0, 1.1 Hz, 3H); ^{13}C NMR (100 MHz, DMSO) δ 173.69, 173.67, 170.73, 170.69, 156.18, 156.16, 148.24, 148.20, 143.84, 140.74, 139.99, 133.65, 133.59, 127.94, 127.92, 127.69, 127.58, 127.53, 127.13, 125.34, 123.81, 123.80, 120.16, 65.70, 53.39, 53.35, 46.65, 44.05, 43.95, 31.53, 31.48, 26.54, 26.49, 21.67; IR (ATR) 3319, 2979, 1688, 1640, 1523, 1164, 738 cm^{-1}; HRMS (ESI) calcd for $C_{28}H_{28}N_3O_7$ ($[M+H]^+$) *m/z* 518.1922, found *m/z* 518.1918.

2.9. Synthesis of mouse IL-4 Q116photoQ 14

Synthesis of Opr-[Cys(Acm)[87,94], photoQ[116]]-IL-4(77–120)-OH S23

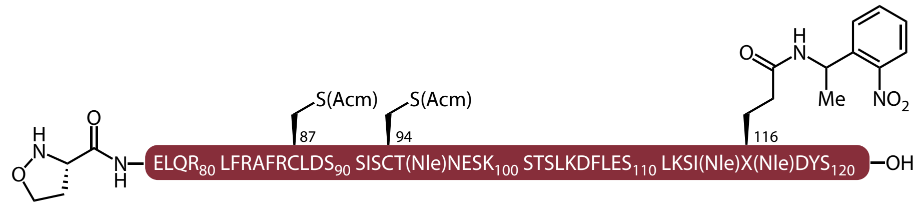

Opr-[Cys(Acm)[87,94], photoQ[116]]-IL-4(77–120)-OH **S23** was synthesized on HMPB ChemMatrix resin loaded with Fmoc-Ser(tBu)-OH followed by Fmoc-Tyr(tBu)-OH (400 mg, 0.271 mmol/g, 108 µmol). Pseudoprolines Fmoc-Ile-Ser(Ψ(Me,Me)pro)-OH and Fmoc-Ser-Thr(Ψ(Me,Me)pro)-OH were used at Ile92-Ser93 and Ser101-Thr102. Fmoc-Gln(photocaged)-OH **13** was used for the coupling at Gln116. Double couplings were applied for coupling of Fmoc-Ile-Ser(Ψ(Me,Me)pro)-OH, Fmoc-Ser-Thr(Ψ(Me,Me)pro)-OH, Fmoc-Cys(Acm)-OH at Cys87, and Fmoc-Cys(Acm)-OH at Cys94. After Fmoc-SPPS, the peptide was cleaved from the resin using cleavage cocktail B. The resulting crude peptide was purified by preparative RP-HPLC using Shiseido Capcell Pak UG80 C18 column (50 x 250 mm) at 60 °C with a gradient of 20 to 70% CH_3CN with 0.1% TFA over 40 min to obtain peptide segment **S23** (35.1 mg, 5.9%) as a white fluffy lyophilized powder.

HRMS (ESI) *m/z* calcd for $C_{241}H_{385}N_{63}O_{78}S_2$ $[M+3H]^{3+}$ 1826.7258, $[M+4H]^{4+}$ 1370.2962, $[M+5H]^{5+}$ 1096.4384, $[M+6H]^{6+}$ 913.8666, [M] 5476.7712, found *m/z* 1826.5964, 1370.2000, 1096.3608, 913.8015, 5476.7712.

a) b)

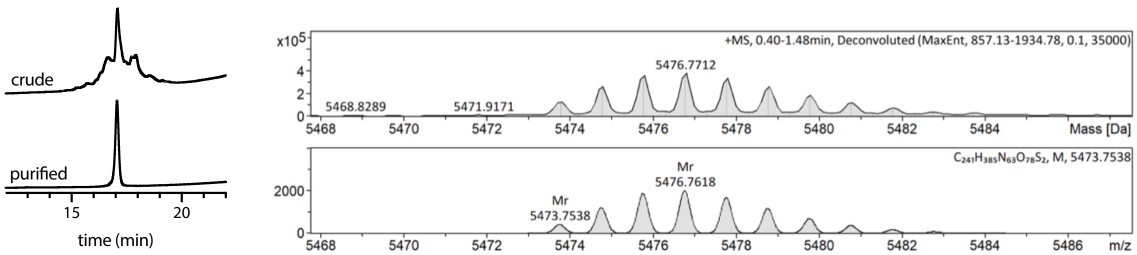

Fig. S38. Analytical data of S23. a) Analytical HPLC trace of the crude and purified **S23** (20 to 95% CH_3CN with 0.1% TFA over 14 min on a Shiseido Capcell Pak C18 UG120, λ = 220 nm). b) HRMS (ESI) of **S23**. Deconvoluted (top) and calculated (bottom).

Synthesis of H$_2$N-[Cys(Acm)^{5,27,49,67,87,94}, photoQ^{116}]-IL-4(1-120)-OH containing isopeptide bond at Leu^{36}-Hse^{37} and Leu^{75}-Hse^{76} S24

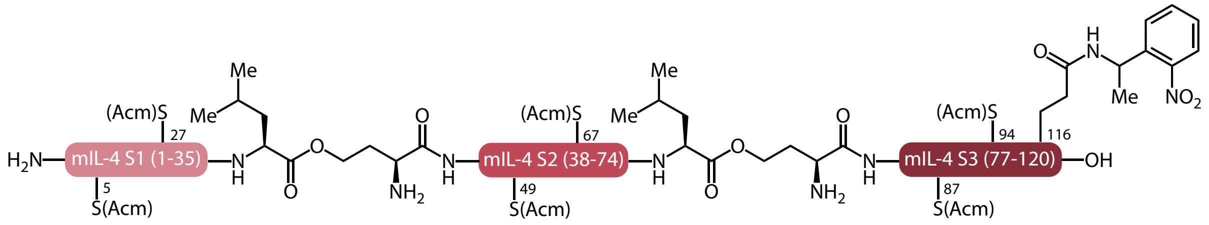

Polypeptide **4** (23.1 mg, 2.68 µmol, 1.0 equiv) and segment **S23** (19.1 mg, 3.49 µmol, 1.3 equiv) were dissolved in 9:1 DMSO:H$_2$O with 0.1 M oxalic acid (268 µL, 10 mM). After stirring for 24 h at 60 °C, the resulting gel-like mixture was dissolved with DMSO (1 mL) and purified by preparative RP-HPLC using Shiseido Capcell Pak MGII C18 column (5 µm, 120 Å pore size, 20 mm I.D. x 250 mm) at 60 °C with a gradient of 20 to 70% CH$_3$CN with 0.1% TFA over 28 min to obtain **S24** (10.8 mg, 29%) as a white fluffy lyophilized powder.

HRMS (ESI) m/z calcd for $C_{614}H_{1005}N_{171}O_{191}S_6$ $[M+13H]^{13+}$ 1080.3157, $[M+14H]^{14+}$ 1003.2223, $[M+15H]^{15+}$ 936.4079, $[M+16H]^{16+}$ 877.9454, $[M+17H]^{17+}$ 826.3608, $[M+18H]^{18+}$ 780.5078, [M] 14030.2713, found m/z 1080.2603, 1003.1709, 936.3601, 877.9005, 826.3186, 780.4680, 14030.2895.

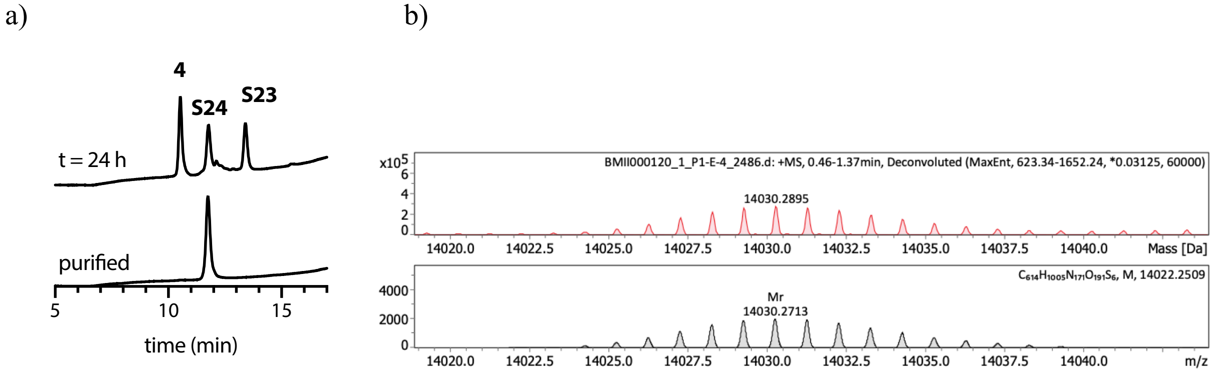

Fig. S39. Analytical data of S24. a) Analytical HPLC trace of the KAHA ligation (20 to 95% CH$_3$CN with 0.1% TFA over 14 min on a Shiseido Capcell Pak C18 UG120, λ = 220 nm). b) HRMS (ESI) of **S24**. Deconvoluted (top) and calculated (bottom).

Synthesis of H$_2$N-[Cys(Acm)^{5,27,49,67,87,94}, photoQ^{116}]-IL-4(1-120)-OH S25

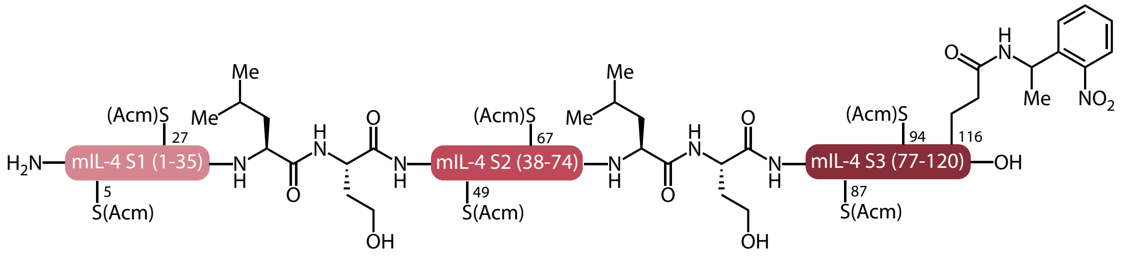

Peptide **S24** (10.8 mg, 0.770 µmol) was dissolved in a buffer containing 50 mM CHES and 6 M Gn·HCl pH 9.5 (3.9 mL, 0.2 mM). After incubating for 2.5 h at room temperature, the mixture was acidified by adding 50% aq. AcOH and purified by preparative RP-HPLC using Shiseido Capcell Pak MGII C18 column (5 µm, 120 Å pore size, 20 mm I.D. x 250 mm) at 60 °C with a gradient of 20 to 70% CH_3CN with 0.1% TFA over 28 min to obtain **S25** (8.6 mg, 80%) as a white fluffy lyophilized powder.

HRMS (ESI) *m/z* calcd for $C_{614}H_{1005}N_{171}O_{191}S_6$ $[M+13H]^{13+}$ 1080.3157, $[M+14H]^{14+}$ 1003.2223, $[M+15H]^{15+}$ 936.4079, $[M+16H]^{16+}$ 877.9454, $[M+17H]^{17+}$ 826.3608, [M] 14030.2713, found *m/z* 1080.2601, 1003.1707, 936.3599, 877.9003, 826.3184, 14030.2863.

a) b)

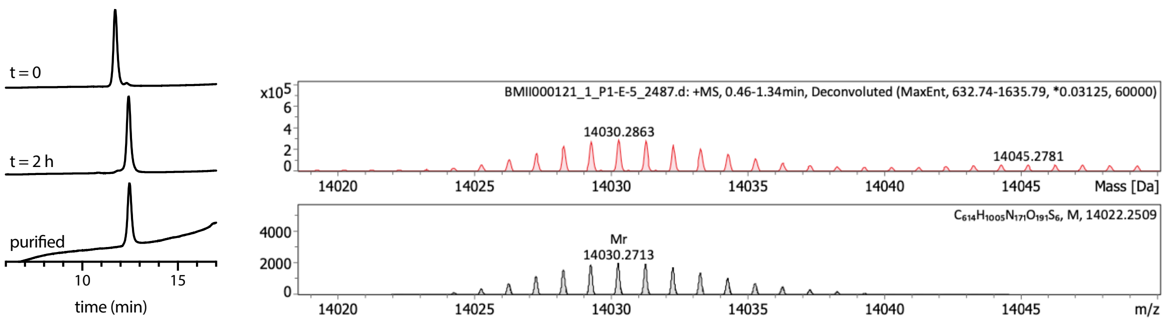

Fig. S40. Analytical data of S25. a) Analytical HPLC trace of the reaction (20 to 95% CH_3CN with 0.1% TFA over 14 min on a Shiseido Capcell Pak C18 UG120, λ = 220 nm). b) HRMS (ESI) of **S25**. Deconvoluted (top) and calculated (bottom).

Synthesis of H_2N-[photoQ^{116}]-IL-4(1-120)-OH S26

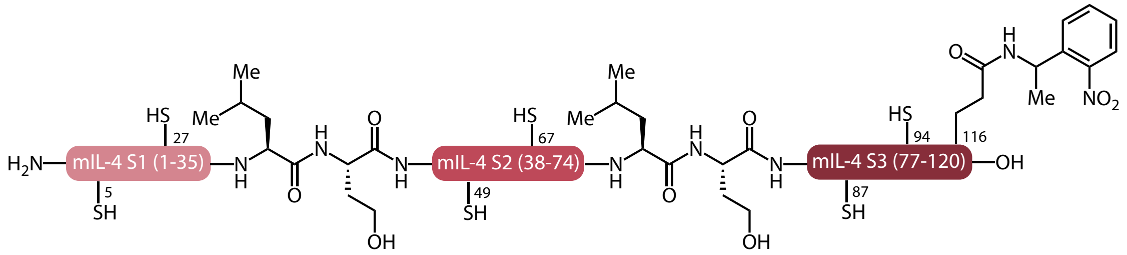

Polypeptide **S25** (8.6 mg, 613 nmol) was dissolved in 50% aq. AcOH (2.0 mL, 0.3 mM) and AgOAc (20.0 mg, 119 μmol) was added. After shaking at 50 °C for 1 h in the dark, the mixture was cooled to room temperature and DTT (20.0 mg, 129 μmol) was added. The formed precipitate was separated after centrifugation and the precipitate was washed twice with 50% aq. AcOH. The combined supernatant was purified by preparative RP-HPLC using Shiseido Capcell Pak MGII C18 column (5 μm, 120 Å pore size, 20 mm I.D. x 250 mm) at 60 °C with a gradient of 20 to 70% CH_3CN with 0.1% TFA over 28 min to obtain **S26** (5.4 mg, 65 %) as a white fluffy lyophilized powder.

HRMS (ESI) *m/z* calcd for $C_{596}H_{975}N_{165}O_{185}S_6$ $[M+8H]^{8+}$ 1701.5749, $[M+9H]^{9+}$ 1512.6229, $[M+10H]^{10+}$ 1361.4614, $[M+11H]^{11+}$ 1237.7837, $[M+12H]^{12+}$ 1134.7190, $[M+13H]^{13+}$ 1047.5105, $[M+14H]^{14+}$ 972.7602, $[M+15H]^{15+}$ 907.9767, [M] 13604.0486, found *m/z* 1701.5141, 1512.4563, 1361.4104, 1237.7350, 1134.6725, 1047.3914, 972.7192, 907.8728, 13603.9799.

a) b)

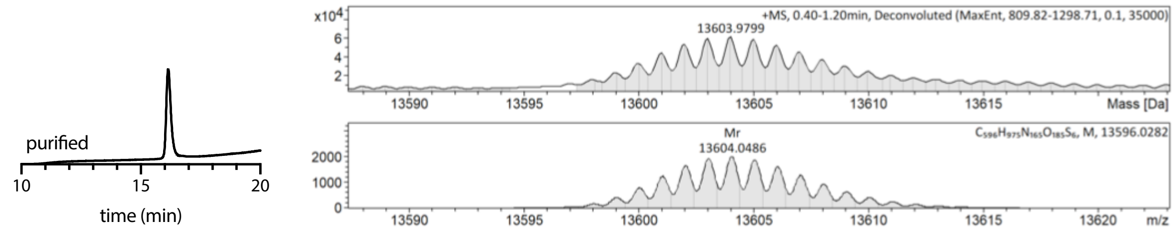

Fig. S41. Analytical data of S26. a) Analytical HPLC trace of purified **S26** (20 to 95% CH_3CN with 0.1% TFA over 14 min on a Shiseido Capcell Pak C18 UG120, λ = 220 nm). b) HRMS (ESI) of **S26**. Deconvoluted (top), calculated (bottom).

Synthesis of folded H-[photoQ^{116}]-IL-4(1-120)-OH 14

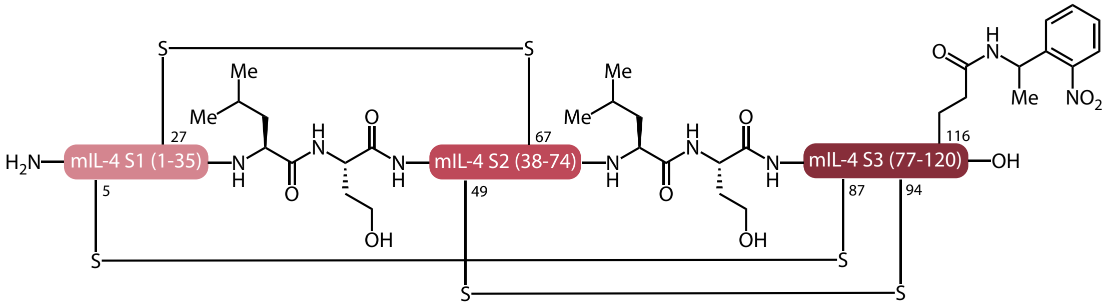

Polypeptide **S26** (5.4 mg, 0.40 µmol) was dissolved in a buffer 10.8 mL (0.5 mg/mL) containing 4 M Gn·HCl, 50 mM tris and 5 mM EDTA (pH 8.5). This solution was diluted with 54 mL of folding buffer containing 0.5 M Arg·HCl, 50 mM tris, 2 mM Cys (pH 8.5) and incubated 18 h at room temperature. The mixture was acidified with 6 mL of 50% aq. AcOH and purified by preparative RP-HPLC using Shiseido Proteonavi column (5 µm, 300 Å pore size, 20 mm I.D. x 250 mm) at room temperature with a gradient of 20 to 75% CH_3CN with 0.1% TFA over 28 min to obtain folded photocaged IL-4 **14** (1.84 mg, 34% isolated yield, calculated based on BCA assay) as a white fluffy lyophilized powder.

HRMS (ESI) *m/z* calcd for $C_{596}H_{969}N_{165}O_{185}S_6$ $[M+7H]^{7+}$ 1943.6491, $[M+8H]^{8+}$ 1700.8189, $[M+9H]^{9+}$ 1511.9510, [M] 13598.0016, found *m/z* 1943.5722, 1700.7530, 1511.8922, 13598.9399.

a) b)

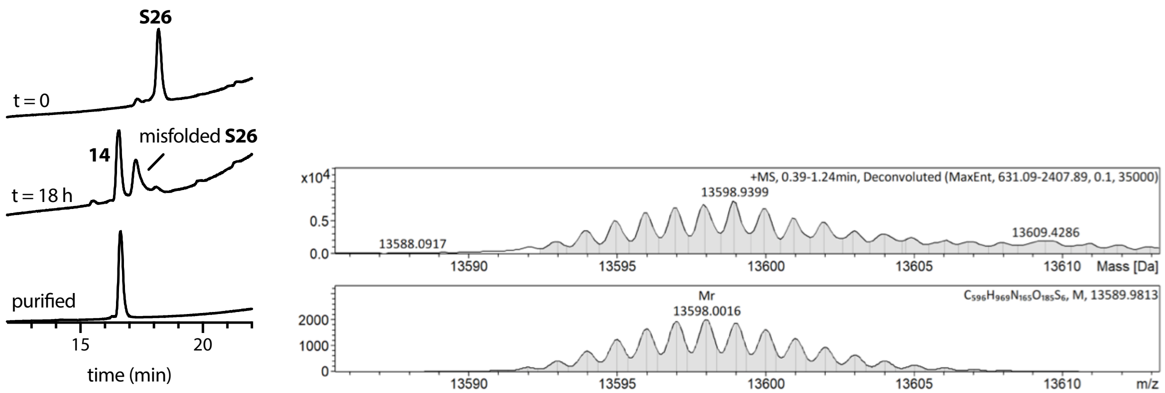

Fig. S42. Analytical data of 14. a) Analytical HPLC of the folding (20 to 95% CH_3CN with 0.1% TFA over 14 min on a Shiseido Proteonavi, λ = 220 nm). b) HRMS (ESI) of **14**. Deconvoluted (top), calculated (bottom).

2.10. Synthesis of mouse IL-4 N41PEG, Q116photoQ 16

Synthesis of Opr-[Cys(Acm)^{49,67}, AzK^{41}]-IL-4(38–74)-photoprotected-Leu-α-ketoacid S27

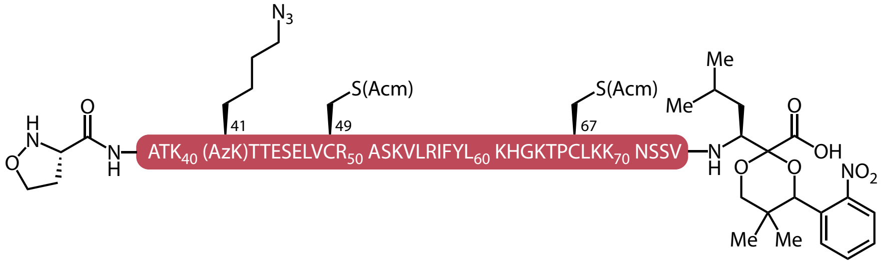

Opr-[Cys(Acm)^{49,67}, AzK^{41}]-IL-4(38–74)-photoprotected-Leu-α-ketoacid **S27** was synthesized on Rink-Amide ChemMatrix resin loaded with Fmoc-photoprotected-Leu-α-ketoacid (1.0 g, 0.22 mmol/g, 0.2 mmol). Pseudoproline Fmoc-Thr(*t*Bu)-Thr(Ψ(Me,Me)pro)-OH and Fmoc-Ala-Ser(Ψ(Me,Me)pro)-OH were used at Thr42-Thr43 and Ala51-Ser52 and Fmoc-Lys(N_3)-OH was used at Asn41. Double couplings were applied for the coupling of Fmoc-Thr(*t*Bu)-Thr(Ψ(Me,Me)pro)-OH, Fmoc-Ala-Ser(Ψ(Me,Me)pro)-OH, and Fmoc-Cys(Acm)-OH at Cys49. After Fmoc-SPPS, the peptide was cleaved from the resin using cleavage cocktail A. The resulting crude peptide was purified by preparative RP-HPLC using ReproSil-Pur 120 ODS-3 column (5 µm, 120 Å pore size, 50 mm I.D. x 250 mm) at 60 °C with a gradient of 20 to 50% CH_3CN with 0.1% TFA over 30 min to obtain peptide segment **S27** (189 mg, 18 %) as a white fluffy lyophilized powder.

HRMS (ESI) *m/z* calcd for $C_{212}H_{352}N_{60}O_{61}S_2$ $[M+2H]^{2+}$ 2391.7932, $[M+3H]^{3+}$ 1594.8646, $[M+4H]^{4+}$ 1196.4003, $[M+5H]^{5+}$ 957.3217, [M] 4780.5782, found *m/z* 2391.8052, 1594.5380, 1196.1548, 957.1260, 4780.6045.

a) b)

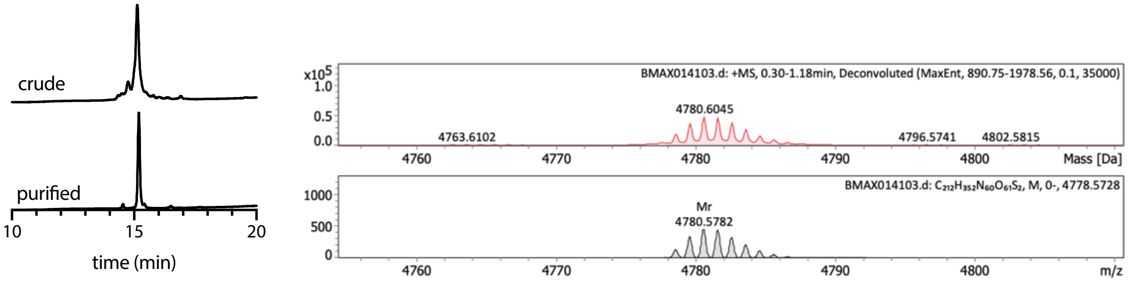

Fig. S43. Analytical data of S27. a) Analytical HPLC trace of the crude and purified **S27**. (25 to 50% CH_3CN with 0.1% TFA over 14 min on a Shiseido Capcell Pak MGIII C18, λ = 220 nm) b) HRMS (ESI) of **S27**. Deconvoluted (top) and calculated (bottom).

Synthesis of H_2N-[Cys(Acm)^{5,27,49,67}, AzK^{41}]-IL-4(1-74)-Leu-α-ketoacid containing isopeptide bond at Leu^{36}-Hse^{37} S28

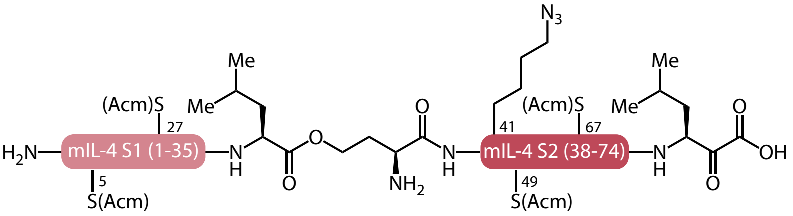

Segment **1** (55.8 mg, 13.6 µmol, 1.3 equiv) and segment **S27** (50.0 mg, 10.5 µmol, 1.0 equiv) were dissolved in 9:1 DMSO:H_2O with 0.1 M oxalic acid (523 µL, 20 mM). After stirring for 17 h at 60 °C, the mixture was cooled to room temperature, diluted with 1:1 CH_3CN:H_2O with 0.1% TFA (3.5 mL, 3 mM) and irradiated at a wavelength of 365 nm for 45 min at room temperature. This reaction mixture was purified by preparative RP-HPLC using ReproSil-Pur 120 ODS-3 column (5 µm, 120 Å pore size, 50 mm I.D. x 250 mm) at 60 °C with a gradient of 20 to 60% CH_3CN with 0.1% TFA over 30 min to obtain peptide **S28** (41.1 mg, 46 %) as a fluffy lyophilized powder.

HRMS (ESI) *m/z* calcd for $C_{376}H_{624}N_{110}O_{114}S_4$ $[M+6H]^{6+}$ 1440.6624, $[M+7H]^{7+}$ 1234.9974, $[M+8H]^{8+}$ 1080.7486, $[M+9H]^{9+}$ 960.7774, $[M+10H]^{10+}$ 864.8004, [M] 8637.5421, found *m/z* 1440.6005, 1234.9434, 1080.7013, 960.7348, 864.7620, 8637.5465.

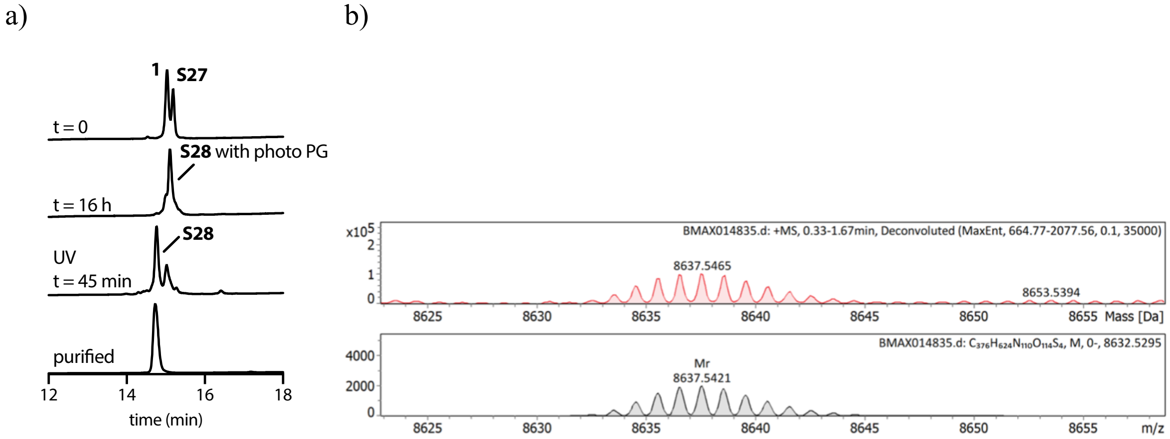

Fig. S44. Analytical data of S28. a) Analytical HPLC trace of the KAHA ligation over time (20 to 95% CH_3CN with 0.1% TFA over 14 min on a Shiseido Capcell Pak MGIII C18, λ = 220 nm). b. HRMS (ESI) of **S28**. Deconvoluted (top) and calculated (bottom).

Synthesis of H_2N-[Cys(Acm)^{5,27,49,67,87,94}, AzK^{41}, photoQ^{116}]-IL-4(1-120)-OH containing isopeptide bond at Leu^{36}-Hse^{37} and Leu^{75}-Hse^{76} S29

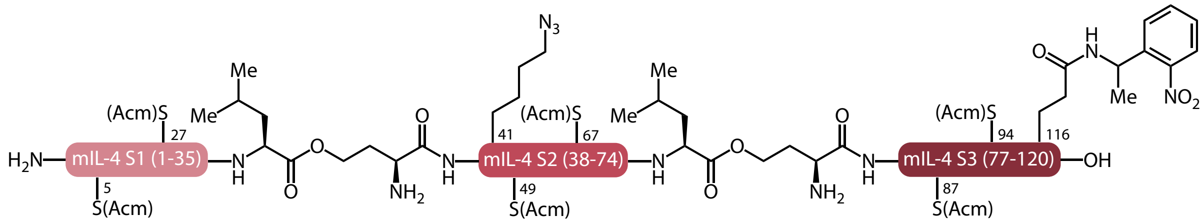

Polypeptide **S28** (41.1 mg, 4.76 µmol, 1.0 equiv) and segment **S23** (33.9 mg, 6.19 µmol, 1.3 equiv) were dissolved in 9:1 DMSO:H_2O with 0.1 M oxalic acid (476 µL, 10 mM). After stirring for 26 h at 60 °C, the resulting gel-like mixture was dissolved with DMSO (1 mL) and purified by preparative RP-HPLC using Osaka Soda Capcell Pak C18 MGIII column (5 µm, 120 Å pore size, 20 mm I.D. x 250 mm) at 60 °C with a gradient of 20 to 70% CH_3CN with 0.1% TFA over 28 min to obtain **S29** (20.4 mg, 31%) as a white fluffy lyophilized powder.

HRMS (ESI) *m/z* calcd for $C_{616}H_{1009}N_{173}O_{190}S_6$ $[M+9H]^{9+}$ 1564.4602, $[M+10H]^{10+}$ 1408.1149, $[M+11H]^{11+}$ 1280.1961, $[M+12H]^{12+}$ 1173.5970, $[M+13H]^{13+}$ 1083.3978, $[M+14H]^{14+}$ 1006.0842, $[M+15H]^{15+}$ 939.0791, $[M+16H]^{16+}$ 880.4496, [M] 14070.3138, found *m/z* 1564.3818, 1408.0442, 1280.1322, 1173.5384, 1083.3435, 1006.0339, 939.0318, 880.4053, 14070.3702.

a) b)

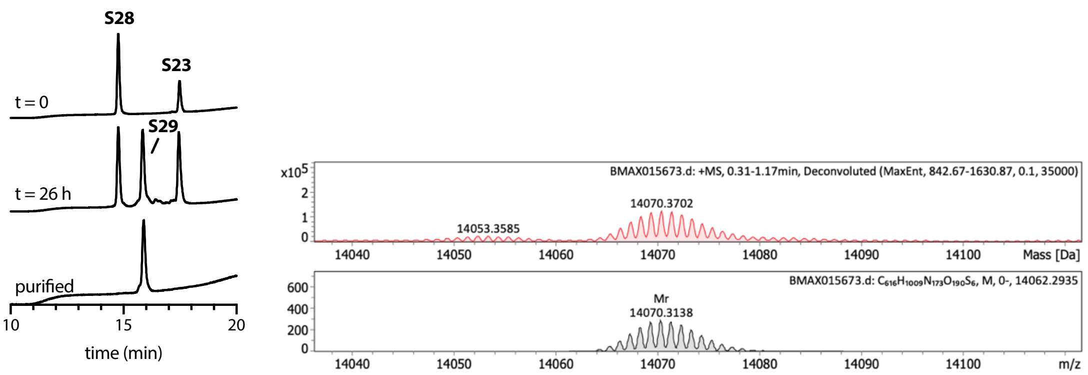

Fig. S45. Analytical data of S29. a) Analytical HPLC trace of the KAHA ligation (20 to 95% CH_3CN with 0.1% TFA over 14 min on a Shiseido Capcell Pak MGIII C18, λ = 220 nm). b) HRMS (ESI) of **S29**. Deconvoluted (top) and calculated (bottom).

Synthesis of H_2N-[Cys(Acm)^{5,27,49,67,87,94}, AzK^{41}, photoQ^{116}]-IL-4(1-120)-OH S30

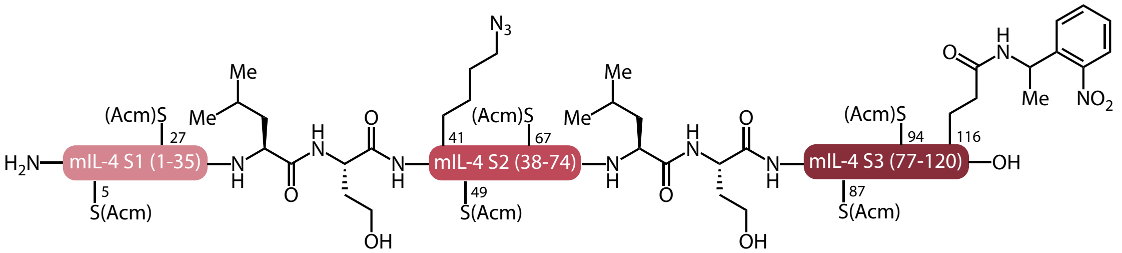

Peptide **S29** (18.7 mg, 1.33 µmol) was dissolved in a buffer containing 50 mM CHES and 6 M Gn·HCl pH 9.5 (6.6 mL, 0.2 mM). After incubating for 2 h at room temperature, the mixture was acidified by adding 50% aq. AcOH and purified by preparative RP-HPLC using Osaka Soda Capcell Pak C18 MGIII column (5 µm, 120 Å pore size, 20 mm I.D. x 250 mm) at 60 °C with a gradient of 10 to 70% CH_3CN with 0.1% TFA over 30 min to obtain **S30** (13.8 mg, 74%) as a white fluffy lyophilized powder.

HRMS (ESI) *m/z* calcd for $C_{616}H_{1009}N_{173}O_{190}S_6$ $[M+7H]^{7+}$ 2011.1610, $[M+8H]^{8+}$ 1759.8918, $[M+9H]^{9+}$ 1564.4602, $[M+10H]^{10+}$ 1408.1149, $[M+11H]^{11+}$ 1280.1961, $[M+12H]^{12+}$ 1173.5970, $[M+13H]^{13+}$ 1083.3978, $[M+14H]^{14+}$ 1006.0842, $[M+15H]^{15+}$ 939.0791, [M] 14070.3138, found *m/z* 2011.0573, 1759.8011, 1408.0427, 1280.1302, 1173.5363, 1083.3418, 1006.0319, 939.0299, 14070.3509.

a) b)

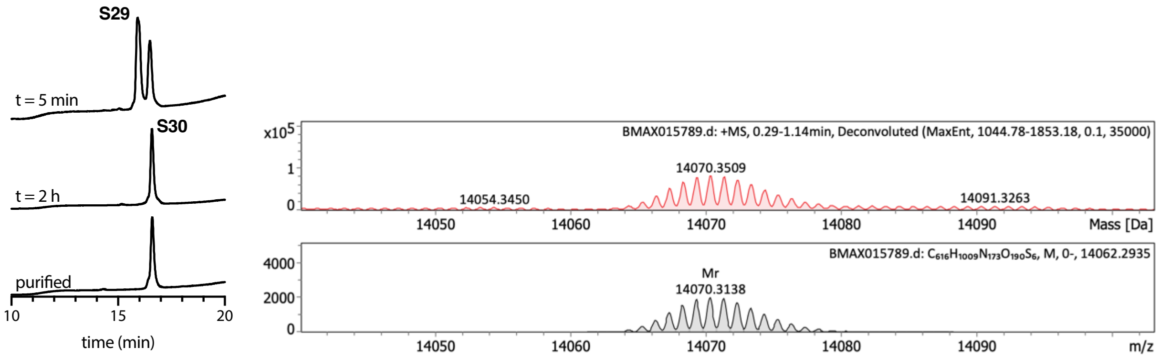

Fig. S46. Analytical data of S30. a) Analytical HPLC trace of the reaction (20 to 95% CH_3CN with 0.1% TFA over 14 min on a Shiseido Capcell Pak MGIII C18, λ = 220 nm). b) HRMS (ESI) of **S30**. Deconvoluted (top) and calculated (bottom).

Synthesis of H_2N-[AzK^{41}, photoQ^{116}]-IL-4(1-120)-OH S31

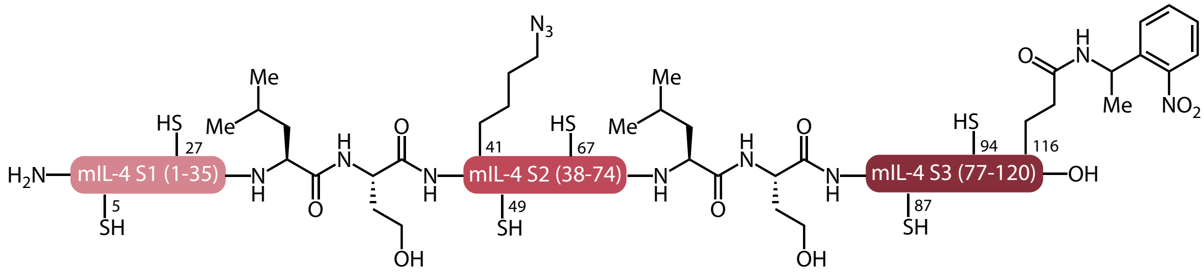

Polypeptide **S30** (13.8 mg, 0.981 µmol) was dissolved in 50% aq. AcOH (3.3 mL) and AgOAc (33.0 mg, 198 µmol) was added. After shaking at 50 °C for 1 h in the dark, the mixture was cooled to room temperature and DTT (33.0 mg, 214 µmol) was added. The resulting precipitate was separated after centrifugation and the precipitate was washed twice with 50% aq. AcOH. The combined supernatant was purified by preparative RP-HPLC using Shiseido Capcell Pak C18 MGIII column (5 µm, 120 Å pore size, 20 mm I.D. x 250 mm) at 60 °C with a gradient of 10 to 70% CH_3CN with 0.1% TFA over 30 min to obtain **S31** (11.2 mg, 84%) as a white fluffy lyophilized powder.

HRMS (ESI) *m/z* calcd for $C_{598}H_{979}N_{167}O_{184}S_6$ $[M+11H]^{11+}$ 1241.4262, $[M+12H]^{12+}$ 1138.0580, $[M+13H]^{13+}$ 1050.5926, $[M+14H]^{14+}$ 975.6222, $[M+15H]^{15+}$ 910.6479, $[M+16H]^{16+}$ 853.7953, [M] 13644.6072, found *m/z* 1241.3847, 1138.0201, 1050.4797, 975.5890, 910.6162, 853.7657, 13643.1514.

a) b)

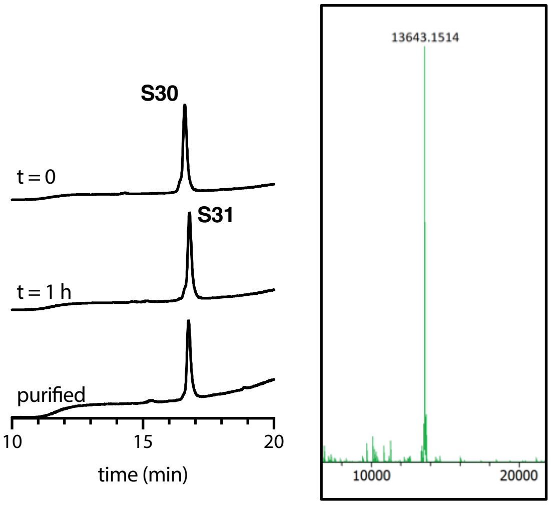

Fig. S47. Analytical data of S31. a) Analytical HPLC trace of Acm deprotection of **S30** (20 to 95% CH_3CN with 0.1% TFA over 14 min on a Shiseido Capcell Pak MGIII C18, λ = 220 nm). b) HRMS (ESI) of **S31** (deconvoluted).

Synthesis of folded H-[AzK^41^, photoQ^116^]-IL-4(1-120)-OH 15

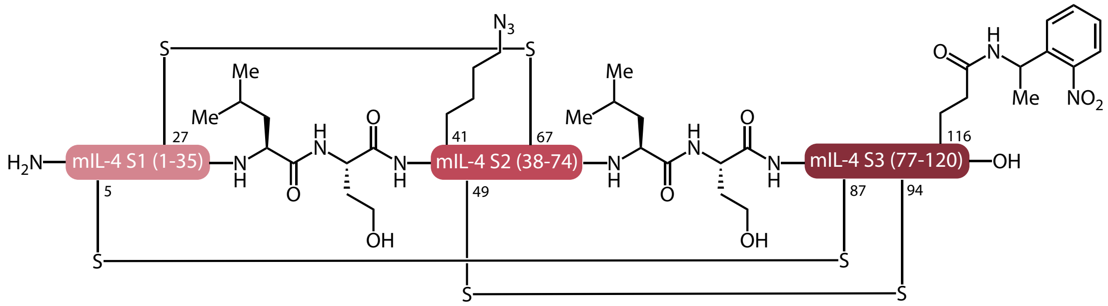

Polypeptide **S31** (4.3 mg, 0.32 µmol) was dissolved in solubilizing buffer 8.6 mL (0.5 mg/mL) containing 4 M Gn·HCl, 50 mM tris and 5 mM EDTA (pH 8.5). This solution was diluted with 43 mL of folding buffer

containing 0.5 M Arg·HCl, 50 mM tris, 2 mM Cys (pH 8.5) and incubated 16 h at room temperature. The mixture was acidified with 6 mL of 50% aq. AcOH and purified by preparative RP-HPLC using Shiseido Proteonavi column (5 µm, 300 Å pore size, 20 mm I.D. x 250 mm) at room temperature with a gradient of 30 to 85% CH_3CN with 0.1% TFA over 30 min to obtain folded photocaged IL-4 **15** (449 µg, 10%, calculated based on BCA assay) as a white fluffy lyophilized powder.

HRMS (ESI) *m/z* calcd for $C_{596}H_{969}N_{165}O_{185}S_6$ $[M+7H]^{7+}$ 1949.3730, $[M+8H]^{8+}$ 1705.8273, [M] 13638.0441, found *m/z* 1949.3021, 1705.8925, 13638.0769.

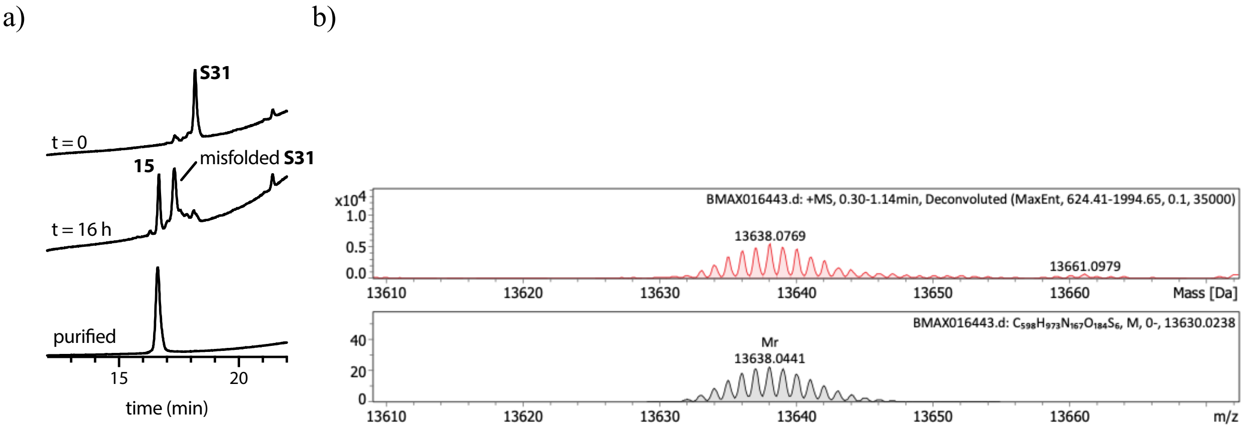

Fig. S48. Analytical data of 15. a) Analytical HPLC of the folding (20 to 95% CH_3CN with 0.1% TFA over 14 min on a Shiseido Proteonavi, λ = 220 nm). b) HRMS (ESI) of **15**. Deconvoluted (top), calculated (bottom).

Synthesis of IL-4-[photoQ^{116}]-30kPEG conjugate at N41 16

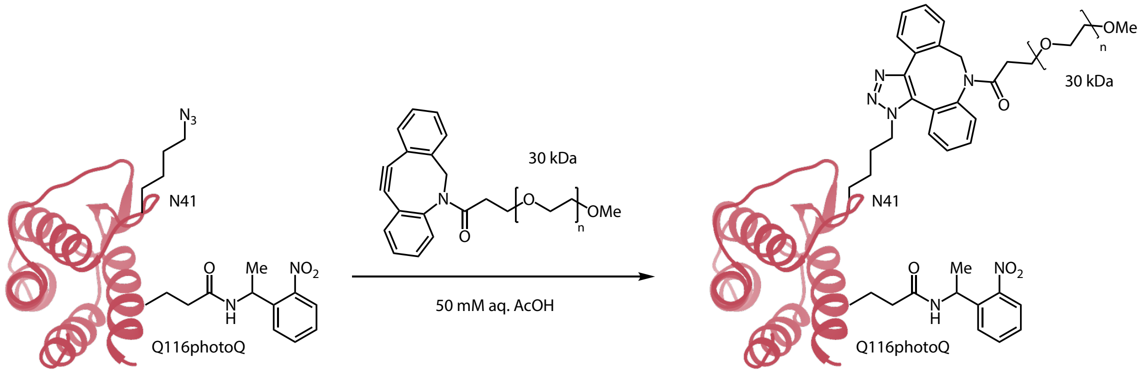

15 **16**

Folded IL-4 containing azidolysine at N41 and photocaged glutamine at Q116 **15** (443 µg, 32.5 nmol, 1.0 equiv) was dissolved in 540 µL of aqueous 50 mM AcOH. To this solution was added DBCO-PEG_{30kDa} (2.9 mg, 0.97 µmol, 3.0 equiv) and the reaction mixture was incubated for 2h at room temperature until the additional portion of DBCO-PEG_{30kDa} (2.9 mg, 0.97 µmol, 3.0 equiv) was added. After incubating for 16 h

at room temperature, the mixture was diluted purified by preparative RP-HPLC using Shiseido Proteonavi (5 µm, 300 Å pore size, 10 mm I.D. x 250 mm) at room temperature with a gradient of 20 to 70% CH_3CN with 0.1% TFA over 30 min to obtain a mixture of DBCO-PEG30kDa and IL-4-photoQ-30kPEG conjugate **16**. This mixture was dissolved in CH_3CN/H_2O = 1:1 + 0.1% TFA and further purified by preparative RP-HPLC using Shiseido Proteonavi (5 µm, 300 Å pore size, 20 mm I.D. x 250 mm) at room temperature with a gradient of 20 to 70% CH_3CN with 0.1% TFA over 30 min to obtain PEGylated photocaged IL-4 **16** (701 µg, 49%, calculated based on BCA assay) as a white fluffy lyophilized powder.

a) b)

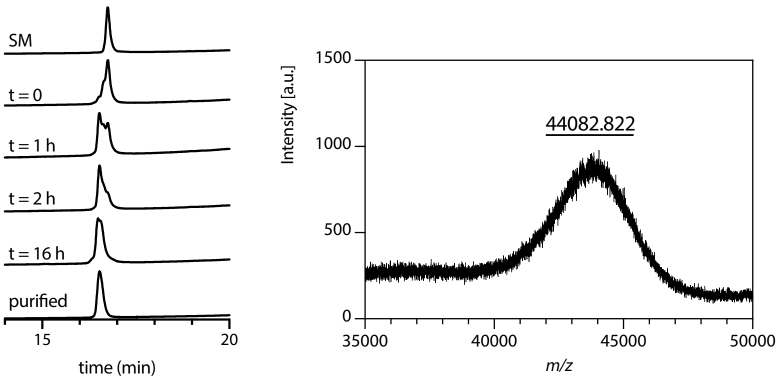

Fig. S49. Analytical data of 16. a) Analytical HPLC of PEGylation (20 to 95% CH_3CN with 0.1% TFA over 14 min on a Shiseido Proteonavi, λ = 220 nm). b) MS (MALDI-TOF) of **16**.

3. NMR spectra

^{1}H-NMR of **S22** (400 MHz, $CDCl_3$)

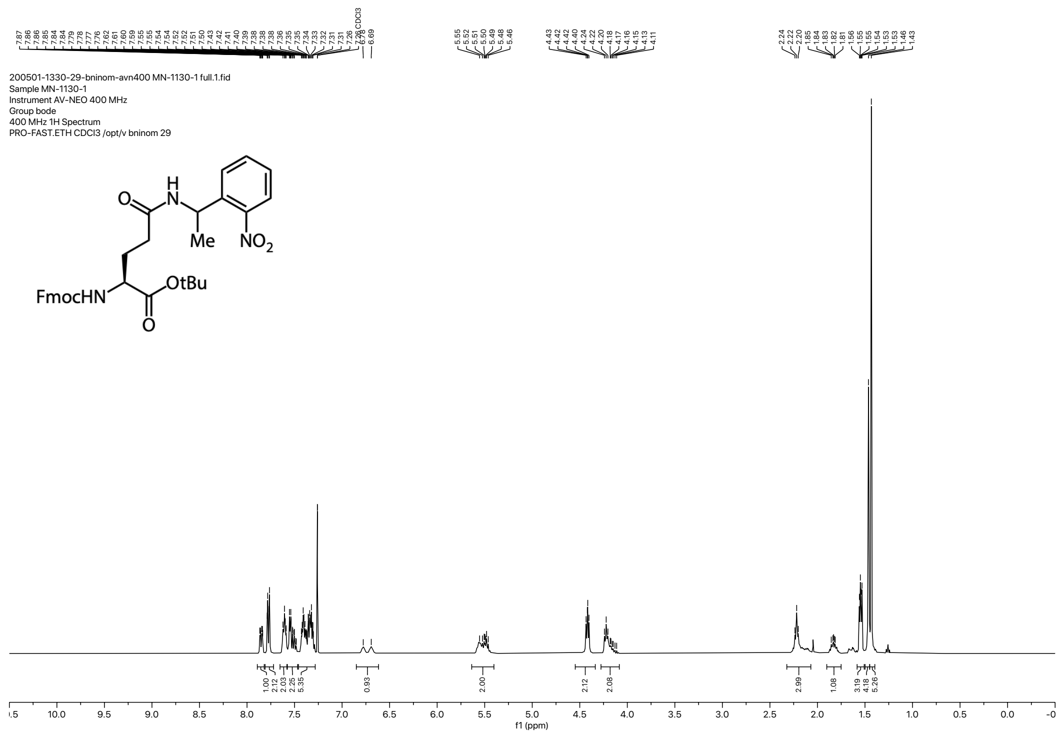

^{13}C-NMR of **S22** (125 MHz, $CDCl_3$)

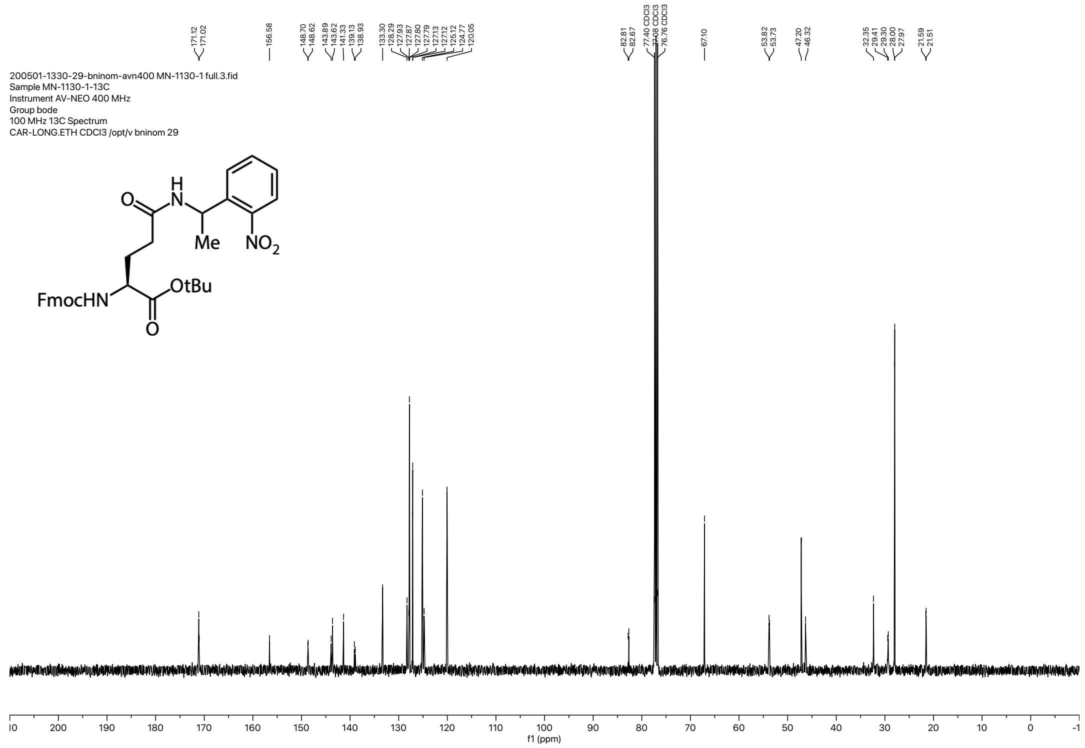

^{1}H-NMR of **13** (400 MHz, DMSO-d_6)

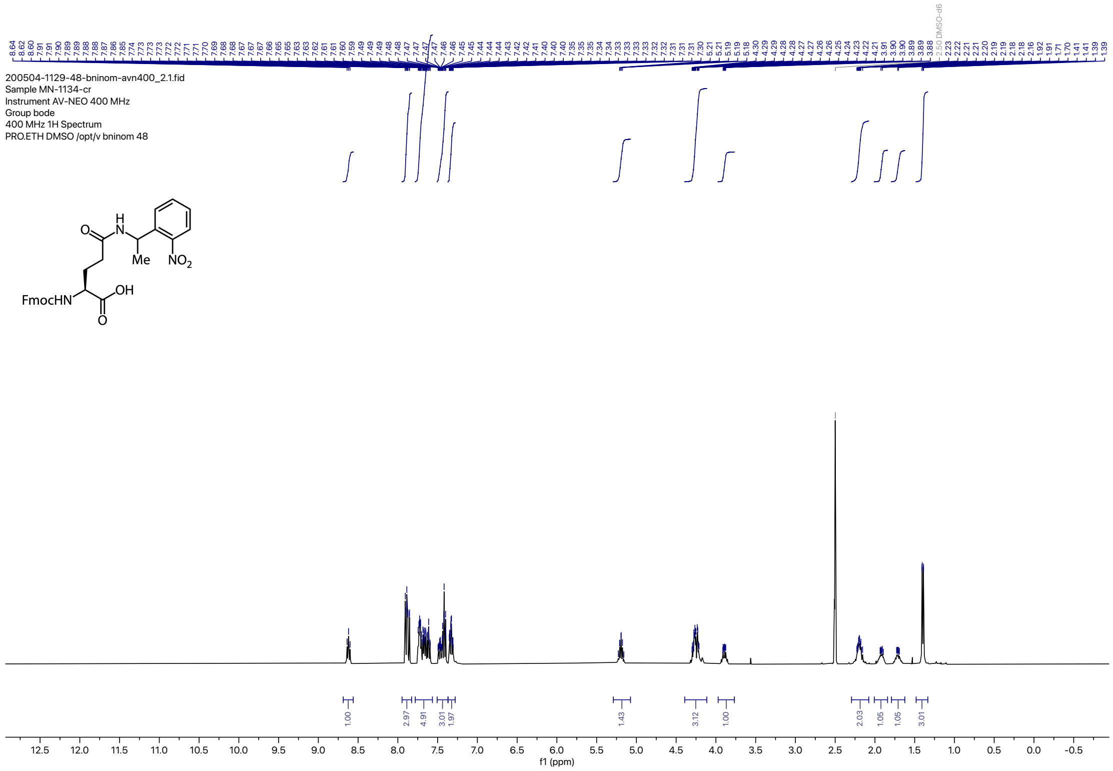

^{13}C-NMR of **13** (100 MHz, DMSO-d_6)

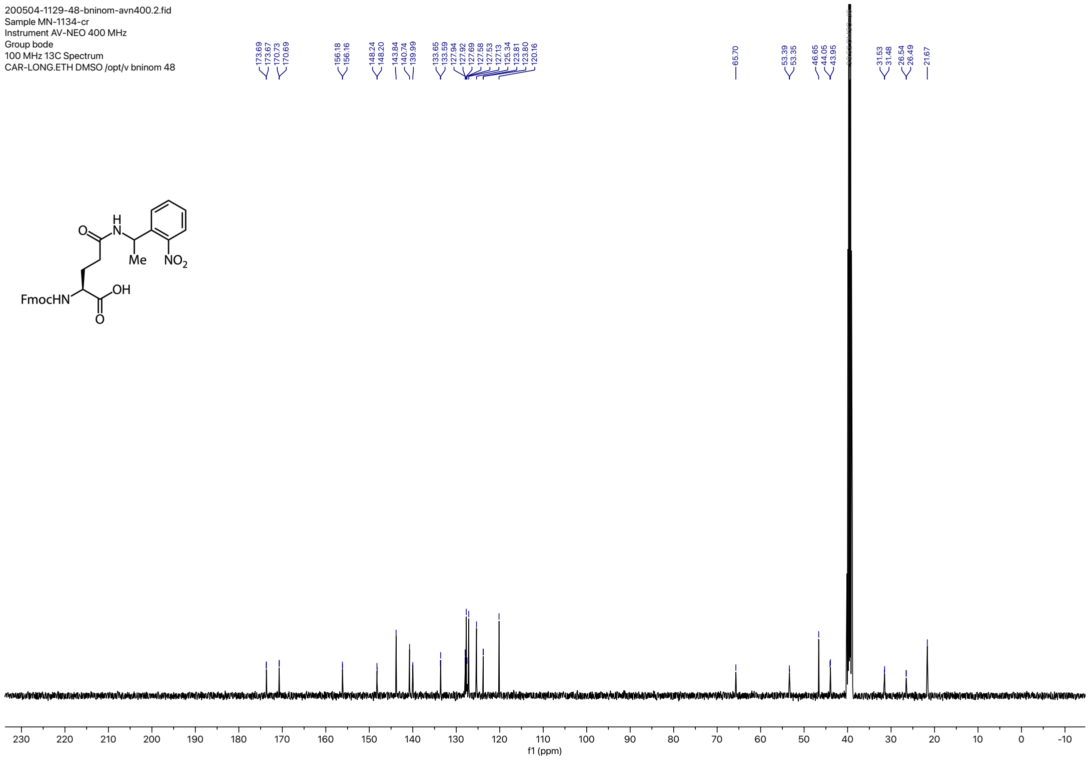

REFERENCES AND NOTES

1. X. Zheng, Y. Wu, J. Bi, Y. Huang, Y. Cheng, Y. Li, Y. Wu, G. Cao, Z. Tian, The use of supercytokines, immunocytokines, engager cytokines, and other synthetic cytokines in immunotherapy. *Cell. Mol. Immunol.* **19**, 192–209 (2022).

2. P. G. Holder, S. A. Lim, C. S. Huang, P. Sharma, Y. S. Dagdas, B. Bulutoglu, J. T. Sockolosky, Engineering interferons and interleukins for cancer immunotherapy. *Adv. Drug Deliv. Rev.* **182**, 114112 (2022).

3. A. M. Levin, D. L. Bates, A. M. Ring, C. Krieg, J. T. Lin, L. Su, I. Moraga, M. E. Raeber, G. R. Bowman, P. Novick, V. S. Pande, C. G. Fathman, O. Boyman, K. C. Garcia, Exploiting a natural conformational switch to engineer an interleukin-2 'superkine'. *Nature* **484**, 529–533 (2012).

4. I. S. Junttila, R. J. Creusot, I. Moraga, D. L. Bates, M. T. Wong, M. N. Alonso, M. M. Suhoski, P. Lupardus, M. Meier-Schellersheim, E. G. Engleman, P. J. Utz, G. C. Fathman, W. E. Paul, C. K. Garcia, Redirecting cell-type specific cytokine responses with engineered interleukin-4 superkines. *Nat. Chem. Biol.* **8**, 990–998 (2012).

5. D.-A. Silva, S. Yu, U. Y. Ulge, J. B. Spangler, K. M. Jude, C. Labão-Almeida, L. R. Ali, A. Quijano-Rubio, M. Ruterbusch, I. Leung, T. Biary, S. J. Crowley, E. Marcos, C. D. Walkey, B. D. Weitzner, F. Pardo-Avila, J. Castellanos, L. Carter, L. Stewart, S. R. Riddell, M. Pepper, G. J. L. Bernardes, M. Dougan, K. C. Garcia, D. Baker, De novo design of potent and selective mimics of IL-2 and IL-15. *Nature* **565**, 186–191 (2019).

6. J. T. Sockolosky, E. Trotta, G. Parisi, L. Picton, L. L. Su, A. C. Le, A. Chhabra, S. L. Silveria, B. M. George, I. C. King, M. R. Tiffany, K. Jude, L. V. Sibener, D. Baker, J. A. Shizuru, A. Ribas, J. A. Bluestone, K. C. Garcia, Selective targeting of engineered T cells using orthogonal IL-2 cytokine-receptor complexes. *Science* **359**, 1037–1042 (2018).

7. H. Yang, U. Y. Ulge, A. Quijano-Rubio, Z. J. Bernstein, D. R. Maestas, J.-H. Chun, W. Wang, J.-X. Lin, K. M. Jude, S. Singh, B. T. Orcutt-Jahns, P. Li, J. Mou, L. Chung, Y.-H. Kuo, Y. H. Ali, A. S. Meyer, W. L. Grayson, N. M. Heller, K. C. Garcia, W. J. Leonard, D.-A. Silva, J. H.

Elisseeff, D. Baker, J. B. Spangler, Design of cell-type-specific hyperstable IL-4 mimetics via modular de novo scaffolds. *Nat. Chem. Biol.* **19**, 1127–1137 (2023).

8. S. L. LaPorte, S. Z. Juo, J. Vaclavikova, L. A. Colf, X. Qi, N. M. Heller, A. D. Keegan, C. K. Garcia, Molecular and structural basis of cytokine receptor pleiotropy in the interleukin-4/13 system. *Cell* **132**, 259–272 (2008).

9. M. Akaiwa, B. Yu, R. Umeshita-Suyama, N. Terada, H. Suto, T. Koga, K. Arima, S. Matsushita, H. Saito, H. Ogawa, M. Furue, N. Hamasaki, K. Ohshima, K. Izuhara, Localization of human interleukin 13 receptor in non-haematopooetic cells. *Cytokine* **13**, 75–84 (2001).

10. I. S. Junttila, Tuning the cytokine responses: An update on interleukin (IL)-4 and IL-13 receptor complexes. *Front. Immunol.* **9**, 888 (2018).

11. J. Woytschak, N. Keller, C. Krieg, D. Impellizzieri, R. W. Thompson, T. A. Wynn, A. S. Zinkernagel, O. Boyman, Type 2 interleukin-4 receptor signaling in neutrophils antagonizes their expansion and migration during infection and inflammation. *Immunity* **45**, 172–184 (2016).

12. K. Nelms, A. D. Keegan, J. Zamorano, J. J. Ryan, W. E. Paul, The IL-4 receptor: Signaling mechanisms and biologic functions. *Annu. Rev. Immunol.* **17**, 701–738 (1999).

13. D. Impellizzieri, F. Ridder, M. E. Raeber, C. Egholm, J. Woytschak, A. G. A. Kolios, D. F. Legler, O. Boyman, Interleukin-4 receptor engagement in human neutrophils impairs their migration and extracellular trap formation. *J. Allergy Clin. Immunol.* **144**, 267–279.e4 (2019).

14. C. A. Akdis, P. D. Arkwright, M.-C. Brüggen, W. Busse, M. Gadina, E. Guttman-Yassky, K. Kabashima, Y. Mitamura, L. Vian, J. Wu, O. Palomares, Type 2 immunity in the skin and lungs. *Allergy* **75**, 1582–1605 (2020).

15. N. Kruse, B. J. Shen, S. Arnold, H. P. Tony, T. Müller, W. Sebald, Two distinct functional sites of human interleukin 4 are identified by variants impaired in either receptor binding or receptor activation. *EMBO J.* **12**, 5121–5129 (1993).

16. A.-L. Andrews, J. W. Holloway, S. T. Holgate, D. E. Davies, IL-4 receptor α is an important modulator of IL-4 and IL-13 receptor binding: Implications for the development of therapeutic targets. *J. Immunol.* **176**, 7456–7461 (2006).

17. V. Duppatla, M. Gjorgjevikj, W. Schmitz, H. M. Hermanns, C. M. Schäfer, M. Kottmair, T. Müller, W. Sebald, IL-4 analogues with site-specific chemical modification at position 121 inhibit IL-4 and IL-13 biological activities. *Bioconjug. Chem.* **25**, 52–62 (2014).

18. K. K. Rathinam, J. J. Abraham, T. M. Vijayakumar, Dupilumab in the treatment of moderate to severe asthma: An evidence-based review. *Curr. Ther. Res.* **91**, 45–51 (2019).

19. A. T. U. Din, I. Malik, D. Arshad, A. T. U. Din, Dupilumab for atopic dermatitis: The silver bullet we have been searching for? *Cureus* **12**, e7565 (2020).

20. M. J. Gooderham, H. C. Hong, P. Eshtiaghi, K. A. Papp, Dupilumab: A review of its use in the treatment of atopic dermatitis. *J. Am. Acad. Dermatol.* **78**, S28–S36 (2018).

21. C.-C. Chiang, W.-J. Cheng, M. Korinek, C.-Y. Lin, T.-L. Hwang, Neutrophils in psoriasis. *Front. Immunol.* **10**, 2376 (2019).

22. W.-M. Wang, H.-Z. Jin, Role of neutrophils in psoriasis. *J. Immunol. Res.* **2020**, 1–6 (2020).

23. F. Grigolato, C. Egholm, D. Impellizzieri, P. Arosio, O. Boyman, Establishment of a scalable microfluidic assay for characterization of population-based neutrophil chemotaxis. *Allergy* **75**, 1382–1393 (2020).

24. D. Impellizzieri, C. Egholm, A. Valaperti, O. Distler, O. Boyman, Patients with systemic sclerosis show phenotypic and functional defects in neutrophils. *Allergy* **77**, 1274–1284 (2022).

25. C. Egholm, A. Özcan, D. Breu, O. Boyman, Type 2 immune predisposition results in accelerated neutrophil aging causing susceptibility to bacterial infection. *Sci. Immunol.* **7**, eabi9733 (2022).

26. R. Martin, Interleukin 4 treatment of psoriasis: Are pleiotropic cytokines suitable therapies for autoimmune diseases? *Trends Pharmacol. Sci.* **24**, 613–616 (2003).

27. K. Ghoreschi, P. Thomas, S. Breit, M. Dugas, R. Mailhammer, W. van Eden, R. van der Zee, T. Biedermann, J. Prinz, M. Mack, U. Mrowietz, E. Christophers, D. Schlöndorff, G. Plewig, C. A. Sander, M. Röcken, Interleukin-4 therapy of psoriasis induces Th2 responses and improves human autoimmune disease. *Nat. Med.* **9**, 40–46 (2003).

28. J. W. Bode, Chemical protein synthesis with the α-ketoacid–hydroxylamine ligation. *Acc. Chem. Res.* **50**, 2104–2115 (2017).

29. C. E. Murar, M. Ninomiya, S. Shimura, U. Karakus, O. Boyman, J. W. Bode, Chemical synthesis of interleukin-2 and disulfide stabilizing analogues. *Angew. Chem. Int. Ed. Engl.* **59**, 8425–8429 (2020).

30. T. D. Mueller, J.-L. Zhang, W. Sebald, A. Duschl, Structure, binding, and antagonists in the IL-4/IL-13 receptor system. *Biochim. Biophys. Acta Mol. Cell Res.* **1592**, 237–250 (2001).

31. C. Carr, S. Aykent, N. Kimack, A. Levine, Disulfide assignments in recombinant mouse and human interleukin 4. *Biochemistry* **30**, 1515–1523 (1991).

32. N. Kruse, T. Lehrnbecher, W. Sebald, Site-directed mutagenesis reveals the importance of disulfide bridges and aromatic residues for structure and proliferative activity of human Interleukin-4. *FEBS Lett.* **286**, 58–60 (1991).

33. T. Durek, V. Y. Torbeev, S. B. H. Kent, Convergent chemical synthesis and high-resolution x-ray structure of human lysozyme. *Proc. Natl. Acad. Sci. U.S.A.* **104**, 4846–4851 (2007).

34. T. Wöhr, F. Wahl, A. Nefzi, B. Rohwedder, T. Sato, X. Sun, M. Mutter, Pseudo-prolines as a solubilizing, structure-disrupting protection technique in peptide synthesis. *J. Am. Chem. Soc.* **118**, 9218–9227 (1996).

35. C. J. White, J. W. Bode, PEGylation and dimerization of expressed proteins under near equimolar conditions with potassium 2-pyridyl acyltrifluoroborates. *ACS Cent. Sci.* **4**, 197–206 (2018).

36. G. N. Boross, D. Schauenburg, J. W. Bode, Chemoselective derivatization of folded synthetic insulin variants with potassium acyltrifluoroborates (KATs). *Helv. Chim. Acta* **102**, e1800214 (2019).

37. E. J. Hsu, X. Cao, B. Moon, J. Bae, Z. Sun, Z. Liu, Y.-X. Fu, A cytokine receptor-masked IL2 prodrug selectively activates tumor-infiltrating lymphocytes for potent antitumor therapy. *Nat. Commun.* **12**, 2768 (2021).

38. X. Cao, Y. Liang, Z. Hu, H. Li, J. Yang, E. J. Hsu, J. Zhu, J. Zhou, Y.-X. Fu, Next generation of tumor-activating type I IFN enhances anti-tumor immune responses to overcome therapy resistance. *Nat. Commun.* **12**, 5866 (2021).

39. J. Guo, Y. Liang, D. Xue, J. Shen, Y. Cai, J. Zhu, Y.-X. Fu, H. Peng, Tumor-conditional IL-15 pro-cytokine reactivates anti-tumor immunity with limited toxicity. *Cell Res.* **31**, 1190–1198 (2021).

40. Y. Zhao, Y.-Q. Xie, S. V. Herck, S. Nassiri, M. Gao, Y. Guo, L. Tang, Switchable immune modulator for tumor-specific activation of anticancer immunity. *Sci. Adv.* **7**, eabg7291 (2021).

41. D. Ramesh, R. Wieboldt, A. P. Billington, B. K. Carpenter, G. P. Hess, Photolabile precursors of biological amides: Synthesis and characterization of caged o-nitrobenzyl derivatives of glutamine, asparagine, glycinamide, and .gamma.-aminobutyramide. *J. Org. Chem.* **58**, 4599–4605 (1993).

42. L. Awad, N. Jejelava, R. Burai, H. A. Lashuel, A new caged-glutamine derivative as a tool to control the assembly of glutamine-containing amyloidogenic peptides. *Chembiochem* **17**, 2353–2360 (2016).

43. K. R. Moulton, A. Sadiki, B. N. Koleva, L. J. Ombelets, T. H. Tran, S. Liu, B. Wang, H. Chen, E. Micheloni, P. J. Beuning, G. A. O'Doherty, Z. S. Zhou, Site-specific reversible protein and peptide modification: Transglutaminase-catalyzed glutamine conjugation and bioorthogonal light-mediated removal. *Bioconjug. Chem.* **30**, 1617–1621 (2019).

44. S. N. S. Alconcel, A. S. Baas, H. D. Maynard, FDA-approved poly(ethylene glycol)– protein conjugate drugs. *Polym. Chem.* **2**, 1442–1448 (2011).

45. L. H. Belén, C. de Oliveira Rangel-Yagui, J. F. B. Lissabet, B. Effer, M. Lee-Estevez, A. Pessoa, R. L. Castillo, J. G. Farías, From synthesis to characterization of site-selective PEGylated proteins. *Front. Pharmacol.* **10**, 1450 (2019).

46. M. F. Debets, S. S. van Berkel, S. Schoffelen, F. P. J. T. Rutjes, J. C. M. van Hest, F. L. van Delft, Aza-dibenzocyclooctynes for fast and efficient enzyme PEGylation via copper-free (3+2) cycloaddition. *Chem. Commun.* **46**, 97–99 (2009).

47. B. Zhang, H. Xu, J. Chen, Y. Zheng, Y. Wu, L. Si, L. Wu, C. Zhang, G. Xia, L. Zhang, D. Zhou, Development of next generation of therapeutic IFN-α2b via genetic code expansion. *Acta Biomater.* **19**, 100–111 (2015).

48. C. L. Semerad, F. Liu, A. D. Gregory, K. Stumpf, D. C. Link, G-CSF is an essential regulator of neutrophil trafficking from the bone marrow to the blood. *Immunity* **17**, 413–423 (2002).

49. D. Richter, I. Moraga, H. Winkelmann, O. Birkholz, S. Wilmes, M. Schulte, M. Kraich, H. Kenneweg, O. Beutel, P. Selenschik, D. Paterok, M. Gavutis, T. Schmidt, C. K. Garcia, T. D. Müller, J. Piehler, Ligand-induced type II interleukin-4 receptor dimers are sustained by rapid re-association within plasma membrane microcompartments. *Nat. Commun.* **8**, 15976 (2017).

50. K. Kurgonaite, H. Gandhi, T. Kurth, S. Pautot, P. Schwille, T. Weidemann, C. Bökel, Essential role of endocytosis for interleukin-4-receptor-mediated JAK/STAT signalling. *J. Cell Sci.* **128**, 3781–3795 (2015).

51. H. Gandhi, R. Worch, K. Kurgonaite, M. Hintersteiner, P. Schwille, C. Bökel, T. Weidemann, Dynamics and interaction of interleukin-4 receptor subunits in living cells. *Biophys. J.* **107**, 2515–2527 (2014).

52. M. C. Pirrung, W. H. Pieper, K. P. Kaliappan, M. R. Dhananjeyan, Combinatorial discovery of two-photon photoremovable protecting groups. *Proc. Natl. Acad. Sci. U.S.A.* **100**, 12548–12553 (2003).

53. M. J. Hansen, W. A. Velema, M. M. Lerch, W. Szymanski, B. L. Feringa, Wavelength-selective cleavage of photoprotecting groups: Strategies and applications in dynamic systems. *Chem. Soc. Rev.* **44**, 3358–3377 (2015).

54. T. J. Harmand, C. E. Murar, J. W. Bode, Protein chemical synthesis by α-ketoacid–hydroxylamineligation. *Nat. Prot.* **11**, 1130–1147 (2016).

55. C. P. Salerno, D. Magde, A. P. Patron, Enzymatic Synthesis of Caged NADP Cofactors: AqueousNADP Photorelease and Optical Properties. *J. Org. Chem.* **65**, 3971–3981 (2000).
